# Reflections on co-producing an obesity-prevention toolkit for Islamic Religious Settings: a qualitative process evaluation

**DOI:** 10.1186/s12966-024-01610-w

**Published:** 2024-06-12

**Authors:** Jennifer Hall, Rukhsana Rashid, Abida Rafiq, Kiran Fatima, Sally E. Barber, Sufyan Abid Dogra

**Affiliations:** 1 Bradford Institute for Health Research, Bradford Teaching Hospitals NHS Foundation Trust, Bradford Royal Infirmary, Duckworth Lane, Bradford, BD9 6RJ UK; 2 Faculties of Life Sciences and Health Studies, University of Bradford, Richmond Road, Bradford, BD7 1DP UK; 3 Core20Plus5, Craven Health and Care Partnership, NHS West Yorkshire Integrated Care Board, Scorex House, Bradford District &, Bradford, BD1 4AS UK; 4 Faculty of Management, Law and Social Sciences, University of Bradford, Richmond Road, Bradford, BD7 1DP UK

**Keywords:** Islamic Religious Settings, Physical activity, Diet, Behaviour change, Obesity, Muslim, South Asian, Co-production, Children

## Abstract

**Background:**

Islamic leaders, staff, and Muslim parents in the UK are supportive of healthy lifestyle intervention delivery through Islamic Religious Settings. Such interventions are necessary given high obesity rates in British South Asian (40%) compared to White British (32%) children of equivalent age. Co-production can facilitate the development of culturally appropriate health interventions, however it can be theoretically and practically challenging, and evaluation of co-production within an Islamic Religious Setting context is lacking. The aim of this study was to examine the feasibility and acceptability of taking a co-production approach to develop an obesity-prevention toolkit for Islamic Religious Settings.

**Methods:**

An obesity-prevention toolkit for use in Islamic Religious Settings, incorporating physical activity, healthy diet, and organisational change, has been co-produced to be evidence-informed and contextually relevant. A qualitative process evaluation was employed to examine experiences of co-production. Semi-structured interviews (*n* = 15) and a focus group (*n* = 5) were conducted with toolkit co-production stakeholders, e.g., subject experts, an Islamic scholar, and Islamic Religious Setting staff. Transcripts were analysed inductively using reflexive thematic analysis.

**Results:**

The analysis revealed four major themes regarding stakeholders' experiences of co-producing a childhood obesity-prevention toolkit for Islamic Religious Settings. These themes are: (1) attitudes towards obesity-prevention through Islamic Religious Settings, (2) benefits of co-production including capacity building and ownership (3) negotiating involvement, power, and perspectives within the co-production process, and (4) the complexities of effective communication in co-production.

**Conclusion:**

This study adds to the evidence-base in support of delivering health promotion through faith settings. Taking a co-production approach to develop an obesity-prevention toolkit for Islamic Religious Settings provided benefit to the toolkit product and local stakeholders. The toolkit is currently being implemented across Bradford, UK and there is potential to adapt the toolkit to other geographical contexts, and for evaluating effectiveness for preventing obesity in British Muslim families.

**Supplementary Information:**

The online version contains supplementary material available at 10.1186/s12966-024-01610-w.

## Background

Childhood obesity is one of the most severe health challenges of the 21^st^ century, with one in five children in the UK leaving primary school living with obesity [30]. Childhood obesity often tracks into adulthood and children with obesity are more likely to develop diabetes and cardiovascular diseases at a younger age [31]. The South Asian ethnic minority has higher rates of childhood obesity compared to their White British counterparts. For example, in Bradford UK, 40% of South Asian children live with overweight or obesity compared to 32% of White British children, based on standard UK cutoff points for age and sex [9].

Ethnic inequalities in obesity result from a combination of metabolic, socioeconomic, cultural, and behavioural factors [17, 40]. Being South Asian is a risk factor for having the thin-fat phenotype [35]. Additionally, South Asian families are geographically concentrated in areas of socioeconomic deprivation, which tend to be highly populated with fast food outlets and have limited availability of healthy food options ([7, 15, 33].

Health promotion interventions tend to be aimed at individuals, such as educational approaches to diet and physical activity [26], whilst the systemic causes of obesity point to a need for multifaceted interventions that target socio-political, environmental, and cultural change [8, 13]. Additionally, whilst individual-level interventions have some effectiveness at reducing obesity [32], they can perpetuate inequalities as they tend to underrepresent ethnic minority groups and are often not culturally tailored [5, 19]. Examining context is key to developing tailored interventions and understanding whether and how they work [24].

Governmental and research bodies have acknowledged the potential role that places of worship have in promoting health and have noted local trusted faith groups as promising delivery partners [18, 28, 29]. Of UK Muslims, over 92% are from an ethnic minority background, over two-thirds are South Asian [22] and a large proportion of Muslim children attend madrasa, i.e. after-school Islamic supplementary educational institutions in the UK. A recent scoping review of health-promotion interventions in Islamic Religious Settings (IRSs) demonstrated that Muslim opinion makers play an important role in promoting health to their networks and congregations [28]. A qualitative study highlighted that IRS leaders, madrasa teachers and parents of children attending madrasas believe that it is possible to deliver a childhood obesity-prevention intervention through IRSs and recommend a toolkit approach to facilitate intervention delivery [9]. Despite this, no known studies have attempted to integrate an obesity-prevention intervention into the existing IRS educational environment in the UK. Smoking cessation interventions have been successfully delivered through IRS ([1]. However, these interventions were developed by researchers *for* IRSs, rather than *with* IRSs [11].

Co-production of public health interventions involves a shift of power from professionals and authorities to communities and service users [23]. There is no single agreed-upon definition of co-production, however, a key feature and value of co-production is that it includes equitable partnership between different stakeholders (professionals, researchers, end users) where all stakeholders can make meaningful contributions throughout design, development and delivery processes [41]. Through involving IRS stakeholders, co-production can facilitate the development of culturally appropriate health interventions and increase IRS ownership of delivering such interventions [12]. It is important to understand the process and acceptability of co-producing health interventions to advance knowledge about how best to design co-production processes in different contexts to maximise benefits, such as increased motivation/ownership for supporting delivery of the intervention and/or engaging in the target behaviour [14]. This study aimed to examine the barriers and enablers to co-producing an IRS childhood obesity-prevention toolkit from the perspectives of co-production stakeholders.

## Methods

A qualitative process evaluation was undertaken to understand the benefits and challenges of co-producing a toolkit for childhood obesity-prevention within IRS. The study was underpinned by a constructivist approach (relativist ontological assumptions and subjectivist epistemological assumptions) [27] to centre stakeholders’ experiences. Presentation of recruitment, data collection and analysis are aligned with the Consolidated criteria for Reporting Qualitative Research [38], see Additional file 1. The study received ethical approval from Leeds Beckett University in March 2020 (ref: 69870) and was transferred to and approved by University of Bradford in May 2021 (ref: E888).

### Study context

This study took place in Bradford, a city in the Northern England, which is the 5^th^ most income deprived and 6^th^ most employment deprived local government in the UK [3]. Over 40% of children in Bradford are of South Asian origin [22]. There are 120 registered IRSs (mosques, madrasa, women’s circles, community organisations affiliated with IRSs) in Bradford and 91% of Muslim primary school children attend madrasa after school [8].

In 2019, Bradford Council, the Bradford Council for Mosques, and Born in Bradford formed a partnership to investigate the opportunities for working with IRSs, particularly madrasas, to support healthier behaviours and impact structural and social change for better health. This work was one of five Childhood Obesity Trailblazer Programmes across the UK, funded for three years (2019–22) by the Local Government Association. A key objective as part of the Bradford Trailblazer Programme was to develop a culturally appropriate obesity-prevention toolkit for IRS use. The toolkit primarily aimed to address the level of the Islamic Religious Setting and support organisational change within IRS (see Additional file 2 for the toolkit).

The toolkit audience was IRS staff, management, and parents of children attending madrasa; through toolkit implementation we aimed to increase the capability, opportunity, and motivation of IRSs to promote healthy behaviours amongst children and families. The toolkit contents targeted three components: IRSs as healthy places, physical activity, and healthy diet. The healthy places component focused on organisational behaviour change, such as establishing a local place-based group in IRSs, and collaborating with external agencies, to facilitate the promotion of healthy behaviours. The physical activity and healthy diet components presented sessions that can be delivered by IRS staff or external experts to children (and families) in group settings. Islamic narrative was integrated throughout the toolkit to illustrate the compatibility of healthy behaviours with Islamic ethos and increase the acceptability of health promotion activity within the madrasa. See Additional file 3 for the TIDieR checklist.

#### Process of coproducing the obesity-prevention toolkit

The obesity trailblazer leadership team oversaw the toolkit co-production process. A toolkit development group was established to co-design the toolkit content. This group consisted of physical activity, healthy diet, and behaviour change experts, applied health researchers, educators and practitioners, and an Islamic scholar (i.e. someone who studies Islam) with significant lived experience of IRSs in Bradford. The leadership team tasked the toolkit development group with creating draft session plans for select sessions. Following this, the multisectoral stakeholders formed three small groups, based on their expertise, to create complete drafts of the organisational behaviour change, physical activity, and healthy diet toolkit components. Alongside this, a Community Engagement Manager (CEM) funded by the Childhood Obesity Trailblazer Programme, was tasked with facilitating 10 place-based groups affiliated with IRSs in Bradford and enable them to review and amend toolkit contents. The initial aim was to deliver face-to-face toolkit test and learn sessions within IRSs, but this was not possible due to COVID-19 restrictions at the time of development. Thus, place-based groups provided feedback through online and small face-to-face meetings, which was used to refine toolkit material. The revised contents were taken back to place-based groups for review to ensure their feedback was incorporated as they intended.

This study took place in Spring 2021; the toolkit content had largely been produced but the overall toolkit had not been finalised. Following the study, cross-sectorial stakeholder workshops were delivered to attain wider input prior to the toolkit’s finalisation and use.

### Sampling and recruitment

A purposive sampling technique was employed. Participants were selected based on their involvement in developing the obesity-prevention toolkit. All toolkit development group members were invited to participate in an interview (*n* = 16). Given the large number of stakeholders involved in the place-based groups (60 across 10 place-based groups) a sub-sample of these stakeholders were selected to take part in a focus group. IRS stakeholders of different genders, from different IRSs, with different roles, and with differing levels of involvement in the toolkit development, were invited to participate in the focus group. Recruitment was facilitated by the CEM, who shared the study information and a link to a survey (see 2.3) via email, which collected participants’ informed consent.

### Data collection methods

A short survey collecting information related to gender, date of birth, home postcode, ethnicity, highest educational qualification, employer, and job role was completed by all participants to enable characterisation of the sample. Qualitative interviews were employed to gain an in-depth understanding of toolkit development group stakeholders’ experiences. Focus groups were utilised to understand diverse experiences, by providing an opportunity for stakeholders from different IRS contexts to compare and contrast their experiences. The qualitative topic guide was developed in partnership with the obesity trailblazer project lead, informed by the study aims and the Theoretical Framework for Acceptability (TFA) which outlines seven component constructs: affective attitude, burden, perceived effectiveness, ethicality, coherence, opportunity costs, and self-efficacy [34]. Drawing on these constructs, questions sought to understand stakeholders’ experiences of being part of the process, and the mechanisms and contextual factors underpinning implementation and impact of co-producing the toolkit. See Additional file 4 for the interview topic guide. Data collection was facilitated by a researcher (JH or MHH) and took place face-to-face (in the workplace) or remotely, depending on participant preference, with no one else present. JH is a non-Muslim qualitative researcher with significant experience in co-production of complex interventions. MHH is a Muslim woman who, at the time of the study, was a Masters in Public Health student. Interviews/focus groups lasted approximately 45 minutes. All qualitative data was audio recorded and transcribed verbatim by a researcher using Otter.ai (www.otter.ai), and anonymised.

### Data analysis

Survey data was summarised using descriptive statistics. Qualitative data was analysed using reflexive thematic analysis [4]. One researcher (JH) open-coded all transcripts and developed initial themes using NVivo. A second researcher (RR) read all the transcripts, reviewed the themes crafted by the primary researcher, and added their thoughts and reflections, including suggestions for additional sub-themes and different ways of organising and presenting the data. A critical friend approach, whereby other researchers provide a “theoretical sounding board” to explore multiple and alternative explanations, and being reflective about the subjective values of the researcher(s), helped maintain rigour [36]. The researchers met to discuss different interpretations, and the themes were revised and agreed.

## Results

Twenty participants took part in an interview (*n* = 15) or focus group (*n* = 5). One toolkit development group member was unavailable for an interview. Of the 15 interviewees, nine were women, eight were White British, and seven South Asian (Pakistani). They worked in roles in the National Health Service, local government, higher education, religious institutions, and their own businesses. The five IRS focus group participants (two women) all identified as British South Asian Muslims and held different volunteer positions in IRSs across Bradford. Participants’ self-reported role in co-producing the obesity-prevention toolkit included: 11 experts by experience, two behaviour change academic experts and one practitioner, three physical activity practitioners and one academic expert, three project management and leadership stakeholders, two public health practitioners, two community engagement practitioners, one healthy eating practitioner and one academic expert, a governance and safeguarding practitioner, an Islamic scholar, and a youth work/education practitioner. Qualitative data analysis produced four overarching themes related to stakeholders’ experiences of co-producing a childhood obesity-prevention toolkit for IRSs. The themes include (1) attitudes towards obesity-prevention through IRSs, (2) the power of co-production, (3) negotiating involvement, power, and perspectives within co-production and (4) complexities of effective communication within co-production.

### Theme one: attitudes towards obesity-prevention through IRSs

The stakeholders were positive about the idea of the intervention. Nevertheless, the interviewees described anticipated challenges related to delivering the sessions and activities described within the toolkit, and recommendations for ensuring successful and sustainable implementation.

#### Toolkit approach

There was recognition that the toolkit approach supported capacity building within IRSs, which would facilitate organisational changes and session delivery focused on health promotion:

*“We expect our leaders to have skills in everything, you know, around terrorism, around hospital care, sexual health, and how do we expect them to have all these skills, without the appropriate training? So, I thought it was a really good idea. I think manualising this process meant that we would bring some more professionalism into the IRS settings” (Participant 11)*

Interviewees valued the flexibility the toolkit approach offered, as it allowed IRSs to choose and adapt sessions as appropriate for their setting:

*“It’s quite a valuable asset in our community. Because if there's something that doesn't really work for your sort of setting, then there's so much to choose from… some settings don't have a lot of space compared to others. And sort of each madrasa will do things differently... So it wasn't sort of tailored to just one madrasa” (IRS focus group participant)*

However, it was felt that provision of the toolkit alone would not be sufficient, and that use and efficacy of the toolkit would be enhanced through external face-to-face support and discussions with other IRSs:

*“I think to really truly embed something they [IRSs] need that hand holding…  having had X years’ experience of teaching, leadership, you need to do the [role] modelling, you need to do the demonstration” (Participant 4)*

*“Sharing good practice is always a good thing…. If anybody wants to come and see how it works… and then you know, kind of learn from each other (IRS focus group participant)*

#### The intervention delivery context

Interviewees’ narratives indicated a shared view that involvement in health promotion is novel for most IRSs, but that it is a positive step forward given their access to communities:

*“I think it's an amazing initiative, especially because the kids spend a lot of time [at the IRS] after school. If we can do our part in the community and, you know, prevent childhood obesity as much as possible, yeah, why not? Let's go for it” (IRS focus group participant)*

Interviewees felt that the inclusion of Islamic narrative was key to engaging IRS in delivery and in the receptivity of families:

*“They [madrasa] have a very specific curriculum, specific aim and purpose. In order for us to be able to introduce healthy diet and healthy exercise into a religious setting we need to convince the setting that they're teaching it anyway… that is why Islamic narrative is so important for the madrasas” (Participant 12)*

*“You can tell them about all these healthy recipes and stuff but when you bring it from an Islamic point of view, it just means so much more….  just mentioning something like that it's the Sunnah, they'll just take it so much more seriously” (Participant 2)*

However, participants voiced likely delivery challenges, such as the novelty of the agenda for IRSs, the volunteer nature of many IRS roles, and societal and funder perceptions of IRSs:

*“We can't take some of the models that are currently being used in schools because IRS are not at that level yet. So, they need additional resource to kind of get them started… and in the IRS’s they are often volunteers, unpaid staff who are giving their time to do something, perhaps don't have the capacity at the moment to be able to deliver” (Participant 10)*

*“I think sometimes just being an Islamic Religious Setting might put you on the backend. Because people might think… ‘what are they going to do?’... which I think is quite unfair… if you're putting in this hard work, you don't want it to be impacted just because of the title that ’oh, we're not gonna give you funding’” (IRS focus group participant) *

There was a perception that IRS’ opportunity to attract funding to deliver health promotion activities, which may be impacted by Islamophobia, could limit longer-term sustainability of toolkit intervention delivery.

### Theme two: the power of co-production

This these describes how, through involving multiple stakeholders with different perspectives and expertise, and taking a collaborative approach, there was benefit to the product (i.e. the toolkit) and the longer-term sustainability and impact of the work.

#### Enhancing the product (toolkit) through co-production

Stakeholders had different but each highly valuable roles that they were able to fulfil, based on their background, experience, and expertise:

*“As a collective [we] have all the skills and expertise that are needed… I felt it was a really strong team…. it allowed me to feel confident in my own abilities, because of the support of the other people… we've got people with behaviour change [expertise], with experience teaching, with experience in Mosque and madrasa settings, with a healthy diet background…. they can complement each other, and you can draw strengths from different ways of doing things” (Participant 7)*

The collective contribution of, and interaction between, multiple stakeholders meant that stakeholders felt more comfortable in their role, knowing that they didn’t have to do or know everything, and this was felt to increase the quality of the output. The value of the input of those with personal and/or professional experience of the IRS context was noted most frequently:

*“The main thing is the difference that I've made as a Muslim… what you may find in other programs is that things are delivered just based on somebody's experience [who is not] even from the culture or from the background” (Participant 11)*

Muslim stakeholders spoke of how their inside knowledge of cultural norms and beliefs facilitated the development of content that was acceptable amongst Muslim communities. These interviewee narratives pointed to perceptions or previous experiences of interventions being developed by academics or subject experts and that whilst these interventions may be scientifically sound, they are not culturally appropriate or feasible for delivery within IRSs, which restricts intervention effectiveness.

Nevertheless, certain roles (community engagement) and stakeholder groups (local government, public health) appeared instrumental to achieving *wider* impact:

*“[they, i.e. CEM] pulled everything together, [they’ve] obviously got lots of knowledge, lots of links, and obviously was very encouraging… [they] got us all organised and obviously that produced results… for me having that element was really good” (IRS focus group participant)*

*“[I’m] trying to embed the Trailblazer [toolkit] work as part of the wider [public health] activity that's going on so that it's all linked together with some level of synergy…. my role really is to make sure that the work that's happening is embedded into everyday practice and to how we tackle obesity in Bradford” (Participant 15)*

Having a dedicated CEM facilitated capacity building for health promotion amongst IRS’s and partnering with the local government enabled integration of the obesity-prevention toolkit within the district public health strategy and delivery.

#### Building capacity and ownership for delivering health interventions through IRSs

Interviewees described how early involvement and engagement of IRS stakeholders facilitated ongoing partnerships to deliver health promotion activity:

*“Co-production I guess helps people recognize why they're doing it and gives them that sense of ownership” (Participant 15)*

*“Islamically… It's called a Shūrā* 1 *… an Islamic way of thinking where you get people together and talk about it… because the Prophet (PBUH) used to do that with his companions… for people to be involved in the decision makes them feel important” (Participant 2)*

Stakeholders recognised that sharing decision-making power and input at the stage of intervention development helped build commitment to addressing childhood obesity amongst IRS stakeholders. The co-productive approach is widely used within Islamic settings, and taking this approach to working with IRSs for supporting the health agenda is likely to be consistent with the values and behaviours of IRS staff. IRS stakeholders spoke about how, since their involvement in developing the obesity-prevention toolkit, they had initiated implementation of health-promotion activities within their madrasa setting:

*“We've done the healthy eating and the snacks and the hydrating. The girls have tried out a few of the sports activities. Boys are always on the sports activity. We're in the process of opening up [venue] across the road, where we will have better facilities to go and do some of these activities”* *(IRS Focus group participant)*

### Theme three: negotiating power, involvement, and perspectives within co-production

This theme focuses on the challenges and nuances of doing co-production within the context of developing the obesity-prevention toolkit and emphasises issues of power and inclusion with regards to varying levels of control and influence stakeholders could assert throughout the process.

#### Varying and restricted stakeholder involvement and power

A range of factors influenced stakeholders’ level of involvement and influence in the co-production process. Some stakeholders were specifically employed to work on the toolkit development, whereas others were fitting it in around their primary roles or participating in a voluntary capacity, which limited time and energy available to engage in the process:

*“There were people's names assigned to do certain parts of the work… [but] in reality I don't think that people have really been given the true capacity within their day jobs… so it was a job to do, but no time to do it in” (Participant 14)*

*“People were dropping out of meeting saying, ‘I'm really sorry, I've got to go pick the kids up, I've got to go shopping’. And there were times when it came to my bit of the meeting, I only had two people, so I couldn't do the session” (Participant 1)*

There was a perception that IRS stakeholders’ *opportunity* to shape the toolkit was limited compared to stakeholders within the toolkit development group:

*“I think it would have been good to have IRS representatives as part of the actual development group… because when you've already written something and they're basically providing feedback, it's not going to drastically change it” (Participant 7)*

Place-based group members were not offered the opportunity to work as part of the toolkit development group, which indicated an inequity in power.

#### Community engagement

The CEMs engagement approach was fundamental to empowering IRS stakeholders to participate in co-producing the toolkit. Key to this was informing, including, and inspiring:

*“The more informed they [IRS stakeholders] are the more aware and involved they could be… I want to make sure that they understand that this is co-produced… it's not something that will be done to them it’s going to be with them… we want to inspire this community to say that this is our business as well… you have to make them understand they are equal partners, and experts in their own right, to address childhood obesity” (Participant 1)*

*“They [CEM] were like the glue, essentially, so they were very good and prompt with their reminders… They were motivating and encouraging” (IRS focus group participant)*

It was deemed important to ‘go to people where they are’ when engaging IRSs:

*“We have gone out to them, to their organization… to engage them, rather than calling them into a central place where they are uncomfortable” (Participant 12)*

As well as the physical meeting location, interviewee narratives suggested the importance of understanding the context, agenda and priorities of IRSs and factoring this into interactions, rather than focusing solely on the obesity-prevention toolkit agenda:

*“This is a lesson to other agencies… sometimes they don’t understand the community... but because [CEM]s a Muslim… [they] were sensitive to… there are lots of politics within Mosques, there are different roles… so [they] listened to all those people” (IRS focus group participant)*

Having a Muslim CEM who understood IRSs helped build trust amongst IRS stakeholders. The data indicates that adaptability facilitated the ongoing engagement of IRS stakeholders:

*“Our school starts at four and finishes at seven. So, most of our meetings took place at half eight to half nine [PM]… they [CEM] have done that with enthusiasm... they understand how many of us are very, very busy and they make allowances for that in terms of making sure they can accommodate and work within those parameters” (IRS focus group participant)*

One interviewee highlighted that it was important to engage gatekeepers to establish and maintain engagement of IRSs in the obesity-prevention toolkit development process:

*“Things work in a bit of social hierarchy, which is partly religious, partly cultural… [we] had to maintain very good relationships with five, six key individuals who are significantly important in Bradford, [who] lead community on all various issues. We need their blessing for us to be able to engage with IRS. That was going on in the background” (Participant 8)*

#### Negotiating different perspectives

The data highlighted difficulties of simply incorporating and consolidating divergent perspectives into the toolkit development process and output:

*“Some of the toolkit’s development meetings were very heavy on talking because everyone was coming at it from a different perspective. And I think there was always an idea of wanting to get consensus on any idea before it goes forward, but I think it was difficult to sort of understand what needs to happen off the back of that” (Participant 14)*

*“[In] the initial stages it was very protracted… what I learned was co-production is sometimes to test your patience” (Participant 12)*

The data highlighted that negotiating different perspectives was time-consuming and that it was difficult to incorporate all viewpoints and satisfy all stakeholders. For example, stakeholders expressed differing views regarding whether evidence or local knowledge should have driven the toolkit development, and whilst some felt there was value in subject expert stakeholders being involved in co-producing the toolkit even if they lacked an understanding of the IRS context, others felt that an understanding of the IRS context should have been a pre-requisite to participation:

*“Initially [name] was given the commission… they don’t understand the [IRS] context… we don't want to deliver services that are not appropriate to the people that are willing to, you know, receive those services (Participant 11)*

Stakeholders also disagreed about the importance of utilising behaviour change principles within the toolkit development process:

*“From my perspective, taking a behaviour change approach is about the process of developing the toolkit… I felt as though some of the people in the team thought behaviour change was a product… it was said to me a few times like, ‘Oh, you just add in the behaviour change’… and I think at that point, I thought, just take a step back” (Participant 6)*

A perception amongst some stakeholders that their role or input was not valued amongst the wider teams sometimes contributed to disengagement with the process.

### Theme four: complexities of effective communication within co-production

Interviewees felt that effective communication was a persistent challenge throughout the project, which led to a lack of role clarity amongst stakeholders and made it difficult to embed consistency and connectivity across the different toolkit components.

#### Communication channels and processes

Stakeholders described inadequate formal communication channels within smaller working groups (e.g., physical activity), between the smaller working groups, and with leadership:

*“We got put into these small groups, but from a physical activity perspective, I don't think that worked very well, because it didn't happen basically…. there wasn't any facilitation to help that happen…. there wasn't a clear communication structure, or there wasn't clear mechanisms and channels of communication” (Participant 7)*

Whilst many of the contributors worked collaboratively to produce toolkit content, IRS stakeholders were at more of an arms-length from the working group, as the process established by leadership was for place-based groups to communicate via the CEM, which various stakeholders felt was limiting:

*“If we'd have been going along to the testing [place-based groups] we would have seen how people reacted to it and that would really help improve the quality of our contribution as well. But that was just never made available as an option” (Participant 6)*

*“It would have been nice to be able to explain that visually and talk through things [with IRS stakeholders] and to demonstrate…  [it would be better to] have the meetings in the madrasa settings so that we can see the environment” (Participant 4)*

An open dialogue between toolkit development group members and IRS stakeholders may have improved the efficiency and output of the co-production process. However, stakeholders voiced that the process was directed by leadership, and that they lacked power and influence in making adaptations based on their expertise and learning. Another contextual factor that hindered communication processes was the complexity of the project:

*“There were lots of components in terms of management… When you have a complicated system like this, it's hard to understand who's leading it. Where is the direction coming from? It wasn't an easy process… There was no head. Other people might see it differently. But that's how I [see it], which makes decision making difficult” (Participant 11)*

The toolkit development took place during the height of the COVID-19 pandemic which made effective communication more difficult:

*“The third lockdown was where things crumbled… it was meant to be a face-to-face community engagement activity, it was never meant to be an online co-production methodology… although everybody's saying, ‘oh wow you've achieved that’, trust me I've got a lot of grey hairs that came out of that process” (Participant 1)*

#### Clarity, consistency, and connectivity

Communication challenges contributed to various unintended negative consequences, including a lack of role clarity amongst co-production stakeholders:

*“I'm coming in as a [topic] specialist… But it wasn't entirely clear, practically, what does that actually involve?... ‘how much time am I meant to be giving to this?... Are there any objectives for me to achieve?’ and I didn't really get an answer to that” (Participant 9)*

A lack of clarity or misunderstandings of roles caused inefficiencies and hindered some stakeholders’ contribution to the toolkit development, for example one IRS stakeholder believed they had been involved from a copy-editing perspective:

*“Being a teacher, my main input was correcting all the spelling mistakes and grammar mistakes, syntax errors that were in the text. Because that's, I mean, that's just something that I need to make… to make sure that things are right” (IRS focus group participant)*

This IRS stakeholder misunderstood their role which meant that they did not contribute to the toolkit in the way that was originally intended, i.e. providing feedback, changes, and additions based on their experience as a Muslim and IRS stakeholder. To improve role clarity, stakeholders suggested that a project induction, including documentation outlining all stakeholders’ roles, should be provided.

A lack of communication between stakeholders working on different toolkit components (physical activity, healthy diet, organisational change), hindered potential connectivity and consistency between different sections of the toolkit:

*“Reducing your carbon footprint, I mean that could have tied in with the being less sedentary and walking to the IRS. Yeah, madrasas organised walking busses, again, there's so many close ties, and delivers healthy eating messages, so it should all just been a lot more joined…. we were all very siloed or certainly, I felt very siloed” (Participant 6)*

Structured communication processes *between* topic groups, such as scheduled meetings facilitated by the project lead, and a template for session plans to be used by stakeholders producing content, may have supported connectivity and coherence across the toolkit.

## Discussion

This study examined the process of developing a health intervention with IRSs. It identified key benefits and challenges of co-production which advance knowledge production related to doing co-production in the context of health promotion broadly, and specifically when working with IRSs. This paper adds to the emerging evidence-base in support of delivering health promotion through IRSs e.g., [9, 28].

The findings illustrate that taking a toolkit approach and delivery through IRS were viewed overall as acceptable. The toolkit approach, whereby IRS can select from a menu of activities, reduced the burden placed on IRS stakeholders. According to the TFA, which outlines seven component constructs that underpin acceptability, an intervention is more acceptable if less perceived effort is required to participate [34]. Incorporation of Islamic narrative into the toolkit increased ethicality of the intervention, as it aligns health promotion with deliverers and users’ value system [34]. Interviewees highlighted that mutual discussion and decision making (co-production) is a fundamental Islamic principle (“Shūrā”). Compared to individual decision-making, it is considered to lead to greater understanding, co-operation, unity and social empowerment [21]. Thus, *co-producing* interventions may be particularly important for enhancing engagement and acceptability with Muslim communities with respect to subsequent intervention delivery, as it is likely to align with individual and community values.

The present study identified various benefits of co-producing an obesity-prevention toolkit. One such benefit was that it facilitated IRS engagement with the health promotion agenda through building ownership amongst stakeholders. Increased ownership through contributing to co-production has also been reported in other contexts, such as the co-design of an integrated model of care for people with COPD in rural Nepal [42]. However, the mechanism(s) through which involvement can contribute to capacity building and ownership amongst delivery stakeholders is largely unexplored in the literature. Findings from the present study suggest that participation in co-production of the intervention enhanced knowledge and understanding of the health problem as it required cognitive engagement, which contributed to building ownership. A second benefit of co-producing the intervention was that it enhanced the relevancy of the toolkit content. Yadav et al., [42] reported that co-production permits the development of contextually relevant evidence, which is supported by our findings that involving stakeholders with lived experience of the delivery context was key to enhancing feasibility and acceptability. A third benefit of co-producing the intervention was that it included a diverse range of stakeholders with differing experience and expertise. Co-production permits marginalised voices to be heard within research and intervention development processes and has the potential to mitigate against structural issues such as gender bias. This was particularly important in developing the obesity-prevention toolkit, and notably, over 60% of the IRS place-based group members were Muslim women, who are seldom heard in the context of the obesity-prevention agenda. Including a diverse range of stakeholders also fostered reciprocity, i.e. exchange of information for mutual benefit [20].

Our findings indicate that community engagement is a resource intensive, but necessary investment for meaningful co-production as it supported capacity building amongst IRS stakeholders. A recent paper outlined a framework for developing public health interventions which recommends building capacity with communities to support engagement in co-production [43]. We found that the CEMs personal characteristics influenced whether and how IRSs engaged in the co-production process, those with personal and professional experience of the IRS context are more able to utilise contextual knowledge to develop capacity to engage in co-production amongst IRS stakeholders. This suggests that it is important that people with lived experience of the target population or setting are part of the core facilitation team, not just engaged with as part of the co-production process, to maximise opportunity for meaningful co-production through IRS capacity building.

A key limitation of the co-production approach was that power was not shared equally amongst all stakeholders. The set-up of the co-production process meant that IRS stakeholders had limited opportunity for direct interaction with the toolkit development group, which restricted potential for exchange of skills and knowledge and limited equitable power sharing. A recent strategy, co-produced with community stakeholders in Bradford and Tower Hamlets, UK, recommends power sharing as a key principle to maximise the value of co-production approaches, which requires an acknowledgement of the imbalanced starting positions of the various stakeholders involved in co-production [2]. The toolkit development group stakeholders also felt that the co-production *process* was largely controlled by leadership and that the opportunity (power) to shape the process would have improved both their experience, and the toolkit product. Adopted such an approach to co-designing a collective leadership educational intervention for health-care teams as stakeholders’ feedback shaped subsequent co-production workshops [25]. The researchers found this relinquishment of power challenging but recognised the value of this for ensuring a genuine co-design partnership.

The present study highlighted a range of practical challenges that can arise when co-producing interventions. One such challenge was negotiating different perspectives, including different views related to the importance of academic evidence and theory, within the co-production process. A recent commentary argued that co-production may compromise the scientific endeavour as decisions may be made that are not based on the best available evidence [23]. However, a response to this commentary highlighted that this argument assumes that uncovering ‘truth’ is the goal of research, co-production may be best aligned with a constructivist framework that recognises how people (including researchers) influence research and co-production processes [41]. Despite this, on a practical level, co-production inevitably involves differences of opinion amongst stakeholders due to diverse backgrounds, experiences and perspectives. Therefore, we believe that it is unhelpful to assume that taking a co-productive approach is a magic pill that will facilitate the development of an intervention that works for everyone, variation in contextual factors such as IRSs operating models and theological positions will lead to differences in toolkit acceptability and uptake in different IRSs. A second challenge was that taking a co-productive approach *added* complexities to communication processes in the present study, leading to inconsistency and lack of connectivity between different toolkit components. This led to delays in finalising the obesity-prevention toolkit. A standardised template for toolkit sessions would facilitate consistency, and cross-component communication channels would facilitate connectivity across different stakeholder groups. A recent systematic review found that communication was identified as an enabler and/or barrier to research co-production in 72% of included case studies (*n* = 109) [10]. It is important to acknowledge the practical challenges and inevitable diversity of thought involved in co-producing interventions, to develop practical strategies to overcome such issues.

After this study was completed work to finalise and implement the toolkit continued. Two workshops with stakeholders involved in health promotion programmes across Bradford District were conducted to gain wider feedback on the toolkit and inform revisions to ensure the toolkit content was consolidated across the different components and compliant with Public Health England's health promotion guidelines. The obesity-prevention toolkit is currently being implemented across IRSs in Bradford, UK. A purpose-built community development organisation, Faith in Communities, employs community engagement staff to support and train IRSs to implement select toolkit activities (based on their preferences/local context) through the Active Faith Settings programme (funded by the Bradford Local Delivery Pilot, Sport England) and the Living Well Faith Settings programme (funded by Bradford Council). There is potential to adapt the toolkit to other geographical contexts, and for evaluating effectiveness of the toolkit intervention for preventing obesity in British Muslim families.

### Strengths and limitations

This study adds to the small but growing literature evaluating co-production approaches to intervention development e.g. [16, 20, 25, 42]. It is important to examine the ‘who, when, what, why, and where’ of co-production to inform a nuanced understanding of how co-production works in different contexts [37], and this study is the first to examine such a process in an IRS context. The present study was conducted during the COVID-19 pandemic, which may have influenced the co-production process as it primarily took place online. However, hybrid and online methods are increasingly being used and so this does not necessarily limit the relevance of the study. A further limitation of the co-production approach is that it took place within a specific geographic context (Bradford, UK), where the majority of Muslim’s attending madrasa are from South Asian backgrounds (primarily Pakistani). It is unclear to what extent cultural values and norms specific to Bradford South Asian communities have informed the toolkit content and, as such, whether the toolkit is transferable to IRSs that serve different Muslim communities or whether adaptation would be required. Finally, we did not explore participants’ prior knowledge and experience of co-productive working in this study, which may have shaped their reflections on the co-production process and the feasibility and acceptability of co-producing the toolkit. This is an important consideration for future research.

## Conclusion

This study examined the feasibility and acceptability of co-producing an obesity-prevention toolkit for IRSs. Four themes were crafted from the qualitative data focused on attitudes towards obesity-prevention through IRSs, the benefits of co-production, negotiating involvement, power and different perspectives, and the complexities of effective communication within co-production. Co-production was beneficial for building capacity for delivering health interventions within IRSs, and for ensuring the toolkit was contextually appropriate. However, employing co-production facilitators that have lived experience of the IRS context was necessary to maximise such benefit.

### Supplementary Information

Additional file 1: COREQ checklist.

Additional file 2: Toolkit.

Additional file 3: TIDieR checklist.

Additional file 4: Interview topic guide.

## Data Availability

The dataset that will be generated and analysed during the current study are available from the corresponding author on reasonable request.

## Abbreviations

CEM: Community Engagement Manager
IRS: Islamic Religious Setting
TFA: Theoretical Framework of Acceptability

## Footnotes

1 Shura (Arabic: شُورَىٰ, romanised: *shūrā*, lit. 'consultation'). The Quran encourages Muslims to decide their affairs in consultation with each other.
