# Correction: Reflections on co-producing an obesity-prevention toolkit for Islamic Religious settings: a qualitative process evaluation

**DOI:** 10.1186/s12966-024-01630-6

**Published:** 2024-07-18

**Authors:** Jennifer Hall, Rukhsana Rashid, Abida Rafiq, Kiran Fatima, Sally E. Barber, Sufyan Abid Dogra

**Affiliations:** 1 Bradford Institute for Health Research, Bradford Teaching Hospitals NHS Foundation Trust, Bradford Royal Infirmary, Duckworth Lane, Bradford, BD9 6RJ UK; 2 Faculties of Life Sciences and Health Studies, University of Bradford, Richmond Road, Bradford, BD7 1DP UK; 3 Core20Plus5, Craven Health and Care Partnership, NHS West Yorkshire Integrated Care Board, Scorex House, Bradford District &, Bradford, BD1 4AS UK; 4 Faculty of Management, Law and Social Sciences, University of Bradford, Richmond Road, Bradford, BD7 1DP UK

**Correction to: Hall et al. Int J Behav Nutr Phys Act (2024) 21:63**

10.1186/s12966-024-01610-w.

Following the publication of the Original Article, the authors identified an error in a name mentioned in the Acknowledgements section.

**Incorrect:**

Rachael Hill

**Correct:**

Rachael Moss

The Original Article has been corrected.

