# Supplementary material for: Reflections on co-producing an obesity-prevention toolkit for Islamic Religious Settings: a qualitative process evaluation
Source: Int J Behav Nutr Phys Act. 2024 Jun 12;21:63. doi: 10.1186/s12966-024-01610-w (PMC11170851; doi:10.1186/s12966-024-01610-w)
Supplement: Supplementary file 1 — Additional file 1: COREQ checklist. [file 12966_2024_1610_MOESM1_ESM.docx]

**Trailblazer Toolkit Process Evaluation Topic Guide**

Topic 1 - The programme and your role

- What is your understanding of Trailblazer Childhood Obesity prevention programme/Toolkit?
- When and how did you become involved in Trailblazer programme/Toolkit?
- How would you describe your role/ involvement? - e.g. time commitment, type of thing you have been doing, involvement over time, others from your IRS?

Topic 2 - How have you found being involved?

- What do you think / feel about the programme? Do you like the idea of the toolkit?
- What have you liked about being involved?
- What have you not liked about the process?
- Is there anything that made it difficult for you to contribute? Any challenges?
- Is there anything that made it easier for you to contribute?

(Think about communication processes, working with community engagement managers, covid-19 etc.)

Topic 3 - The impact of your work

- Do you feel like you were able to shape the content of the toolkit? What is the value of this? What has the impact of your involvement been on the toolkit?
- Has been involved in the project had any impact on you personally or your IRS, or more broadly? In what way?

Topic four - reflection and learning

- What would you personally, or what would you recommend the trailblazer team do differently, if developing the toolkit again?
- Did you have sufficient time for reflection? What specific tools, processes or approaches helped you in capturing your learning?
- What are the next steps for you regarding this work? What do you think of the toolkit, will you use it within your IRS?
- Is there anything else you wanted to say?
