# Supplementary material for: Reflections on co-producing an obesity-prevention toolkit for Islamic Religious Settings: a qualitative process evaluation
Source: Int J Behav Nutr Phys Act. 2024 Jun 12;21:63. doi: 10.1186/s12966-024-01610-w (PMC11170851; doi:10.1186/s12966-024-01610-w)
Supplement: Supplementary file 3 — Additional file 3: TIDieR checklist. [file 12966_2024_1610_MOESM3_ESM.docx]

**The TIDieR (Template for Intervention Description and Replication) Checklist*:**

We have completed the TiDieR checklist for the Living Well and Active Faith Settings intervention, for which the obesity prevention toolkit is a central component. Our paper presents a qualitative study examining the process of co-producing the toolkit specifically, prior to the full development and delivery of the wider intervention. As such, some items within the checklist were not relevant or appropriate to report within this paper.

| **Item number** | **Item** | **Where located **** | |
| --- | --- | --- | --- |
|  |  | Primary paper  (page or appendix  number) | Other ^†^ (details) |
|  | **BRIEF NAME** |  |  |
| **1.** | Provide the name or a phrase that describes the intervention. | ____7_____ | ______________ |
|  | **WHY** |  |  |
| **2.** | Describe any rationale, theory, or goal of the elements essential to the intervention. | ____7______ | _____________ |
|  | **WHAT** |  |  |
| **3.** | Materials: Describe any physical or informational materials used in the intervention, including those provided to participants or used in intervention delivery or in training of intervention providers. Provide information on where the materials can be accessed (e.g. online appendix, URL). | ____7______ | Link to toolkit |
| **4.** | Procedures: Describe each of the procedures, activities, and/or processes used in the intervention, including any enabling or support activities. | ___7+26____ | _____________ |
|  | **WHO PROVIDED** |  |  |
| **5.** | For each category of intervention provider (e.g. psychologist, nursing assistant), describe their expertise, background and any specific training given. | ___7____ | [Home - Faith in Communities](https://faithincommunities.co.uk/) |
|  | **HOW** |  |  |
| **6.** | Describe the modes of delivery (e.g. face-to-face or by some other mechanism, such as internet or telephone) of the intervention and whether it was provided individually or in a group. | ____7____ | _____________ |
|  | **WHERE** |  |  |
| **7.** | Describe the type(s) of location(s) where the intervention occurred, including any necessary infrastructure or relevant features. | _____7______ | _____________ |
|  | **WHEN and HOW MUCH** |  |  |
| **8.** | Describe the number of times the intervention was delivered and over what period of time including the number of sessions, their schedule, and their duration, intensity or dose. | _____n/a____ | _____________ |
|  | **TAILORING** |  |  |
| **9.** | If the intervention was planned to be personalised, titrated or adapted, then describe what, why, when, and how. | _____26_____ | _____________ |
|  | **MODIFICATIONS** |  |  |
| **10.^ǂ^** | If the intervention was modified during the course of the study, describe the changes (what, why, when, and how). | _____n/a___ | _____________ |
|  | **HOW WELL** |  |  |
| **11.** | Planned: If intervention adherence or fidelity was assessed, describe how and by whom, and if any strategies were used to maintain or improve fidelity, describe them. | ____n/a______ | _____________ |
| **12.^ǂ^** | Actual: If intervention adherence or fidelity was assessed, describe the extent to which the intervention was delivered as planned. | ____n/a_____ | _____________ |
