# Supplementary material for: Reflections on co-producing an obesity-prevention toolkit for Islamic Religious Settings: a qualitative process evaluation
Source: Int J Behav Nutr Phys Act. 2024 Jun 12;21:63. doi: 10.1186/s12966-024-01610-w (PMC11170851; doi:10.1186/s12966-024-01610-w)
Supplement: Supplementary file 4 — Additional file 4: Interview topic guide. [file 12966_2024_1610_MOESM4_ESM.pdf]

LIVING WELL FAITH SETTINGS

A TOOLKIT FOR PROMOTING HEALTHY BEHAVIOURS THROUGH MADRASAS AND OTHER ISLAMIC FAITH SETTINGS

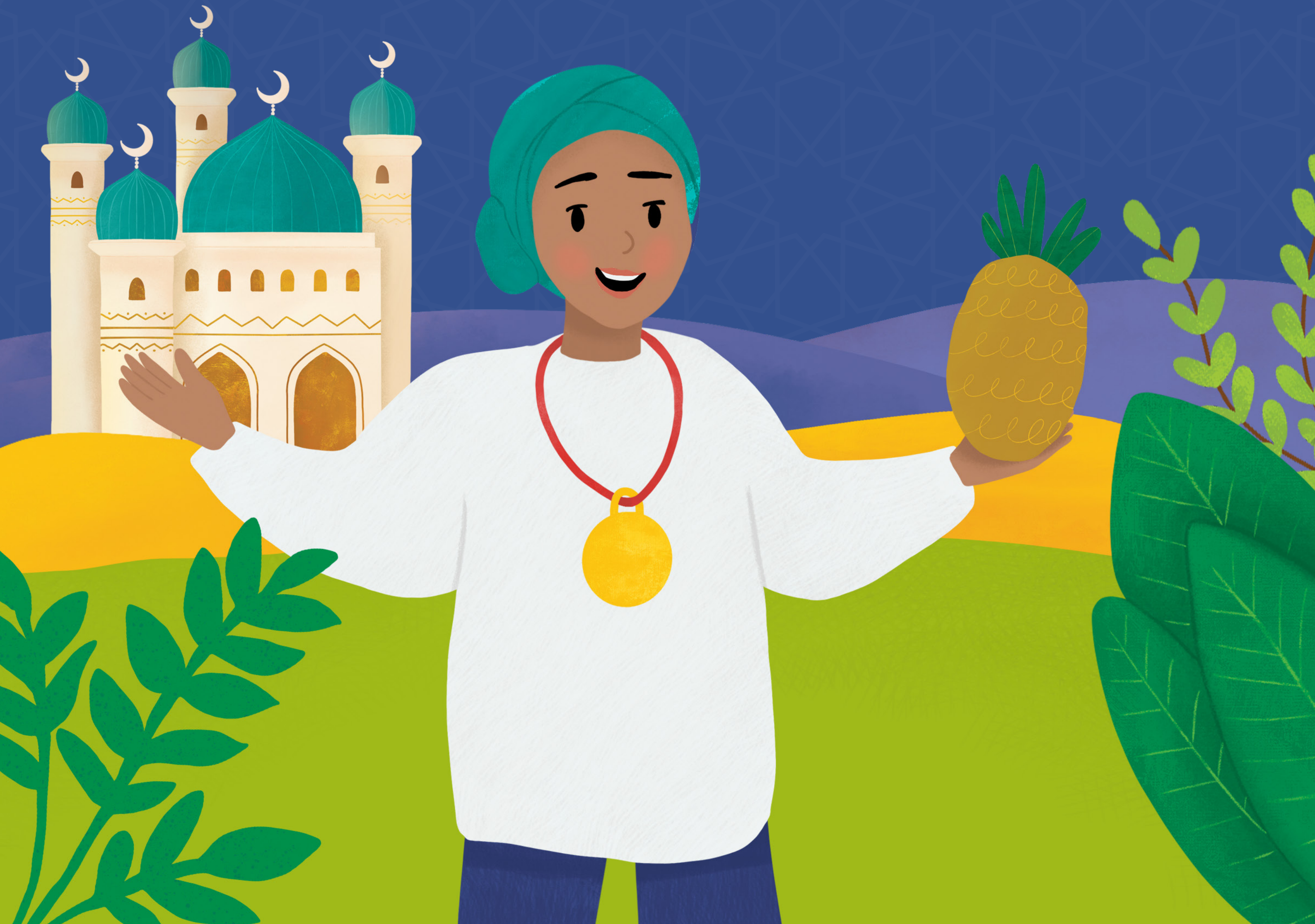

OVERVIEW

This Living Well Faith Settings toolkit has been funded by the **Local Government Association** and **the Department of Health and Social Care** Childhood Obesity Trailblazer Programme (2019-2022). The grant was awarded to **the City of Bradford Metropolitan Borough Council** in 2019 to be delivered in partnership with **Born in Bradford, the Bradford Institute for Health Research**, and **Faith in Communities**; a local community organisation focussed on health promotion in faith settings.

The design, dissemination, local mobilisation and testing of this toolkit has been funded by **Living Well**; the district's whole systems approach to obesity and wellbeing jointly led by **the City of Bradford Metropolitan Borough Council** and **the Bradford District and Craven Health and Care Partnership**, and **JUMP, Bradford's Local Delivery Pilot funded by Sport England.**

The reference, commentary and interpretation of the Islamic narrative has been developed and added by the British Islamic theologian and scholar Mufti Mohammed Zubair Butt. The inclusion of the religious narrative within this toolkit does not constitute endorsement by partner organisations or the LGA and DHSC as funders. All enquiries relating to the Islamic narrative should be referred to Mufti Mohammed Zubair Butt directly at *zubair.butt@faithincommunities.co.uk.*

INTELLECTUAL PROPERTY RIGHTS

This toolkit was developed by Born in Bradford, the City of Bradford Metropolitan District Council, and Faith in Communities through funding from the Local Government Association and the Department for Health and is subject to ©Crown copyright and available under the Open Government Licence v3.0.

We request that, should you intend to use this toolkit for any kind of commercial or research funding application purposes, you inform the Director of Public Health, City of Bradford Metropolitan District Council at *Sarah.Muckle@bradford.gov.uk* and *borninbradford@bthft.nhs.uk.*

PARTNERS

Born in Bradford

JU:MP – Bradford's Local Delivery Pilot

Faith in Communities

Living Well

Department of Health and Wellbeing, Bradford Council

Department of Health and Social Care

Bradford Institute for Health Research

Sport England

Local Government Association

ACKNOWLEDGMENTS

Many individuals contributed on various components during the development and co-production of this toolkit. This *Living Well Faith Settings* toolkit is a result of voluntary contributions by community members, leaders and staff in madrasas, parents of children attending madrasas, and local community organisations collaboratively working among diverse teams of researchers and practitioners. This toolkit is a true example of how co-production works and how evidence-based health policies can be translated into a programme of action in culturally sensitive ways to improve health outcomes in ethnic minority populations in the UK.

AUTHORS

Dr Sufyan Abid Dogra, PhD, Communities Involvement and Implementation Lead, Born in Bradford, Bradford Institute for Health Research, led the co-production of the toolkit and coordinated the collaborative work of diverse teams, providing input on this toolkit over five years (2018-2023). He undertook the rigorous and pioneering research and data collection, analysed and published data, collated evidence, secured grants and funding, co-designed the implementation model, and led the co-production of all components and sections of this toolkit on encouraging and implementing healthy behaviours using madrasas and other Islamic faith settings. He ensured that the scientific rigor, public health messages, and community insights and priorities are truly reflected in the contents of this toolkit by continuously building trust and ownership with Islamic faith settings, scholars, and leaders to stay involved in the co-production of the toolkit. He disseminated the process of co-producing the toolkit to national and international audiences over 5 years.

Dr Sally Barber, PhD, Principal Research Fellow, Born in Bradford, Bradford Institute for Health Research, conceived the study, analysed data on health inequalities in ethnic minorities, secured grants and funding, undertook the research, supervised the co-production process, supervised, supported, and mentored Dr Sufyan Dogra over five years, organised panels of national experts on shortlisting healthy behaviours, co-designed the implementation model, disseminated the findings of research behind this toolkit, and provided input to different research teams. Dr Sally Barber also provided technical and thematic input on physical activity workshops and aligned the co-production of this toolkit with wider research, evidence and implementation programmes.

Rose Dunlop, Deputy Director of Public Health, played an instrumental role working with Born in Bradford to secure the initial LGA Childhood Obesity Trailblazer Programme funding for Bradford Council. Rose has been fundamental in embedding the principles of cost-effectiveness and sustainability throughout the initial planning, developing the theory of change and logic model. By driving the focus on enabling settings to deliver this work for themselves, Rose has ensured the work was able to be transitioned into a sustained Public Health offer both now and into the future. In 2017 Rose initiated Living Well, the district's whole systems approach to obesity & wellbeing. *Living Well Faith Settings* is now one of the over 15 Living Well core projects which, alongside policy makers and the work programmes of other partners, are working together to make it easier for everyone in Bradford to live healthier, more active lives.

CONTRIBUTORS

Tim Howells, Head of Public Health, Healthy Communities, Bradford Council has led the project management, co-ordination, and quality and accuracy checks on this toolkit. Tim organised dedicated workshops with wider public health experts and system leaders to ensure community groups views and public health guidelines are in alignment. Tim Howells continues his support in the implementation of toolkit workshops through his leadership of Living Well Faith Settings and the Living Well Healthy Communities work area.

Mufti Mohammed Zubair Butt, Chair, Faith in Communities provided his expertise in the development and selection of the relevant Islamic narrative on health promotion, such as verses from the Quran and sayings of Prophet Mohammed (pbuh) as a supplementary message to increase the uptake of healthy behaviours in madrasas, and among parents and children attending madrasas. He used his influence as an Islamic leader to bring diverse mosques, madrasas, and health champions from ethnic minorities on board and pushed madrasas to continue encouraging healthy behaviour as an ongoing activity.

Nicola Knowles and **Grainne Dickerson,** Senior Public Health Specialists from Bradford Council contributed in the project management and co-ordination from a public health perspective.

Nabeela Khan (healthy diet), **Mufti Mohammed Zubair Butt** (Islamic narrative and healthy places), **Bryn Llewellyn** and **Ashfaq Ahmed** (physical activity), **Dr Fiona Fylan** (behaviour change techniques), and **Shazia Rafiq** (organisational behaviour change), worked as subject matter experts and provided their input on the content development for toolkit workshops.

Dr Jennifer Hall, Senior Research Fellow, Born in Bradford, led the process evaluation on the co-production of this toolkit and disseminated it at an international forum in the UAE. She provided a behaviour change framework to underpin the toolkit development and recommended behaviour change techniques. Other research fellows from Born in Bradford who contributed to the development of the toolkit are **Rukhsana Rasheed**, who provided input on healthy diet content, **Dr Rosslyn Kerr** and **Dr Kate Lightfoot**, who both did a remarkable job in refining the contents of the toolkit workshops after repeated quality and accuracy sessions organised by Bradford Council in which colleagues from across the district provided input. **Dr Rosslyn Kerr** and **Dr Kate Lightfoot** also provided input in editing the overall presentation toolkit contents.

Faith in Communities as an organisation and several **Community Engagement Managers** performed a vital role of bringing madrasas onboard, ensured their involvement in the co-production of the toolkit, and presented the contents of toolkit workshops to health groups in madrasas (comprising of parents, staff, leaders, and volunteers in Islamic faith settings) to gain their input on what subject matter experts proposed. **Abida Rafiq** was instrumental in delivering test and learn sessions on various sports and healthy behaviours with health groups in madrasas. Her repeated interactions with health groups were very useful in gaining insights from members of the communities. **Nasiba Siddig** discussed the finalised contents of behaviour change workshops with health groups affiliated with madrasas and got input from female leaders on the acceptability of toolkit contents. She provided her pictures to be included in the toolkit to facilitate the delivery of physical activity workshops. **Maryam Malick** and **Abdullah Butt** supported the implementation of the toolkit workshops before these were designed. Both received the feedback of health groups on how practical it is to plan and implement toolkit workshops in madrasas.

Professor Rosie McEachan, Director Born in Bradford played a key role in securing the grant funding from LGA by working closely with Public Health colleagues in Bradford Council. She provided her

input on the theory of change and logic model for the programme. Rosie contributed to community consultations for the programme design and brought system leaders on board. Many thanks to the overall support and guidance of **Professor John Wright**, Director Bradford Institute for Health Research, and **Sarah Muckle**, Director Public Health, Bradford Council.

We express our gratitude to the exceptional team at **Knowledge to Action (Reyhana Ismail, Waqaus Ali and Zahra Patel)**, whose project management and creative and diligent efforts were instrumental in elevating the toolkits to a professional standard and ensuring they have been designed to be culturally appropriate for our intended audience.

We are humbled by the selfless and voluntary contributions of Islamic faith settings in Bradford. We are extremely thankful to children in madrasas, parents of children attending madrasas, Imams, Islamic leaders, management, teachers and staff in mosques and madrasas who contributed proactively to the co-production of this toolkit. Special thanks to madrasas in Bradford including Al Mustafa Centre, Masjid Umar, Islam Bradford, Bayt al Qaim, Islamic Tarbiyya Centre, Abu Hurairah Academy, al-Markaz al-Islami, Al Hikam, Masjid Bilal, Masjid Hussain, and many other mosques and madrasas in Bradford who participated in test and learn sessions on the co-production of the contents of this toolkit. Without your help, the task of co-producing this toolkit couldn’t have been accomplished.

WELCOME

It gives me great pleasure to present the Living Well Faith Settings toolkit, a remarkable resource to be used by madrasas and other Islamic faith settings in the United Kingdom, to encourage healthy behaviours among children, parents, staff, management and leaders. This toolkit has been co-produced to support madrasas and other Islamic faith settings to encourage healthy behaviours, such as having a healthy diet and being physically active, with the aim of making it easier for children, young people and families to live healthier and more active lives.

In my mind, I have never questioned whether Islamic faith settings can be agents for change in health promotion. This toolkit has simply provided evidence that mosques and madrasas in Bradford have been an untapped resource for too long in the promotion of the health and wellbeing of children and families in deprived neighbourhoods. Thousands of children and families from ethnic minorities will benefit from encouraging and implementing healthy behaviours in madrasas and other faith settings and in the community. This toolkit empowers staff in madrasas to be the change themselves and contribute to the health and wellbeing of children attending faith settings. This toolkit confirms that long lasting behaviour change for health and wellbeing in deprived neighbourhoods is possible if local authorities and community organisations involve health groups in madrasas by sharing learning and resources with them and work directly with them.

An Islamic narrative (verses from the Holy Quran, sayings of the Prophet Mohammed (pbuh) and appropriate historical accounts) is used throughout the workshops to supplement and reinforce healthy behaviours. The Islamic narrative is included in the toolkit for the purpose of enabling madrasas and Islamic faith settings to engage with establishing health promoting practices. I developed and added the references, commentary, and interpretation of the Islamic narrative in this toolkit.

Finally, I wish you well on your journey to improving the health and wellbeing of the children, parents, staff, management, and civil and faith leaders of the future.

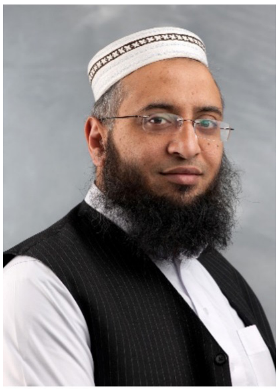

Mufti Mohammed Zubair Butt
Chair, Faith in Communities
Jusrisconsult, Hospital Chaplain and Islamic Leader
zubair.butt@faithincommunities.co.uk

RECOMMENDATION FOR USERS

As a madrasa, it is recommended that you start by undertaking Healthy Places Workshop 1 which will support your setting to establish a 'health group'. This group will help you to create lasting changes for your faith setting. The group will hold the responsibility for deciding upon and organising/timetabling all further workshops. They will also take responsibility for general promotion of healthy behaviours across the faith setting. This group should consist of faith setting leaders, management and central decision makers for the faith setting, mosque or madrasa – whether these are staff, volunteers, teachers or parents of children attending madrasa: any group involved in decision making can be part of this group.

Once your health group is established, together you can look through the rest of the workshops in this toolkit, discuss which ones would be appropriate to run in your setting and make an action plan. The remaining workshops set out in this toolkit can be used by your health group to support the delivery and promotion of healthy behaviours in your setting. The workshops on preventing childhood obesity cover the following aspects:

1. **Healthy places** to support your setting to adopt health promotion across all aspects of the faith setting

2. **Healthy diet** for all members of the family

3. **Physical activity** both inside the faith setting (e.g. use of active breaks within curriculum time) and outside (e.g. by working with local sports or youth clubs).

The contents of each workshop are flexible and can be adapted to the needs and interests of those taking part. The toolkit has been developed with a broad audience in mind and is intended for use with children of all ages. For some workshops, the activities and discussion points may be more or less relevant for children of different ages. It is intended that workshop facilitators adapt elements of the activities and discussions to be appropriate for the ages of the children they are working with.

Your health group should choose the workshops to deliver that are of interest to the group and however many can be delivered with the number of staff/volunteers you have to support this.

The facilitator for the delivery of these workshops can be an externally funded community engagement manager/worker, a local subject matter expert (e.g. a nutritionist, for the healthy diet workshops) or a local health practitioner. Alternatively, faith settings can identify and choose their own facilitator for the delivery of the workshops if there is internal expertise (e.g. volunteers with relevant knowledge). An externally funded community engagement manager can work with the health group affiliated with the faith setting to deliver and implement toolkit workshops or they can co-ordinate between the health group and a local subject matter expert. An externally funded community engagement manager can also facilitate the training of staff from the faith setting, or a person designated by the health group, on how to deliver these workshops themselves. There is additional information at the end of this toolkit which includes a list of useful resources to support the delivery of this toolkit.

This toolkit is coproduced by Born in Bradford, Bradford Council, and Faith in Communities. For details, see introduction.

LIVING WELL FAITH SETTINGS

CONTENTS

Healthy Places 9

1 Organising Healthy Faith Settings 11
2 Accurate Health Information 21
3 Encouraging Healthy Lifestyles 28
4 Connecting Faith Settings and External Agencies 34
5 Transport, Travel, and Managing Vehicles 39
6 Safe Walking and Cycling 45
7 Working Together and Building Relationships 50
8 Collaborations and Applying for Small Grants 56

Healthy Diet 61

1 Introduction to Nutrition 63
2 Creative Kitchens 68
3 Drinking Water 75
4 Mealtimes and Manners 85
5 Portion Size 93
6 Shopping and Food Labels 99
7 Healthy Snacking 105

Physical Activity 110

1 Reducing Sedentary Time and Increasing Physical Activity 112
2 Physical Activity in the Curriculum 121
3 Engaging Parents through Fun Days and Sports 128
4 Extracurricular and Leisure Time 134
5 Being Active through Travel 143
6 Using Islamic Narrative to Support Physical Activity 149

Further Information and Resources for Facilitators 155

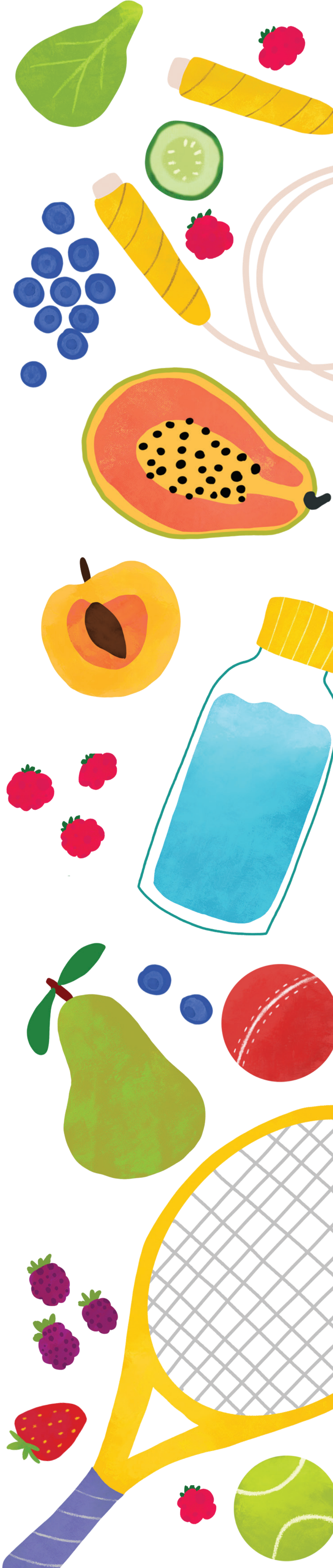

HEALTHY PLACES

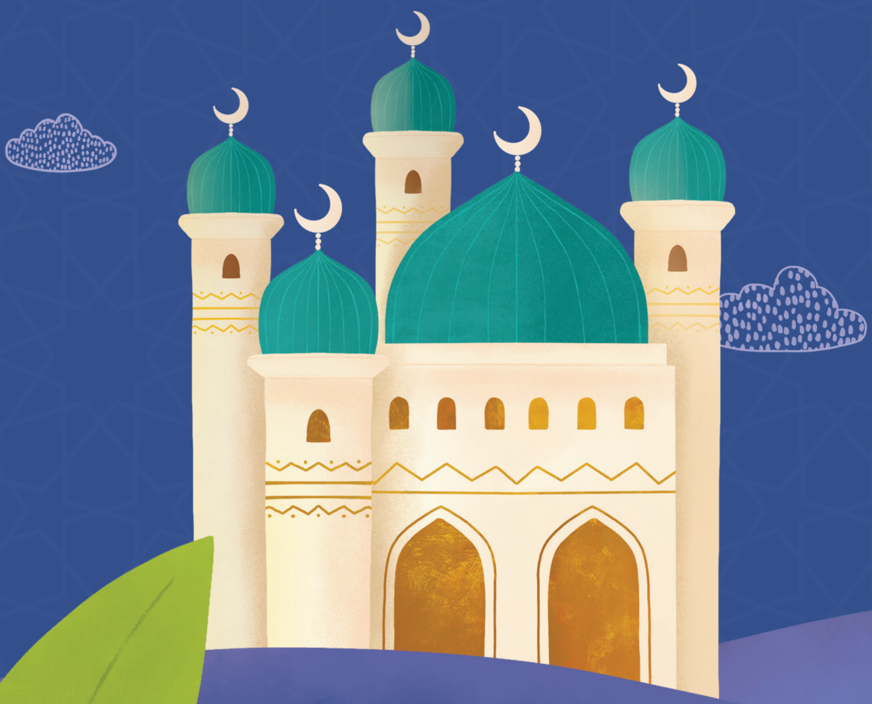

HEALTHY PLACES

The focus of this section is to understand the context and environment of the madrasas in order to harness their organisational potential for encouraging healthy behaviours and prevention of childhood obesity. The workshops in this section are largely intended to be delivered with the health group, to plan how healthy behaviours can be delivered within and across the faith setting. Workshops should involve parents and families where possible, to ensure full support for the plans of the health group and buy-in for the delivery of health behaviours for all involved. The aims of this section are:

AIMS:

- To provide guidance to decision makers and/or staff and volunteers on how to set up a system within the Islamic faith settings, to encourage healthy behaviours on a sustainable basis
- Train faith setting leaders, teachers and volunteers to have an institutional/structural approach to raise awareness, promote and facilitate health interventions within their setting for their community
- Faith setting leaders and teachers learn and recognise the faith setting's role as an influential and active partner in preventing childhood obesity

WORKSHOPS:

1. Organising Healthy Faith Settings
2. Accurate Health Information
3. Encouraging Healthy Lifestyles
4. Connecting Faith Settings and External Agencies
5. Transport, Travel, and Managing Vehicles
6. Safe Walking and Cycling
7. Working Together And Building Relationships
8. Collaborations and Applying for Small Grants

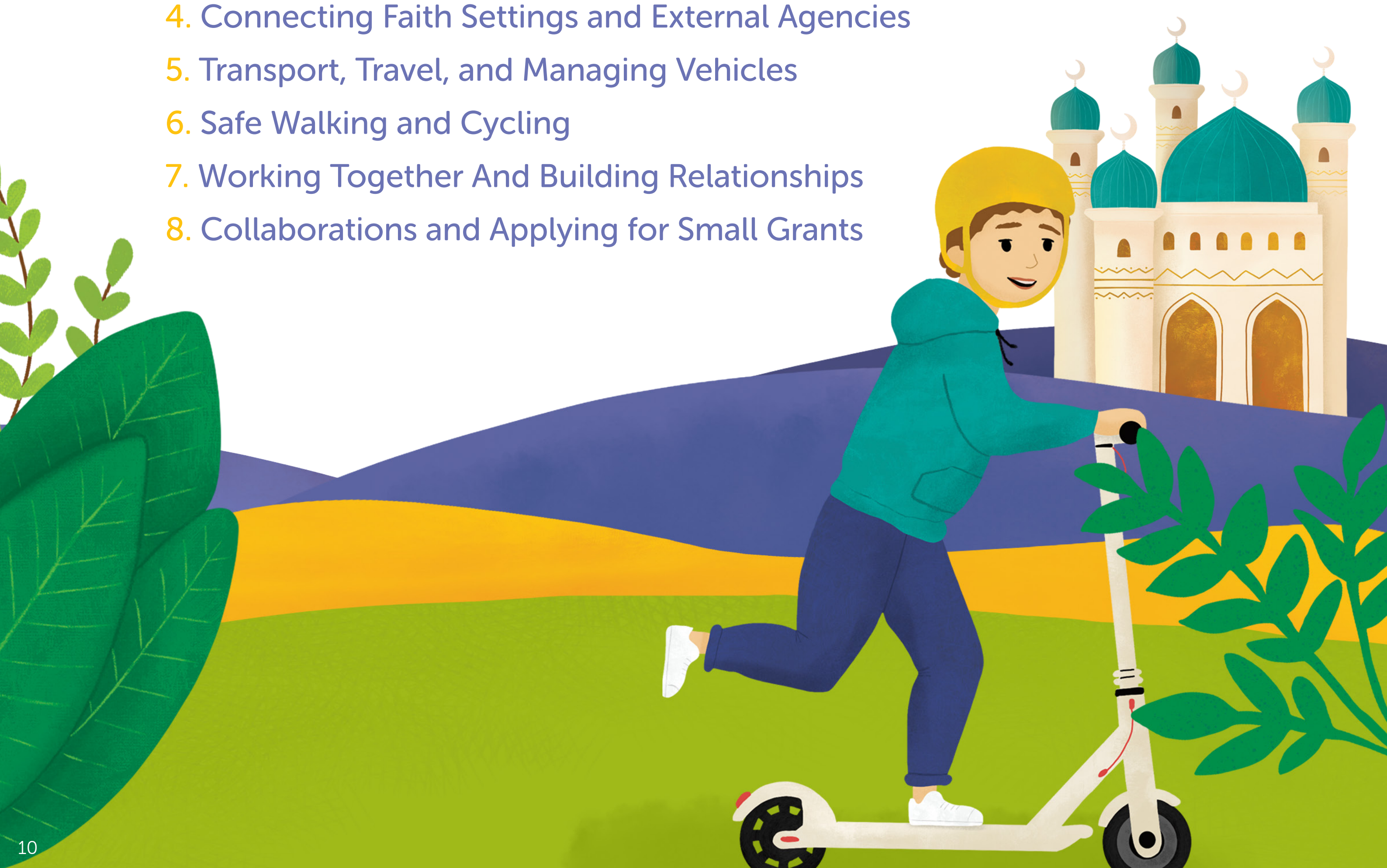

We are family BORNINBRADFORD | JU:MP | FAITH IN COMMUNITIES | LIVING WELL | City of BRADFORD METROPOLITAN DISTRICT COUNCIL

Department of Health & Social Care | BRADFORD INSTITUTE FOR HEALTH RESEARCH MAKING RESEARCH REAL | SPORT ENGLAND | Local Government Association

HEALTHY PLACES WORKSHOP 1

ORGANISING HEALTHY FAITH SETTINGS

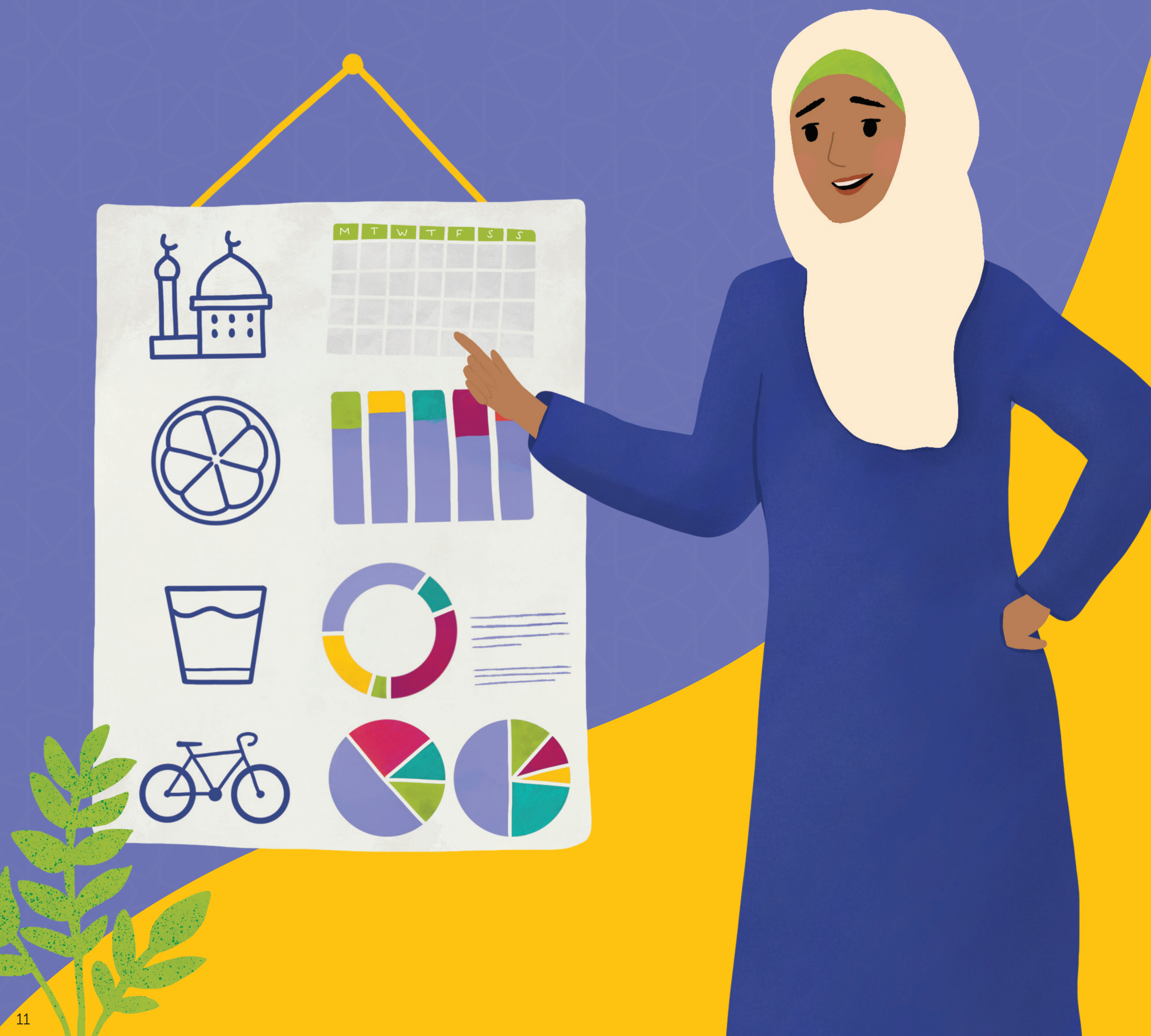

HEALTHY PLACES WORKSHOP 1

ORGANISING HEALTHY FAITH SETTINGS

AIMS:

- To establish a health group and an 'action plan' for subsequent workshops. Participants will understand the need for, and identify, actions for enabling healthy behaviours within the faith setting

OUTCOMES:

- The health group identifies and commits to at least one workshop that they can deliver, to make changes during madrasa time, and one workshop that they can deliver outside of madrasa time
- The faith setting has a sustainable plan for the future promotion of healthy behaviours (healthy diet and physical activity)
- The faith setting will understand and identify their own priorities and internal capacity of resources (skills and time available of staff and volunteers, as well as physical space) for health promotion

TARGET PARTICIPANTS:

- Management committee, senior leadership, staff, parents, volunteers, teachers and Imams
- Anyone who is important to decision making in the faith setting

LENGTH OF WORKSHOP:

- 1 hour

EQUIPMENT REQUIRED:

- Pens and paper for taking notes on planning/discussion
- Action plan template

LOCATION:

- Faith setting or any suitable space where participants can gather

HEALTH AND SAFETY:

- N/A

FOOTWEAR AND CLOTHING:

- N/A

ISLAMIC NARRATIVE

And help one another in righteousness and piety, and do not help one another in sin and aggression.
[Qur'an, 5:2]

And hold firmly to the rope of Allah all together and do not be divided.
[Qur'an, 3:103]

Whilst offering advice to a man, the Prophet Mohammed (pbuh) said, "Value five [things] before five [other things]: Your youth before your old age, your health before your illness, your affluence before your poverty, your availability before your occupation, and your life before your death.
[Al-Mustadrak]

[There are] two blessings in which many people are deceived: good health and free time.
[Al-Bukhari]

*City of Bradford Metropolitan District Council, and Born in Bradford do not own the Islamic Narrative in the toolkit and maintain this to be the independent work of Mufti Mohammed Zubair Butt. All enquiries relating to the Islamic Narrative should be referred to Mufti Mohammed Zubair Butt directly. For detailed disclaimer, please see page 2.

ACTIVITY 1
Discussion

10 mins

Why is it important to address unhealthy behaviours in the faith setting?

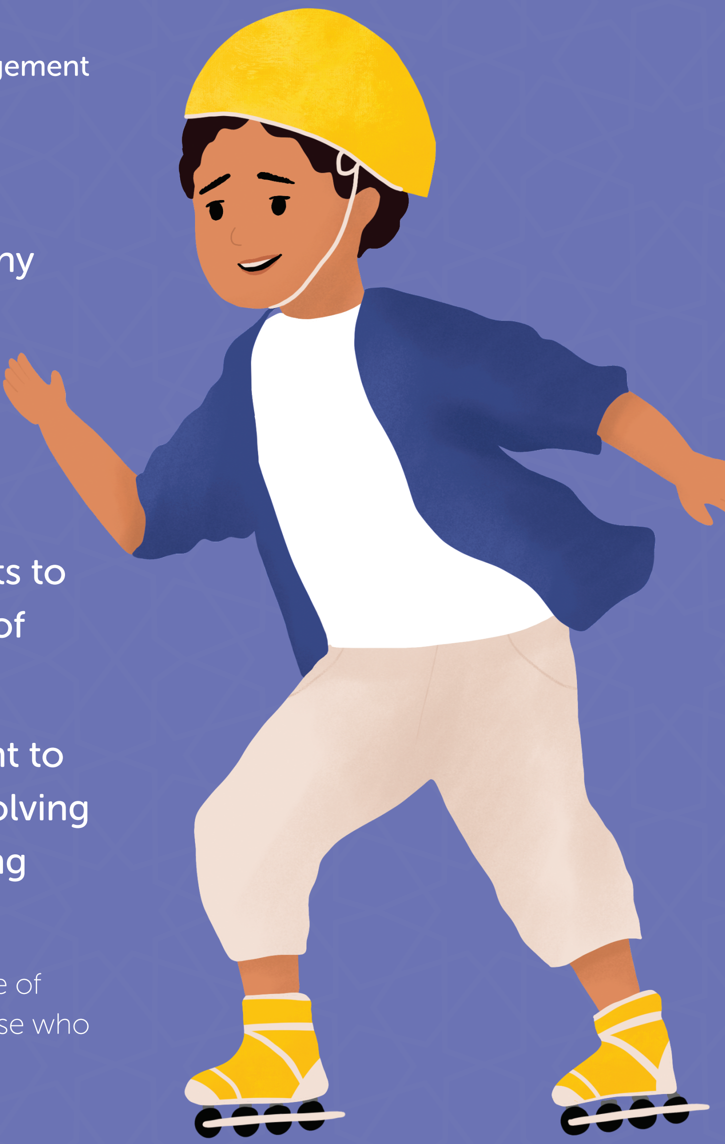

1. An Imam, Islamic leader or a community engagement manager can lead on this discussion.

   Points covered should include:

   - Why is physical activity and healthy diet intake important for children and families?
   - Describe childhood obesity and associated health risks etc.
   - Discuss the responsibility of adults to ensure the health and wellbeing of children in their care
   - Describe Islamic narrative relevant to maintaining healthy lifestyles involving physical activity and healthy eating

2. This discussion should end with identifying some of the key priority areas for the faith setting and those who agree to become members of the health group.

ACTIVITY 2
Action Plan

40 mins

The faith setting identifies their own healthy diet and physical activity priorities and produces an action plan.

A template of a plan is given below. One person should be nominated to fill in the plan whilst the group discusses.

Action:

1. Look through the workshops in this toolkit and think about how these can address the health priorities in your madrasa.. Consider the level of priority for each key area addressed by the workshops and detail the actions which will be implemented (i.e. who will take part in which toolkit workshops, giving exact details of children in which class, etc.).

2. Create a timetable as to when these could be delivered.

3. Think about what the specific areas of success look like, which will be appropriate for the resources that you have available (e.g. staffing) in completing each action. This could be the number of children or families taking part in particular workshops, or it could be the different behaviours that children may engage in (e.g. drinking more water or bringing in fruit as a snack during madrasa).

4. Use the toolkit workshops and other ideas to make your settings plan, and nominate someone to write the plan down whilst you are discussing. An example of a plan is given below.

? What actions can be taken to improve these? Settings should complete the table and prioritise each workshop and the relevance for the specific faith setting. '*What success looks like*' is also something which will be different and localised for the specific faith setting.

When making the plan, consider SMART goal making: the plan needs to be Specific, Measurable, Achievable, Relevant, and Time-bound.

AN EXAMPLE ACTION PLAN:

Key areas: Families to understand the importance of following the Eatwell guide

Priority: *E.g.* High

Actions: Children and parents take part in **Workshop 1 Introduction to Nutrition** from the Healthy Eating component

When: *E.g.* Within 1 month – 12/12/23

What success looks like: *E.g.* 25 families to take part in the workshop and gain an understanding of the different food groups

This toolkit is coproduced by Born in Bradford, Bradford Council, and Faith in Communities. For details, see introduction.

PRINT

ACTION PLAN:

Key areas: **Families to increase knowledge and skills around cooking healthy meals**

Priority: ....

Actions: Children and parents take part in **Workshop 2 Creative Kitchens** from the Healthy Eating component

When:..............................

What success looks like: ....

....

....

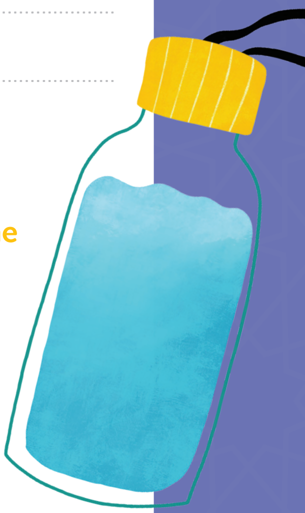

Key areas: **Children and families to drink more water and to understand the importance of doing so**

Priority: ....

Actions: Children and parents take part in **Workshop 3 Drinking Water** from the Healthy Eating component

When:..............................

What success looks like: ....

....

....

Key areas: **Children and families to understand the importance of family mealtimes**

Priority: ....

Actions: Children and parents take part in **Workshop 4 Mealtimes and Manners** from the Healthy Eating component

When:..............................

What success looks like: ....

....

....

This toolkit is coproduced by Born in Bradford, Bradford Council, and Faith in Communities. For details, see introduction.

Key areas: **Children and families to understand the need for appropriate portion sizes**

Priority: ....

Actions: Children and parents take part in **Workshop 5 Portion Sizes** from the Healthy Eating component

When:..

What success looks like: ....

....

....

Key areas: **Children and families to learn how to identify healthier options when shopping**

Priority: ....

Actions: Children and parents take part in **Workshop 6 Shopping and Food Labels** from the Healthy Eating component

When:..

What success looks like: ....

....

....

Key areas: **Children and families to identify healthier snack options**

Priority: ....

Actions: Children and parents take part in **Workshop 7 Healthy Snacking** from the Healthy Eating component

When:..

What success looks like: ....

....

....

This toolkit is coproduced by Born in Bradford, Bradford Council, and Faith in Communities. For details, see introduction.

PRINT

Key areas: **Faith setting leaders understand the importance of active learning**

Priority: ....

Actions: Faith leaders and stakeholders take part in **Workshop 2 Physical Activity in the Curriculum** from the Physical Activity component

When:..

What success looks like: ....

....

....

Key areas: **Mobilising parents to arrange and take part in fun days**

Priority: ....

Actions: Families and staff members take part in **Workshop 3 Engaging Parents Through Fun Days and Sports** from the Physical Activity component

When:..

What success looks like: ....

....

....

Key areas: **Children learn how to take part in various physical activities**

Priority: ....

Actions: Children take part in the activities detailed in **Workshop 4 Extracurricular and Leisure Time** from the Physical Activity component

What success looks like: ....

....

....

This toolkit is coproduced by Born in Bradford, Bradford Council, and Faith in Communities. For details, see introduction.

PRINT

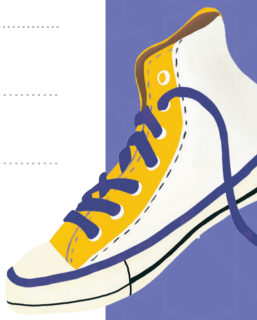

Key areas: **Families and staff understand the importance of active travel**

Priority: ....

Actions: Families and staff members take part in **Workshop 5 Being Active Through Travel** from the Physical Activity component

When: ....

What success looks like: ....

Key areas: **Families have more knowledge of Islamic narratives for physical activity for the community**

Priority: ....

Actions: Families take part in **Workshop 6 Using Islamic Narrative to Support Physical Activity** from the Physical Activity component

When: ....

What success looks like: ....

Any local health priorities not covered by the workshops?

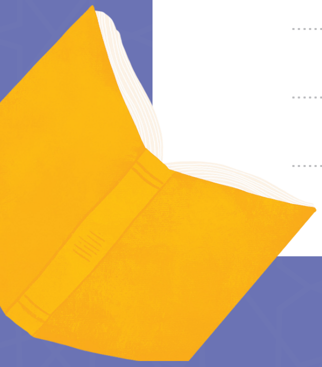

This toolkit is coproduced by Born in Bradford, Bradford Council, and Faith in Communities. For details, see introduction.

ACTIVITY 3
Discussion

10 mins

How can you mirror the Prophet's (pbuh) mosque and its functions in your mosque, in relation to health promotion and community development?

How can you sustain and continue healthy initiatives for the future?

With the priorities and action plan developed, the facilitator leads the discussion on the above points.

Action:

1. Appoint a health group to promote healthy behaviours among Islamic leaders, staff of the faith setting, management, children and parents - particularly girls and women.

2. Agree on how often the health group will meet to keep track of what progress is being achieved, and to record success of activities. Specifically agree the details on when the next meeting will take place.

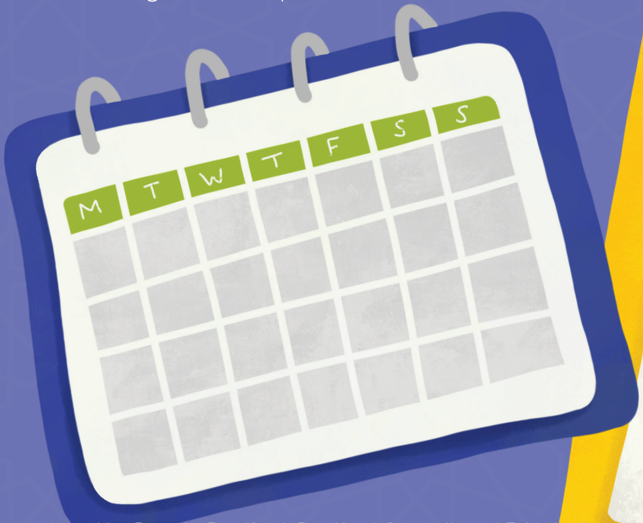

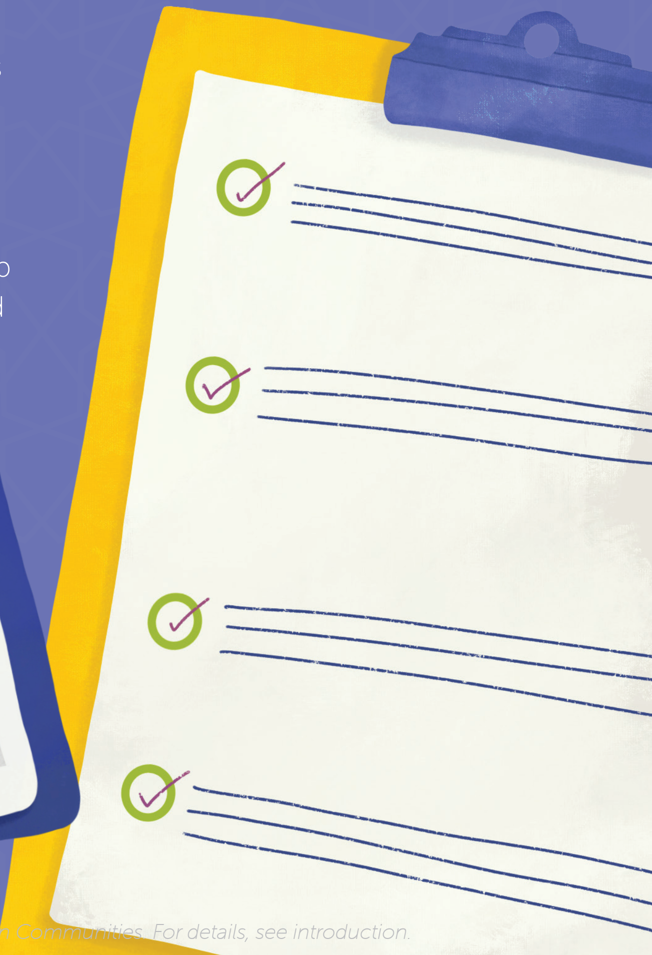

HEALTHY PLACES WORKSHOP 2

ACCURATE HEALTH INFORMATION

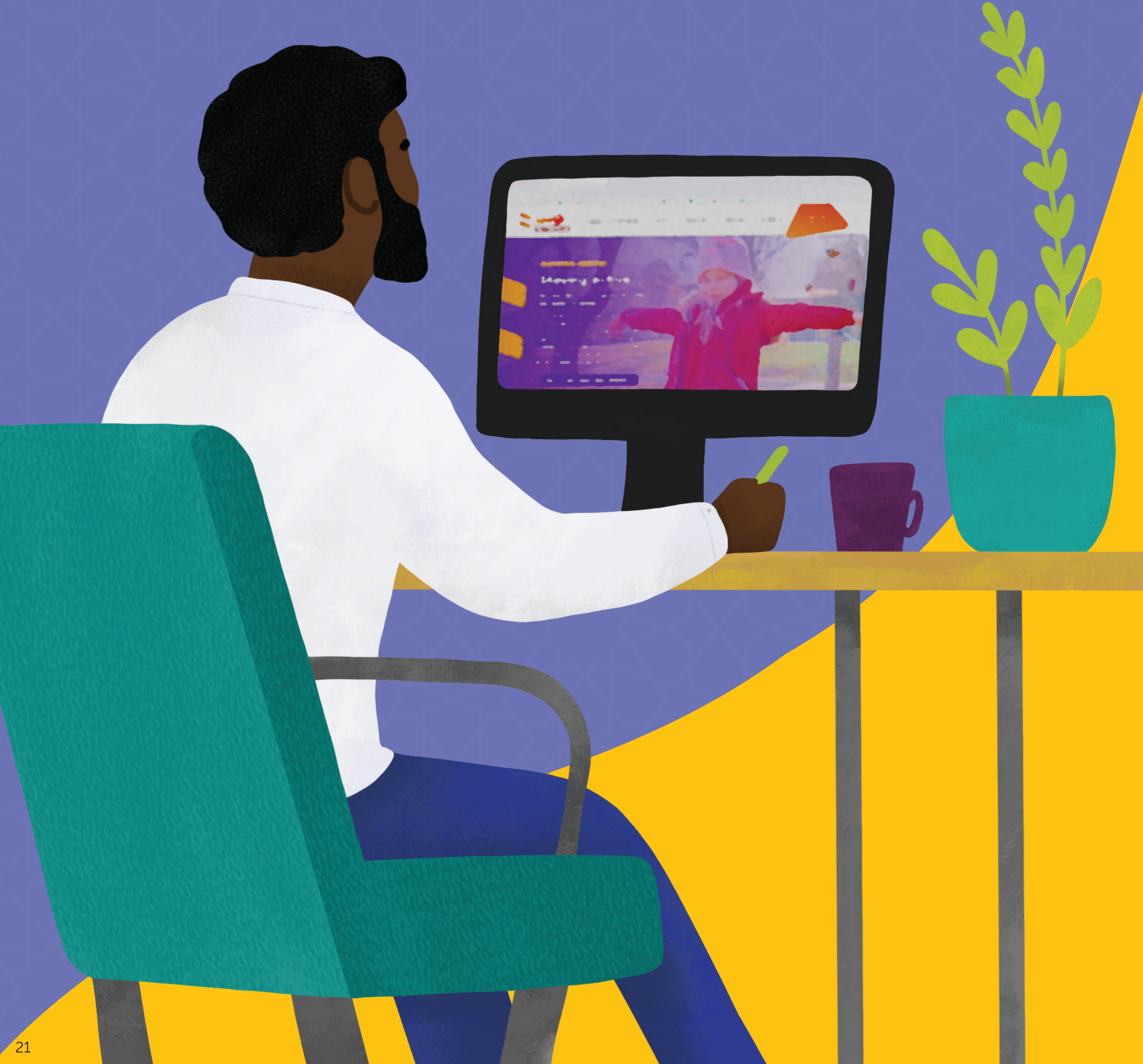

HEALTHY PLACES WORKSHOP 2

ACCURATE HEALTH INFORMATION

AIMS:

- To understand the importance of accurate health information
- To understand where accurate health information can be found

OUTCOMES:

- Faith setting staff and volunteers feel confident in identifying accurate health information
- Faith setting has a calendar of events throughout the year to deliver accurate health messages, and staff/volunteers have confidence to be able to share accurate health information with children and families

TARGET PARTICIPANTS:

- Imams, staff, teachers, volunteers, the health group

LENGTH OF WORKSHOP:

- 1 hour

EQUIPMENT REQUIRED:

- Pens and paper for taking notes on planning/discussion

LOCATION:

- Faith setting or any suitable space where participants can gather

HEALTH AND SAFETY:

- N/A

FOOTWEAR AND CLOTHING:

- N/A

This toolkit is coproduced by Born in Bradford, Bradford Council, and Faith in Communities. For details, see introduction.

And help one another in righteousness and piety, and do not help one another in sin and aggression.
[Qur'an, 5:2]

And hold firmly to the rope of Allah all together and do not be divided.
[Qur'an, 3:103]

Believers, when a corrupt person brings to you a piece of news, carefully ascertain its truth, lest you should hurt a people unwittingly and thereafter repent at what you did.
[Qur'an, 49:6]

Whilst offering advice to a man, the Prophet Mohammed (pbuh) said, "Value five [things] before five [other things]: Your youth before your old age, your health before your illness, your affluence before your poverty, your availability before your occupation, and your life before your death.
[Al-Mustadrak]

[There are] two blessings in which many people are deceived: good health and free time.
[Al-Bukhari]

*City of Bradford Metropolitan District Council, and Born in Bradford do not own the Islamic Narrative in the toolkit and maintain this to be the independent work of Mufti Mohammed Zubair Butt. All enquiries relating to the Islamic Narrative should be referred to Mufti Mohammed Zubair Butt directly. For detailed disclaimer, please see page 2.

ACTIVITY 1
Discussion

10 mins

This will be led by group members with expertise on Islamic guidelines and teachings (e.g. Imam).

Identify Islamic guidelines on health, and how these are sometimes not followed in relation to:

- Food
- Sleep
- Physical Health
- Mental Health (mindfulness)

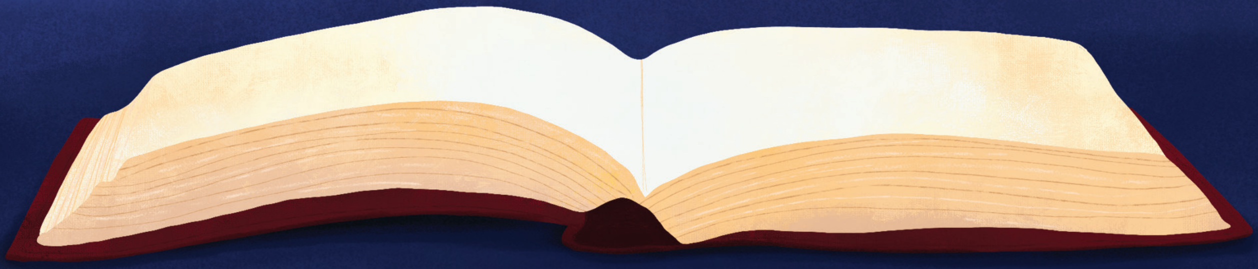

ACTIVITY 2
Discussion

10 mins

How to determine sources of accurate health information in our times and how can this be shared?

1. The following points can be used to shape the discussion:

- Why is it important to have accurate health information?
- Where and from whom can you get accurate health information?
- How and why should the spread of inaccurate health information be avoided? *(Quote Qur'anic verse 49:6 on spreading rumours and uncorroborated information; the group can consider the role of technology/internet in the spread of information)*

2 Facilitator then introduces roles and responsibilities, and shares details of the following organisations and how to access their services:

**Active Bradford**<br>activebradford.com	**Better Health**<br>nhs.uk/healthier-families	**For Bradford, Born in Bradford**<br>borninbradford.nhs.uk
**Islamic Relief**<br>bit.ly/42Y9mAi	**JU:MP**<br>joinusmoveplay.org	**My Living Well Programme**<br>mylivingwell.co.uk
**NHS – BD Care Trust**<br>bdct.nhs.uk	**NICE Guidelines**<br>bit.ly/3Me1Fix	**Office for Health Improvements and Disparaties**<br>bit.ly/3MDBEuE
**Public Health Bradford Council - Local Authority**<br>bit.ly/3ogekJV<br><br>**Public Health Early Years**<br>bit.ly/3Oke4Ek	**Faith in Communities**<br>faithincommunities.co.uk	• Community Health professionals, practitioners, experts and champions<br>• Community Health Voluntary sector.

ACTIVITY 3

Creating a Healthy Calendar

20 mins

Create an Islamic lunar calendar linking each month with a relevant health promotion idea (e.g. Ramadan and healthy foods when breaking the fast), and accurate health messaging.

It is important for the facilitator to note that the focus from each month should be continued, and that learning from one month is not forgotten.

Imams/Islamic leaders can help a lot here in identifying which health activity is more relevant for which Islamic month.

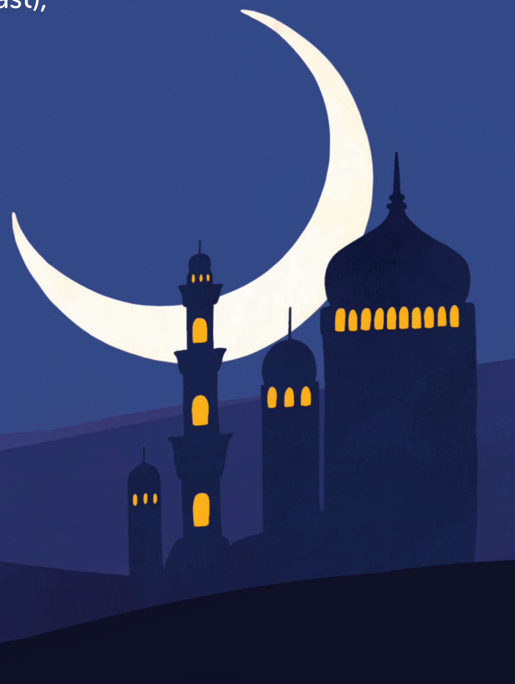

PRINT

1 Muharram

Significant Event: Islamic new year

Health Promotion Activity: Have new Islamic year resolutions to improve healthy eating, physical exercise and self-care.

2 Safar

Significant Event: ....

Health Promotion Activity: ....

....

3 Rabi-al-Awwal

Significant Event: ....

Health Promotion Activity: ....

....

4 Rabi al Thani

Significant Event: ....

Health Promotion Activity: ....

....

5 Jamad ul Awal

Significant Event: ....

Health Promotion Activity: ....

....

6 Jamad ul Thani

Significant Event: ....

Health Promotion Activity: ....

....

7 Rajab

Significant Event: ....

Health Promotion Activity: ....

....

8 Shaban

Significant Event: ....

Health Promotion Activity: ....

....

This toolkit is coproduced by Born in Bradford, Bradford Council, and Faith in Communities. For details, see introduction.

PRINT

9 Ramadan

Significant Event: ....

Health Promotion Activity: ....

....

10 Shawal

Significant Event: ....

Health Promotion Activity: ....

....

11 Dhul Qadah

Significant Event: ....

Health Promotion Activity: ....

....

12 Dhul Hijja

Significant Event: ....

Health Promotion Activity: ....

....

ACTIVITY 4
Friday Sermon

20 mins

Participants develop a plan for promoting health information before, during or after the Friday sermon (whenever a theme-based sermon is needed at any time during the year).

For example, from the calendar developed in Activity 3, build up a plan as to how each one can be delivered as part of a Friday sermon during the relevant months.

Some ideas in which this could be achieved are:

- **Using an Islamic narrative where needed to dispel myths** (e.g. narrative identified in Activity 1)
- **Inviting any health promoter to speak with the congregation** (e.g. one of those introduced during Activity 2)

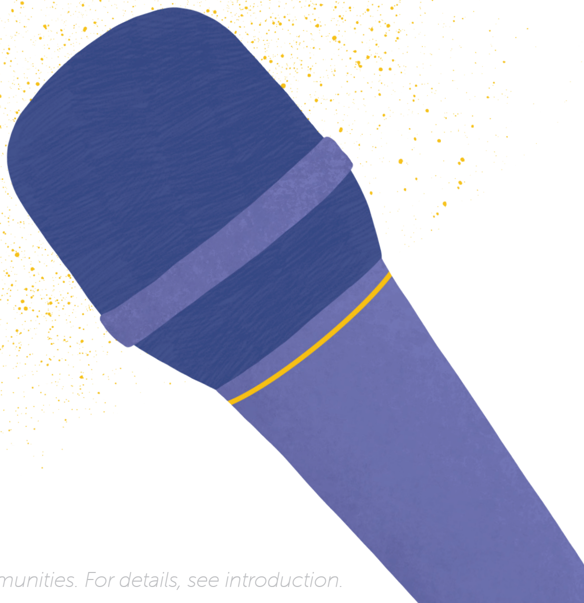

HEALTHY PLACES WORKSHOP 3

ENCOURAGING HEALTHY LIFESTYLES

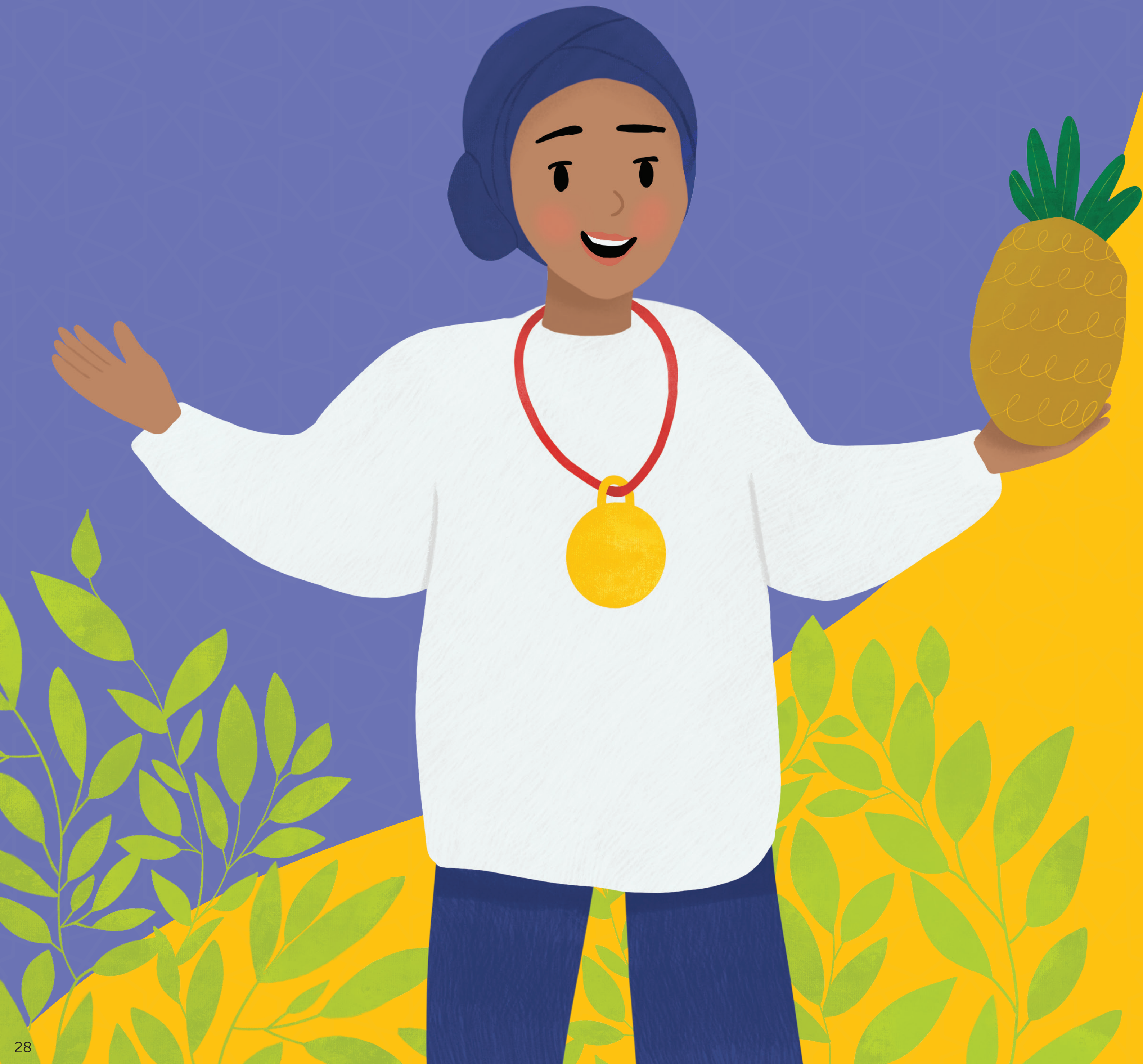

HEALTHY PLACES WORKSHOP 3

ENCOURAGING HEALTHY LIFESTYLES

AIMS:

- The faith setting community understand what behaviours can encourage healthy lifestyles (diet and physical activity)

OUTCOMES:

- The faith setting community feels empowered to form habits for behaviours which will encourage healthy lifestyles for the whole family
- The faith setting engages with local fast-food takeaway outlets to promote/introduce healthy food options for the community

TARGET PARTICIPANTS:

- Faith setting staff and volunteers, the health group

LENGTH OF WORKSHOP:

- 1 hour 10 minutes

EQUIPMENT REQUIRED:

- Pens and paper for taking notes on planning/discussion

LOCATION:

- Faith setting or any suitable space where participants can gather

HEALTH AND SAFETY:

- N/A

FOOTWEAR AND CLOTHING:

- N/A

This toolkit is coproduced by Born in Bradford, Bradford Council, and Faith in Communities. For details, see introduction.

And help one another in righteousness and piety, and do not help one another in sin and aggression.
[Qur'an, 5:2]

And do not throw [yourselves] with your [own] hands in to destruction.
[Qur'an, 2:195]

Whilst offering advice to a man, the Prophet Mohammed pbuh said, "Value five [things] before five [other things]: Your youth before your old age, your health before your illness, your affluence before your poverty, your availability before your occupation, and your life before your death.
[Al-Mustadrak]

*City of Bradford Metropolitan District Council, and Born in Bradford do not own the Islamic Narrative in the toolkit and maintain this to be the independent work of Mufti Mohammed Zubair Butt. All enquiries relating to the Islamic Narrative should be referred to Mufti Mohammed Zubair Butt directly. For detailed disclaimer, please see page 2.

ACTIVITY 1
Discussion

10 mins

What are the prevalences, causes and consequences of obesity?

Facilitator to lead discussion on the above question.

Points for discussion can include:

Prevalence:

- Local context (e.g. Born in Bradford research has shown that children from South Asian backgrounds have 10% higher rates of being overweight and obese than White British counterparts).
- Ethnic minorities (e.g. families often live in areas with high deprivation and high density of unhealthy food outlets).

Causes:

- Takeaways, sedentary behaviours, not enough physical activity, large portions etc.

Consequences:

- Risk to health (e.g. diabetes, heart disease, musculoskeletal problems, certain cancers).
- Economic (e.g. cost to the NHS in treating co-morbidities, cost to industry in days lost due to obesity related illnesses).

ACTIVITY 2
Friday Sermon

10 mins

Plan a focused Friday sermon or a speech to the congregation.

This can happen at any time of the year and encourages physical activity or healthy dietary behaviour to promote physical and mental health (the Islamic narrative at the beginning of each workshop in this toolkit which supports these behaviours can be drawn upon for content of the sermon).

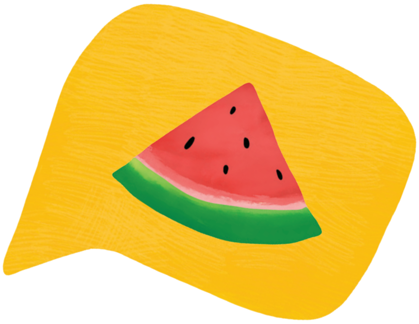

ACTIVITY 3

Health Message and a Challenge for Families

20 mins

The workshop participants should consider various health messages and challenges for families to complete, to encourage learning across the faith setting.

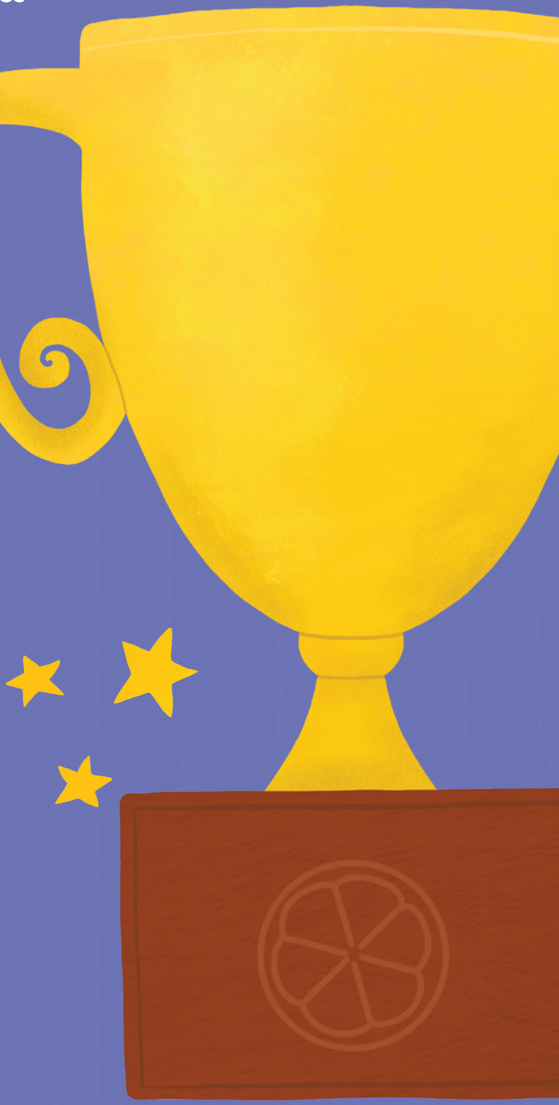

If appropriate, the faith setting could offer a prize (non-specific reward) for families who complete the most number of challenges over one or two months.

Facilitators should encourage participants to be creative in considering challenges, but remain realistic on what can be achieved with the available resources in the community.

An example challenge is given below.

Message:

Let's talk about healthy eating habits e.g. encourage children to eat with families and not separately.

Challenge:

(Take a voluntary pledge from participants)

- Share the message with their families
- Cook one meal together as a family, swapping unhealthy for healthy ingredients

Benefit:

Social, spiritual, physical, mental health.

Success:

(How did families respond to the challenge - well received, feelings, wellbeing etc.)

Good engagement with 10 families completing the challenge.

ACTIVITY 4

Improving Provision of Healthy Foods

30 mins

Part 1 (10 minutes)

Inside the faith setting:

The health group should discuss and agree on what foods should be offered during events held within the setting, and the types of food and drinks that are provided (e.g. 'pizza parties' and fizzy drinks for celebrations, or sweets to reward learning).

This toolkit is coproduced by Born in Bradford, Bradford Council, and Faith in Communities. For details, see introduction.

The group should agree to limits on provision of unhealthy foods used for rewards and celebrations and consider how healthy alternatives (e.g. fruit, water or fun active games) can be provided instead.

Part 2 (20 minutes)

Outside the faith setting:

Consider the provision of unhealthy food takeaway outlets close by.

Plan and then run a session to promote healthy diet for those involved in running these takeaway outlets.

Action:

1. Create a map of fast-food takeaway locations near to the faith setting that families often visit.
2. Identify those involved in managing these takeaways, and particularly if these people attend the mosque.
3. Discuss local takeaway provisions for children around faith settings, or invite people from local businesses and/or friendly takeaway owners to a toolkit workshop on healthy diet (if possible). Use the Islamic narrative to emphasise the importance of eating healthily and encourage participants to consider how they can help children to have healthy dietary options.

Make sure this is done without blaming, shaming or pointing fingers at anyone, and ensure that the discussion revolves around improving the health environment as a whole. The purpose of this activity is to engage with local businesses through discussion, by listening to their point of view on healthy diet provision, and not about asking or demanding them to change their takeaway menu.

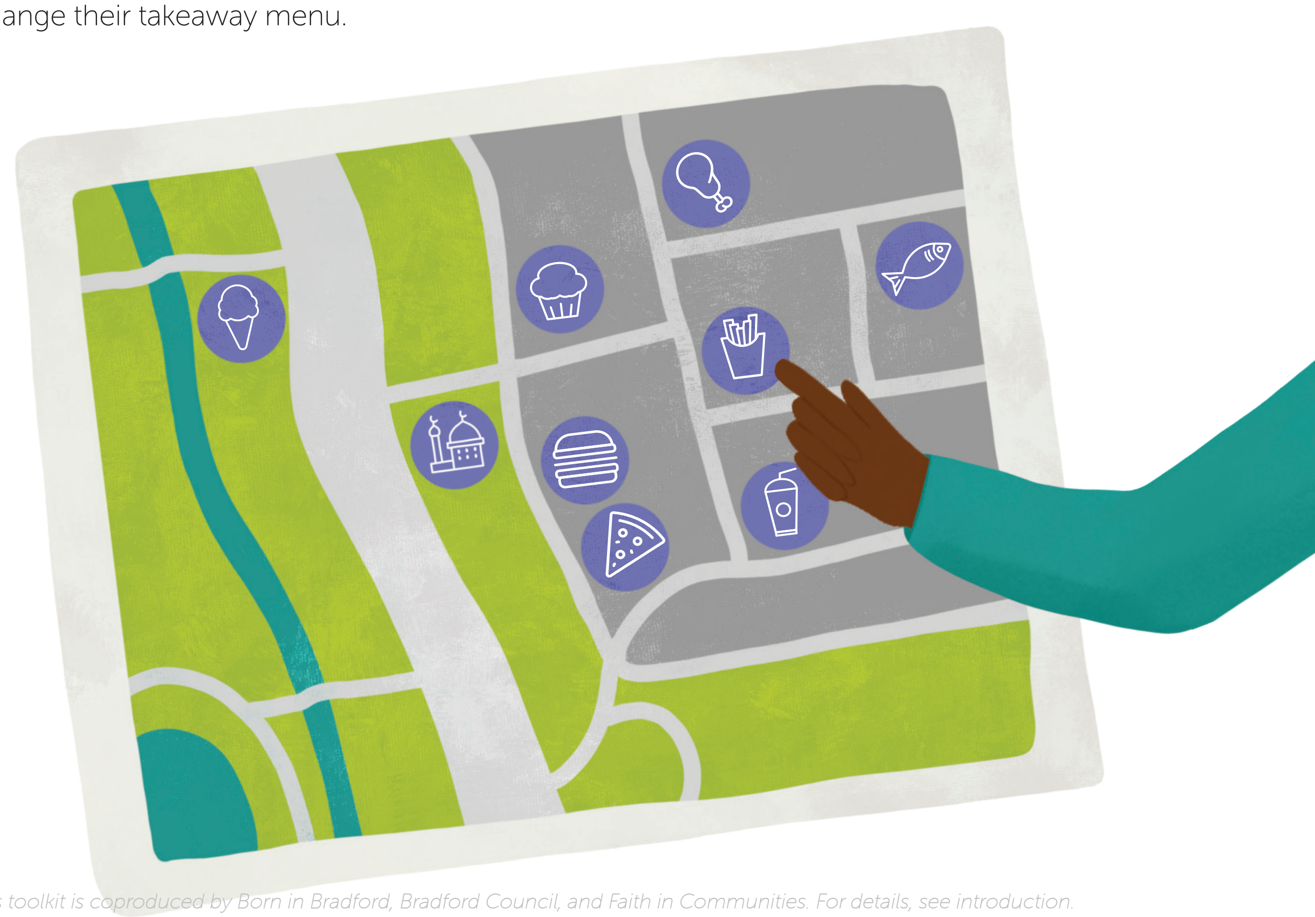

We are family BORNINBRADFORD
JU:MP
FAITH IN COMMUNITIES
LIVING WELL
City of BRADFORD METROPOLITAN DISTRICT COUNCIL
Department of Health & Social Care
BRADFORD INSTITUTE FOR HEALTH RESEARCH | MAKING RESEARCH REAL
SPORT ENGLAND
Local Government Association

HEALTHY PLACES WORKSHOP 4

CONNECTING FAITH SETTINGS AND EXTERNAL AGENCIES

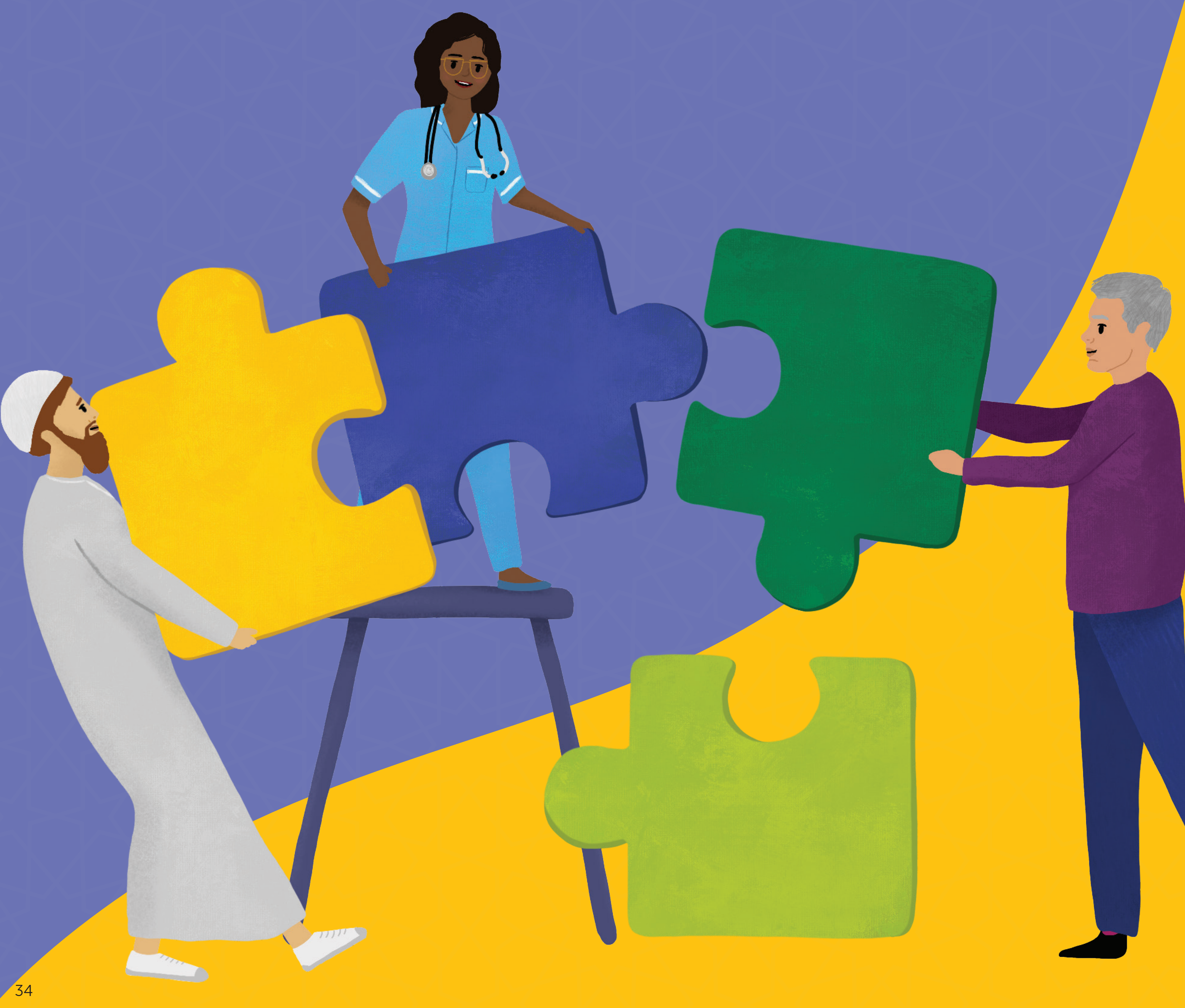

HEALTHY PLACES WORKSHOP 4

CONNECTING FAITH SETTINGS AND EXTERNAL AGENCIES

AIMS:

- To ensure faith setting leaders and decision makers know of local services, businesses and charities which can offer support for healthy behaviours

OUTCOMES:

- To create a comprehensive plan to host a health fayre focusing on healthy diet and physical activity, so that information and learning can be promoted through the community
- Connections are built between the faith setting and external agencies and organisations which can support the faith setting to promote behaviours for a healthy lifestyle

TARGET PARTICIPANTS:

- Imams, staff, volunteers and management, external agencies

LENGTH OF WORKSHOP:

- 1 hour

EQUIPMENT REQUIRED:

- Pens and paper for taking notes
- Access to internet (smartphones or otherwise) for researching organisations

LOCATION:

- Faith setting or any suitable space where participants can gather

HEALTH AND SAFETY:

- N/A

FOOTWEAR AND CLOTHING:

- N/A

This toolkit is coproduced by Born in Bradford, Bradford Council, and Faith in Communities. For details, see introduction.

And help one another in righteousness and piety,
and do not help one another in sin and aggression.
[Qur'an, 5:2]

One of the two women said, "O my father, hire him. Indeed, the best whom you might hire is the strong and the trustworthy."
[Qur'an, 28:26]

[There are] two blessings in which many people are deceived: good health and free time.
[Al-Bukhari]

Usman Ibn Maz'un was a companion of the Prophet Mohammed (pbuh) who had decided to put himself through some hardship. The Prophet (pbuh) came to learn of this and advised him against this saying, "Indeed, your body has a right over you." [Abu Dawud]

*City of Bradford Metropolitan District Council, and Born in Bradford do not own the Islamic Narrative in the toolkit and maintain this to be the independent work of Mufti Mohammed Zubair Butt. All enquiries relating to the Islamic Narrative should be referred to Mufti Mohammed Zubair Butt directly. For detailed disclaimer, please see page 2.

ACTIVITY 1
Discussion

10 mins

Which local services support healthy diet and physical activity?

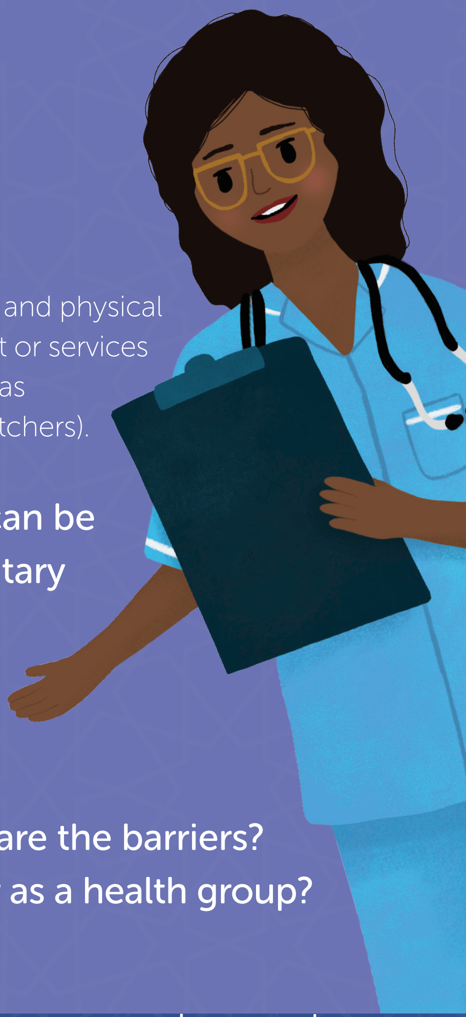

Facilitator leads discussion on the above question.

Ascertain local services available in the community that support healthy diet and physical activity – particularly encouraging participants to identify the informal support or services that may be available in the community (e.g. small community groups such as walking groups, or locally operating charities and businesses e.g. Weight Watchers).

? Can you identify your local health agencies? These can be local authority services, public health services, voluntary sector or charities and businesses operating in the local area (medical, physical and mental health, social, care)

? Can you access these? How? If not, why not – what are the barriers? Can these barriers be overcome by working together as a health group?

ACTIVITY 2
Plan and Organise a Health Event

50 mins

Faith setting to plan and organise a health event to promote healthy lifestyle behaviours (with a focus on food and physical activity) for the congregation by inviting community and Islamic health organisations.

Depending on the size of the faith setting and resources available, this can be either a 'mega health fayre' with large numbers of attendees or 'local health fayre' with smaller numbers. *Whilst this workshop focuses on planning, note the outcome of the workshop includes hosting a health fayre – further action and planning will be required to achieve this.*

Action:

1. Ensure that all actions have a named person responsible for taking them forward, to ensure that this planning progresses and the best event possible can take place. If appropriate, this may involve partnering with another local faith setting to increase available resources. An example plan is available below.

2. Please discuss:
   1. **Planning:** E.g. date and time
   2. **Marketing/promoting:** Consider intended attendees such as local community, local press, local councillors etc
   3. **Finding resources/funds:** Budgeting
   4. **Event day management:** E.g. plans for bad weather if planned for outside, responsible person(s) for health and safety
   5. **Shortlisting/inviting health services/organisations on certain day(s):** Giving particular attention to groups who may not be immediately included, e.g. attendance for those with disabilities, organisations with busy or restricted schedules (can these be confirmed for intended date or must date be flexible to agree when organisations are on board?)

3. Facilitator shares ideas for stalls/activities that a faith setting can organise for the event and encourages participants to think about how they could be involved, e.g. host a stall at the fayre to share their experiences and encourage others to try it. Consider how stalls could engage attendees (e.g. give-it-a-go, samples or info leaflets to hand out):
   - My Islamic New Year Health Resolutions
   - Sunnah Fasting Together
   - Organising a community Eid party in the park/nature/green space
   - Hajj Miles: Long walks (challenging terrain)
   - Sunnah foods (tasting samples) and their benefits
   - Female health (mother and daughter, GP/nurse offers information)

Example Plan for Health Fayre:

Proposed date, time and venue: Saturday 11th November 2023, 14:00, Mosque

Intended attendees: Families and parents from the local area who attend mosque or madrasa

How to promote the event: Social media pages, group communications to parents (e.g. WhatsApp), letter given to children to take home, reminder before/after Friday prayer for 4 weeks leading to event

Budget: Room hire: free. Refreshments (tea/coffee/fruits): £50

Event day management: Named person with knowledge and responsibility for health and safety

Health services/promotion stalls:
- **Invited:** Local GP service *(name of person responsible for contact)*
- **Confirmed?** Yes/No

This toolkit is coproduced by Born in Bradford, Bradford Council, and Faith in Communities. For details, see introduction.

HEALTHY PLACES WORKSHOP 5

TRANSPORT, TRAVEL, AND MANAGING VEHICLES

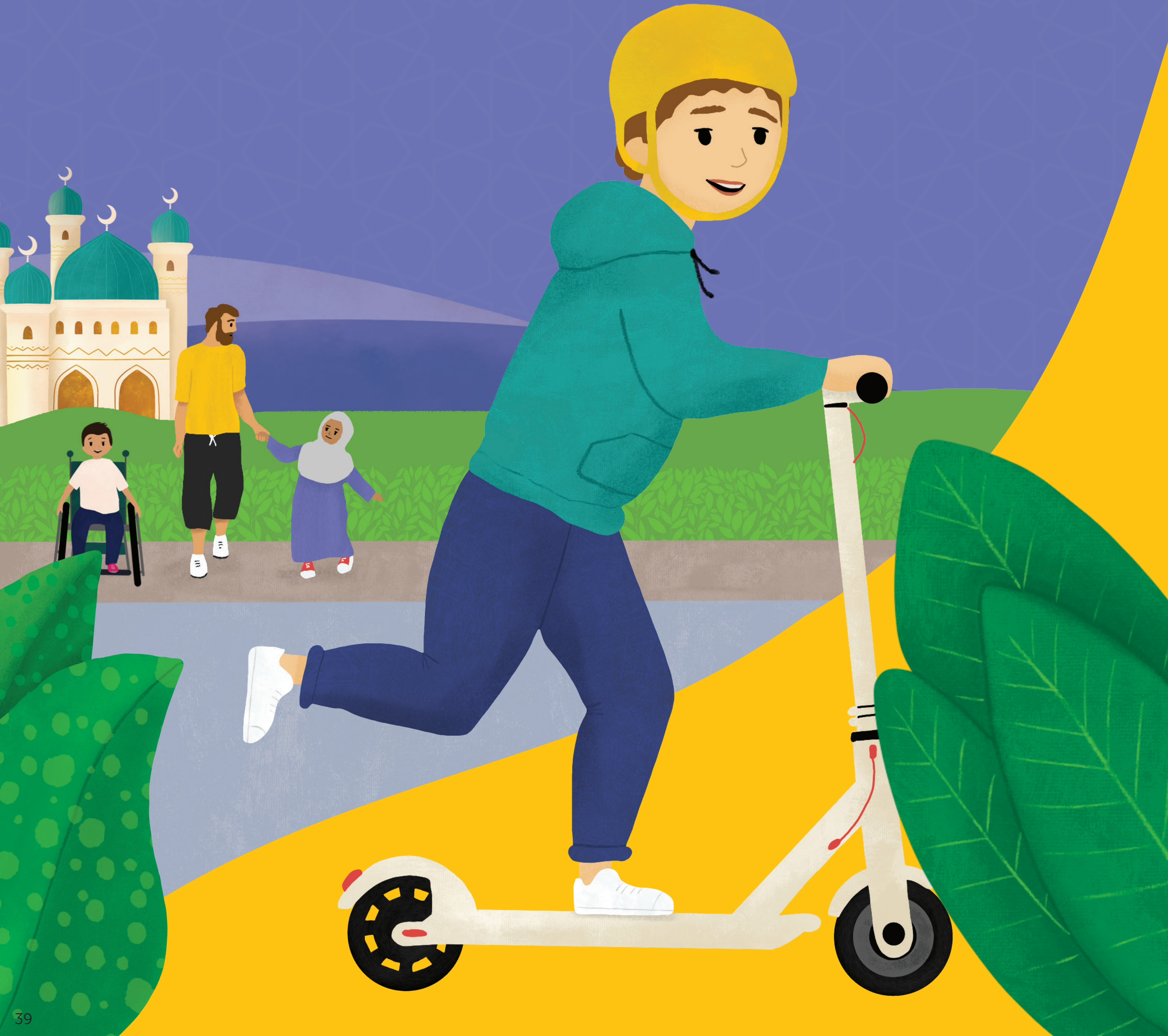

HEALTHY PLACES WORKSHOP 5

TRANSPORT, TRAVEL, AND MANAGING VEHICLES

AIMS:

- Faith setting staff, volunteers, leaders and parents understand the benefits of reducing reliance on motor vehicles as a means of transport

OUTCOMES:

- Develop alternative strategies that local families can use as a means of ensuring children can get to and from the faith setting with reduced reliance on motor vehicles
- Develop plans on how faith setting staff and volunteers can manage the number of vehicles around the madrasa at pick-up and drop-off times

TARGET PARTICIPANTS:

- Parents, staff, volunteers, leaders

LENGTH OF WORKSHOP:

- 1 hour

EQUIPMENT REQUIRED:

- Pens and paper for taking notes

LOCATION:

- Faith setting or any suitable space where participants can gather

HEALTH AND SAFETY:

- N/A

FOOTWEAR AND CLOTHING:

- N/A

NOTE: If you choose to do this workshop, it would also be very beneficial to do **Healthy Places Workshop 6** and **Physical Activity Workshop 5** which focus on promoting safe walking and cycling to faith settings for children and families.

This toolkit is coproduced by Born in Bradford, Bradford Council, and Faith in Communities. For details, see introduction.

And do not throw [yourselves] with your [own] hands in to destruction.
[Qur'an, 2:195]

[There are] two blessings in which many people are deceived: good health and free time.
[Al-Bukhari]

Abu Huraira reported that the Prophet (pbuh) said, "Whoever goes to the mosque in the morning and evening, Allah will prepare for him a place in Paradise for every morning and evening."
[Al-Bukhari]

O you who believe! When the call is made for prayer on the day of congregation [Friday], hasten to the remembrance of Allah and leave off trade. That is better for you, if you but knew.
[Qur'an, 62:9]

*City of Bradford Metropolitan District Council, and Born in Bradford do not own the Islamic Narrative in the toolkit and maintain this to be the independent work of Mufti Mohammed Zubair Butt. All enquiries relating to the Islamic Narrative should be referred to Mufti Mohammed Zubair Butt directly. For detailed disclaimer, please see page 2.

The Prophet (pbuh) told us that the entire process of attending the mosque, including every step taken to the house of God, is rewarded.

He who purifies himself (performs Wudhu) at his home and then walks to a house from the houses of Allah (mosque) so that he may discharge an obligation from the obligations of Allah (perform an obligatory prayer), one of his two steps will wipe out a sin and the other (step) will elevate one rank (in Jannah).

[Muslim]

Aaisha, the wife of the Prophet (pbuh) reports, "I was with the Prophet (pbuh) on a journey whilst I was [still] young. The Prophet (pbuh) said to his companions, 'Come forward!' So they came forward. Then he said [to me], 'Come, let me race with you.' So I raced with him and raced ahead of him on foot. After some time, I went again on a journey with him and he said to his companions, 'Come forward!' Then he said, 'Come, let me race with you.' I had forgotten what had happened [before] and had gained weight and so I said, How can I race with you, O Messenger of Allah! Whilst I am in this state?' He replied, 'You must do so.' So I raced him and he beat me in the race. Then he said,' This is for that win.'"

[Sunan al-Baihaqi]

In another Hadith, his cousin Ali described his manner of walking as follows: "When he walked, he lifted his leg with vigour. As though he was descending from a high place."

[Al-Mustadrak]

This toolkit is coproduced by Born in Bradford, Bradford Council, and Faith in Communities. For details, see introduction.

ACTIVITY 1
Discussion

10 mins

Facilitator to lead discussion, posing the following questions for participants:

- **Why do we love cars?**

  Is it due to convenience, speed, habit, and/or any other factors?

- **What are the benefits of walking to the faith setting instead of going in a car?**

  Ensure that a wide range of social and health benefits of walking to the faith setting are emphasised, for example mental and physical health, environmental impact or the opportunity to spend quality time together.

- **What are the barriers in walking to the faith setting?**

  Consider a lack of time for busy families, limited mobility, or simply a habit of using cars.

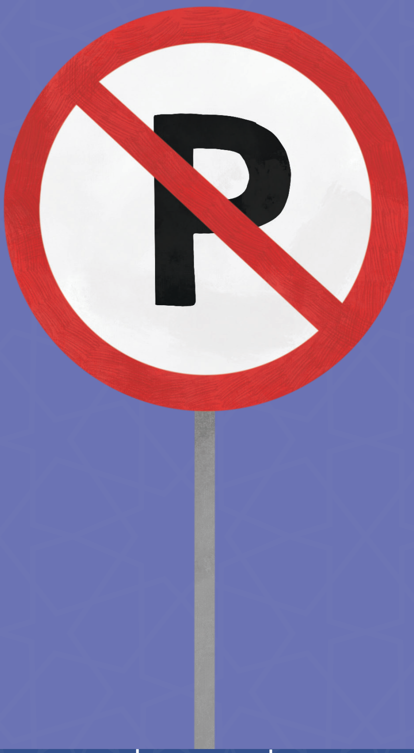

ACTIVITY 2
Discussion

20 mins

How can we manage motor vehicles locally?

In small groups participants have a discussion on the following points before sharing ideas back in the group:

- How can participants reduce dependency on vehicles whilst picking up or dropping off children?
- How can the faith setting promote vehicle management messages through children to parents?

Facilitators share the following ideas and participants can consider practicalities of implementation within their local setting:

- Car free day
- Vehicle management (dedicated volunteers at faith setting to monitor car flow before and after madrasa time)
- Restricted access to madrasa parking space
- Walk instead of driving
- Park outside half a mile or a mile radius from the faith setting
- Cycling stand
- Parking charges or parking permits (optional)

ACTIVITY 3
Calculation

10 mins

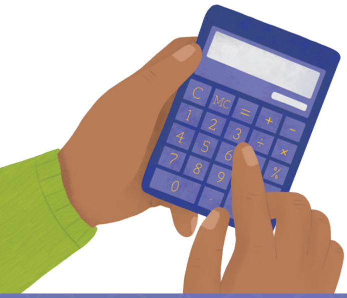

Work out the associated petrol costs for the distance driven.

Parents calculate how much money they can save by not using a vehicle to get to the faith setting over one week or a month.

ACTIVITY 4
Discussion and Action Plan

20 mins

This discussion can be focused on the health group once other workshop attendees have left.

Action:

1. Group discussion on securing resources for vehicle management. Facilitators can introduce available programmes in the local area.
2. Health group to make a plan to connect with cycling organisations and obtain funding.
3. Faith setting management to contact/request a Parking Warden to regularly visit the street, to deter excessive vehicle use.

We are family BORNINBRADFORD
JU:MP
FAITH IN COMMUNITIES
LIVING WELL
City of BRADFORD METROPOLITAN DISTRICT COUNCIL
Department of Health & Social Care
BRADFORD INSTITUTE FOR HEALTH RESEARCH | MAKING RESEARCH REAL
SPORT ENGLAND
Local Government Association

HEALTHY PLACES WORKSHOP 6

SAFE WALKING AND CYCLING

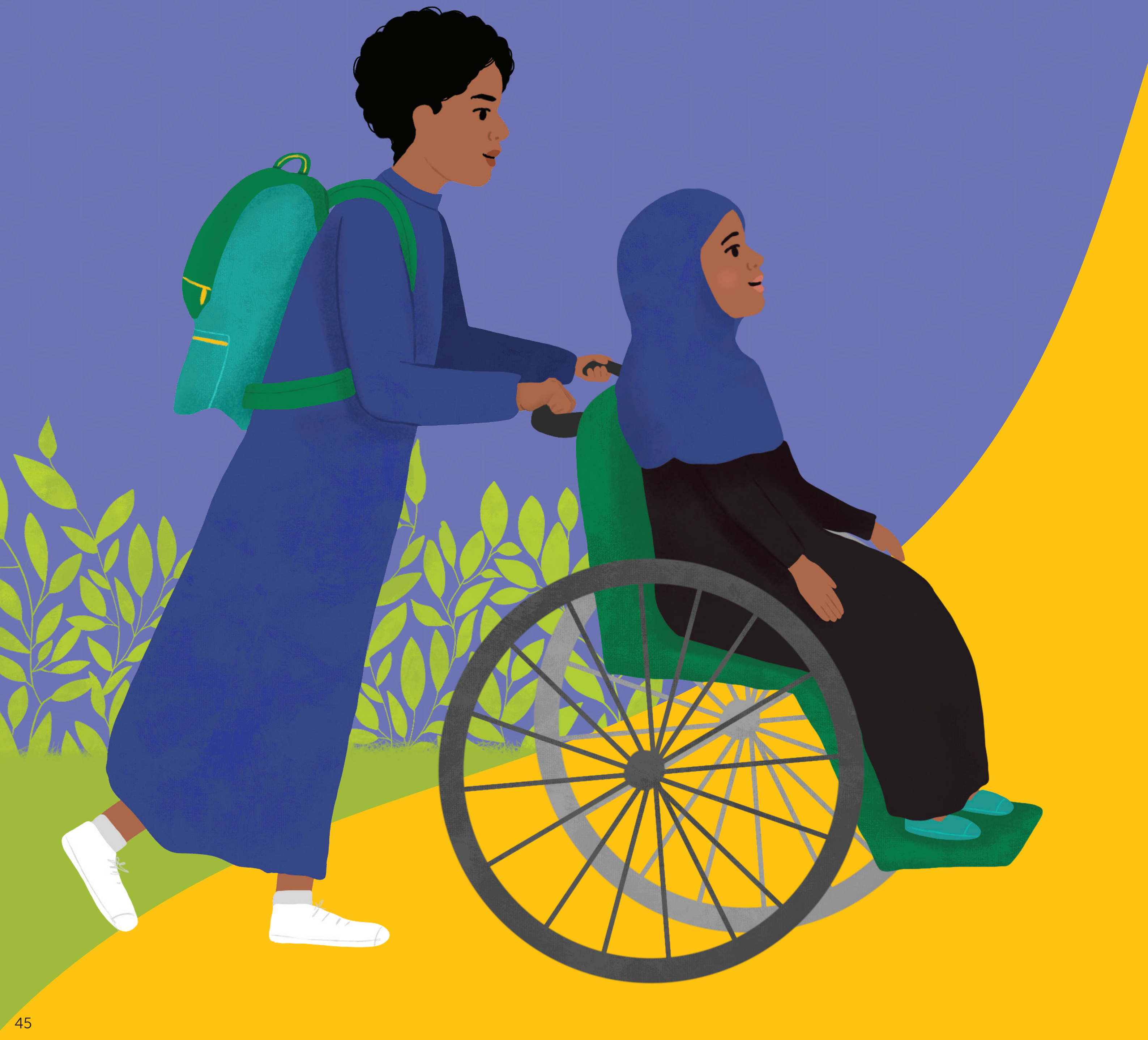

HEALTHY PLACES WORKSHOP 6

SAFE WALKING AND CYCLING

AIMS:

- To design a safe route that can be used by children when actively travelling to and from the faith setting
- Increase safety awareness for children and families when walking and cycling or scooting

OUTCOMES:

- Children and families feel confident in children actively travelling (e.g. walking, cycling or scooting) to and from the faith setting
- Children and families feel safe when walking, cycling or scooting around their community; they know safe crossing points for busy roads and routes that avoid areas of anti-social behaviour

TARGET PARTICIPANTS:

- Children, parents, staff

LENGTH OF WORKSHOP:

- 50 minutes

EQUIPMENT REQUIRED:

- Pens and paper (ideally flip-chart/larger sized paper)
- Smartphone/tablet/laptop with internet access for showing Google Street View, or print-outs of the google satellite view for the group to draw on

LOCATION:

- Faith setting or any suitable space where participants can gather

HEALTH AND SAFETY:

- N/A

FOOTWEAR AND CLOTHING:

- N/A

This toolkit is coproduced by Born in Bradford, Bradford Council, and Faith in Communities. For details, see introduction.

And help one another in righteousness and piety, and do not help one another in sin and aggression.
[Qur'an, 5:2]

And hold firmly to the rope of Allah all together and do not be divided.
[Qur'an, 3:103]

And do not throw [yourselves] with your [own] hands in to destruction.
[Qur'an, 2:195]

The Prophet Muhammad (pbuh) would naturally walk at a vigorously brisk pace and his close companions found it difficult to keep pace with him. One of his close companions, Abu Huraira, described his walking on one occasion saying, "And I did not see anyone faster in his manner of walking than the Messenger of Allah pbuh, as if the earth was folded for him. We would exert ourselves [to keep up with him] whilst he was not troubled."
[Ibn Hibban]

Usman Ibn Maz'un was a companion of the Prophet Mohammed (pbuh) who had decided to put himself through some hardship. The Prophet (pbuh) came to learn of this and advised him against this, saying, "Indeed, your body has a right over you."
[Abu Dawud]

*City of Bradford Metropolitan District Council, and Born in Bradford do not own the Islamic Narrative in the toolkit and maintain this to be the independent work of Mufti Mohammed Zubair Butt. All enquiries relating to the Islamic Narrative should be referred to Mufti Mohammed Zubair Butt directly. For detailed disclaimer, please see page 2.

ACTIVITY 1
Discussion

10 mins

Facilitator and Imam lead discussion on the benefits of walking to the faith setting and whether or not (and why not) they feel safe walking.

The following points can be discussed:

- Why is physical activity important for families?
- What are the social benefits of walking and exploring your neighbourhood? (Improved confidence for children and families, understanding local area, sense of direction and being streetwise)
- What are the benefits of walking as part of a group for collective safety, especially for young girls?
- Do the children feel safe walking to and from the faith setting, and why/ why not?

ACTIVITY 2
Creating a Route Map

20 mins

Participants create a potential route map for walking to the faith setting from up to four directions (depending on size of faith setting).

Action:

1. Facilitator shows Google Maps with 'street view'. Whilst going through the potential route, consider any points where health and safety may be a concern, including areas where road safety should be considered as well as any areas known as risk for anti-social behaviour.

2. Also consider the suitability of the route for children with physical disabilities who may use physical mobility supports (e.g. walking sticks or wheelchair), or that may be blind or deaf.

3. If the route contains many areas of concern, consider how the route can be adjusted.

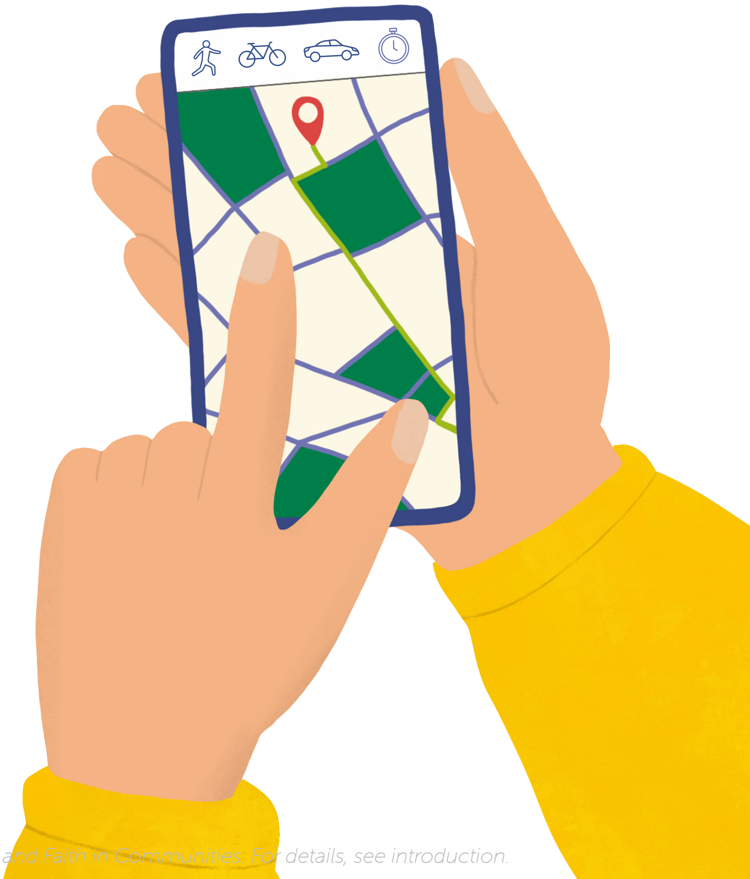

ACTIVITY 3
Linking Walking Routes with Green Spaces

10 mins

Link the finalised safe walking routes with nearby green space if possible (to be used as an activity whenever possible).

Action:

1. Facilitator shares benefits of green spaces (e.g. for mental health, being more active, playing, socialising).
2. Facilitators can highlight spots that could be used as picnic spaces or for physical activity.
3. Consider safety in green spaces, particularly availability of lighting during winter months or if they are linked with anti-social behaviour.
4. Highlight this on the map to encourage awareness of safety in areas, linking into Activity 4.

ACTIVITY 4
Discussion

10 mins

Ensure safety of children whilst walking and actively travelling, and highlight points on walking route where safety measures are required. For example, crossing points for roads/how to safely cross any roads, avoidance of areas known for anti-social behaviour (those identified from Activity 3), areas of low lighting, or those which may be icy during winter months, etc.

Discussion points can include:

- How can children assess safety for themselves? (Knowledge of nature and increased awareness and confidence in their neighbourhood)
- High visibility jackets and walking as group
- Possible use of pedometers and daily step count

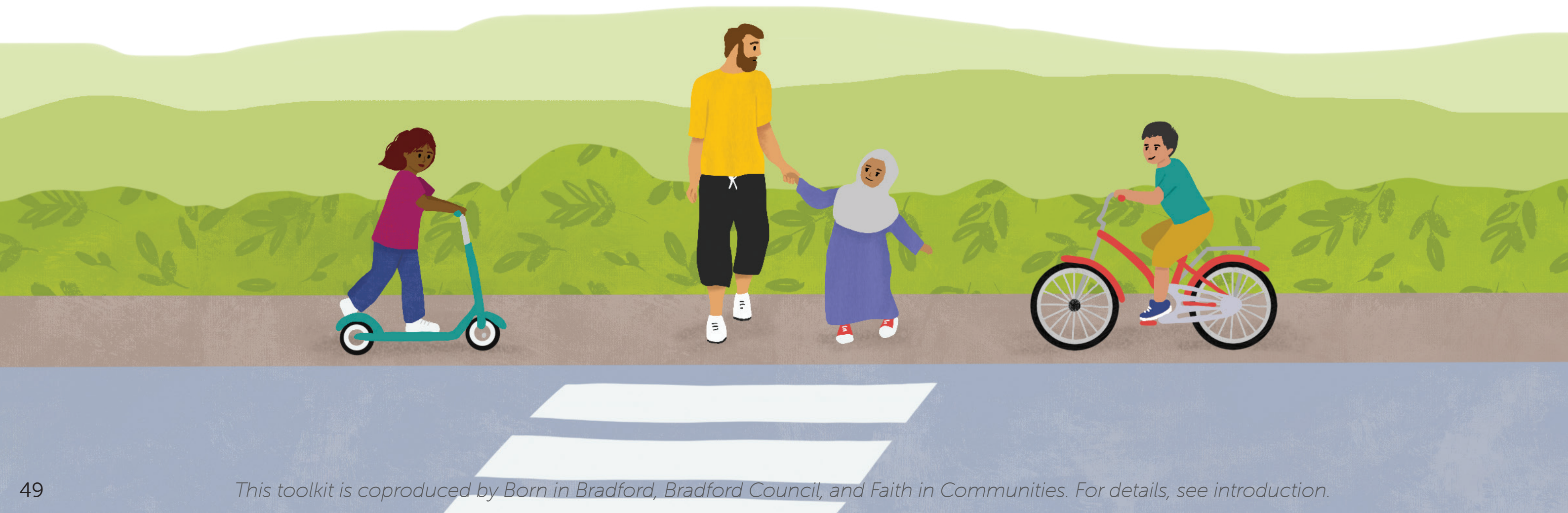

HEALTHY PLACES WORKSHOP 7

WORKING TOGETHER AND BUILDING RELATIONSHIPS

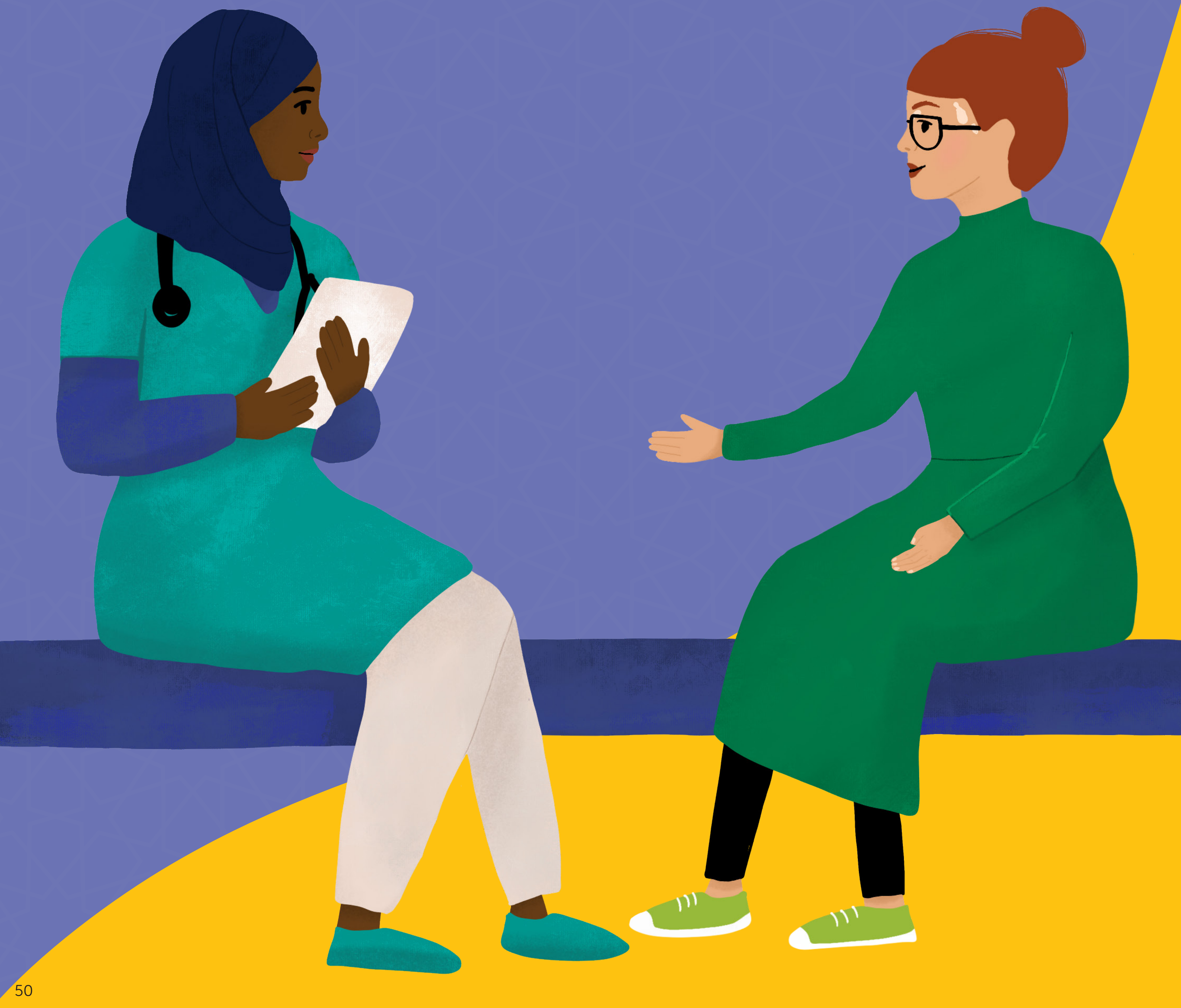

HEALTHY PLACES WORKSHOP 7

WORKING TOGETHER AND BUILDING RELATIONSHIPS

AIMS:

- The health group, including faith setting leaders and decision makers, understand the areas that may need external support/funding in order to develop/implement plans

OUTCOMES:

- Faith setting leaders and decision makers know how to access funding opportunities and support to submit funding applications, for health promotion activities

TARGET PARTICIPANTS:

- Staff, management, leaders, local organisations

LENGTH OF WORKSHOP:

- 50 minutes

EQUIPMENT REQUIRED:

- Notebooks/technology for researching and taking notes on planning

LOCATION:

- Faith setting or any suitable space where participants can gather

HEALTH AND SAFETY:

- N/A

FOOTWEAR AND CLOTHING:

- N/A

This toolkit is coproduced by Born in Bradford, Bradford Council, and Faith in Communities. For details, see introduction.

And do not throw [yourselves] with your [own] hands in to destruction.
[Qur'an, 2:195]

One of the two women said, "O my father, hire him. Indeed, the best whom you might hire is the strong and the trustworthy."
[Qur'an, 28:26]

[There are] two blessings in which many people are deceived: good health and free time.
[Al-Bukhari]

The Prophet Muhammad (pbuh) would naturally walk at a vigorously brisk pace and his close companions found it difficult to keep pace with him. One of his close companions, Abu Huraira, described his walking on one occasion saying, "And I did not see anyone faster in his manner of walking than the Messenger of Allah pbuh, as if the earth was folded for him. We would exert ourselves [to keep up with him] whilst he was not troubled."
[Al-Shamail Al-Muhammadiyya, Ibn Hibban]

*City of Bradford Metropolitan District Council, and Born in Bradford do not own the Islamic Narrative in the toolkit and maintain this to be the independent work of Mufti Mohammed Zubair Butt. All enquiries relating to the Islamic Narrative should be referred to Mufti Mohammed Zubair Butt directly. For detailed disclaimer, please see page 2.

And help one another in righteousness and piety, and do not help one another in sin and aggression.
[Qur'an, 5:2]

And hold firmly to the rope of Allah all together and do not be divided.
[Qur'an, 3:103]

Whilst offering advice to a man, the Prophet Mohammed (pbuh) said, “Value five [things] before five [other things]: Your youth before your old age, your health before your illness, your affluence before your poverty, your availability before your occupation, and your life before your death.
[Al-Mustadrak]

Usman Ibn Maz'un was a companion of the Prophet Mohammed (pbuh) who had decided to put himself through some hardship. The Prophet (pbuh) came to learn of this and advised him against this saying, “Indeed, your body has a right over you.”
[Abu Dawud]

ACTIVITY 1
Discussion

10 mins

Facilitator leads a discussion on the benefits of partnership working and building relationships with external agencies/organisations.

Discussion points can include:

- Pooling resources by working with external organisations
- Specific benefits for your faith setting from partnering with other organisations, and what this can help your faith setting to achieve (e.g. capacity building to be able to offer more to the community)

ACTIVITY 2
Discussion

10 mins

Discuss the needs of the local community and what changes are needed, to encourage physical activity and healthy eating, both within the faith setting and within the local community (consider the priorities and actions developed through **Healthy Places Workshop 1**).

Participants should discuss and consider:

- Health and wellbeing needs and areas of improvement for faith setting
- Identify available funding and organisations/donors
- Finding links with funding bodies/networking

Facilitator shares available local programmes which faith settings can access for information.

Examples from Bradford include:

- JU:MP Programme (Sports England)/Born in Bradford
- Living Well
- Faith in Communities
- Bradford Provider Alliance
- Public Health – Bradford Council
- NHS England
- NHS West Yorkshire Health and Care Partnership
- The Health Foundation

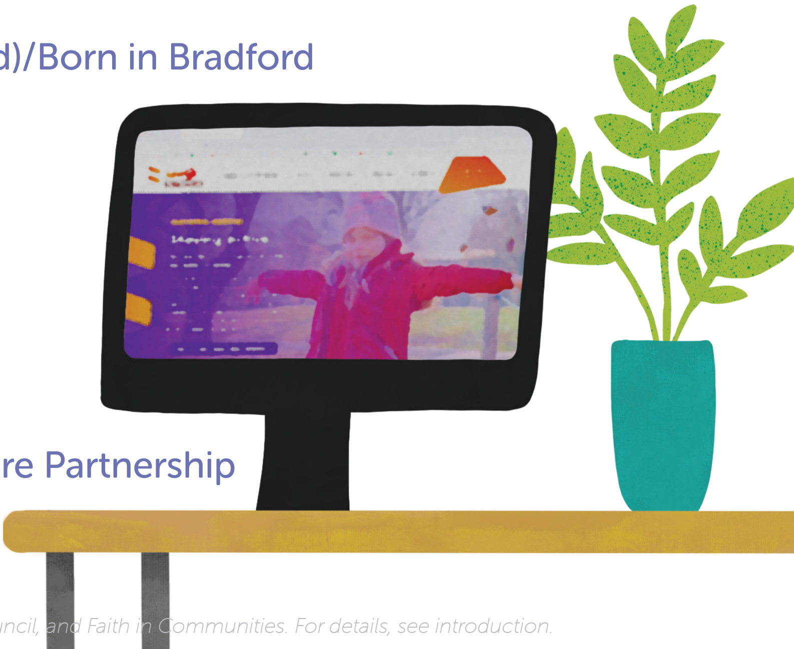

This toolkit is coproduced by Born in Bradford, Bradford Council, and Faith in Communities. For details, see introduction.

ACTIVITY 3
Group Training

30 mins

Identify and plan group training activities that participants can take part in, to build the skills and capacity within the faith setting.

These are transferable skills that can be taken across other areas of faith setting delivery and learning, not only connected to delivery of the toolkit but also allows for wider training and development of staff and volunteers.

Steps to identify training can include:

1. Link up with existing delivery and implementation programme (e.g. Living Well Academy or JU:MP in Bradford).
2. Identify dedicated members of the health group to seek their commitment for training.
3. Request organisation's availability and seek training slot.
4. Attend and participate in training (either where organisation conducts, or by inviting organisation to your faith setting).

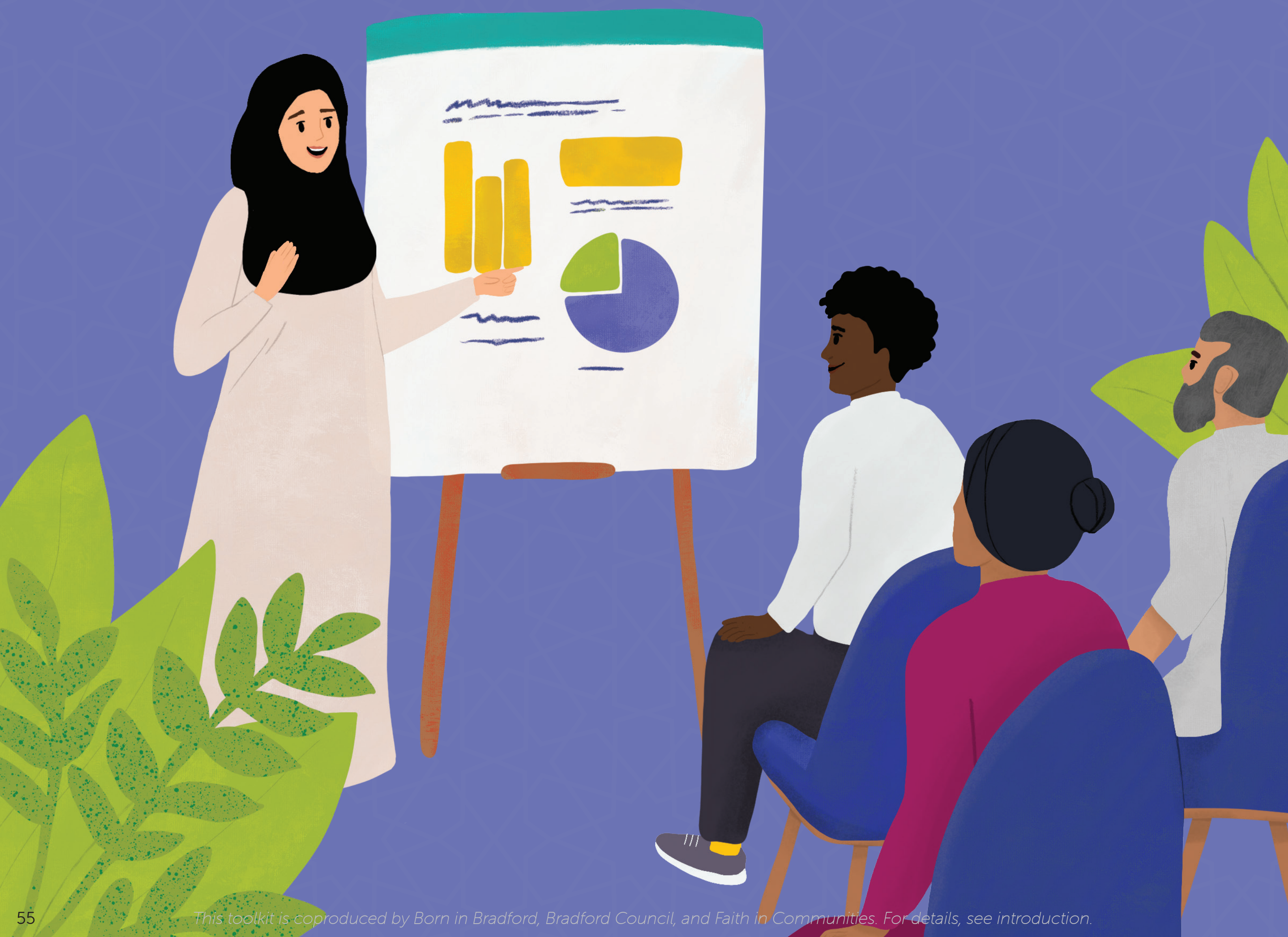

This toolkit is coproduced by Born in Bradford, Bradford Council, and Faith in Communities. For details, see introduction.

We are family BORNINBRADFORD | JU:MP | FAITH IN COMMUNITIES | LIVING WELL | City of BRADFORD METROPOLITAN DISTRICT COUNCIL

Department of Health & Social Care | BRADFORD INSTITUTE FOR HEALTH RESEARCH MAKING RESEARCH REAL | SPORT ENGLAND | Local Government Association

HEALTHY PLACES WORKSHOP 8

COLLABORATIONS AND APPLYING FOR SMALL GRANTS

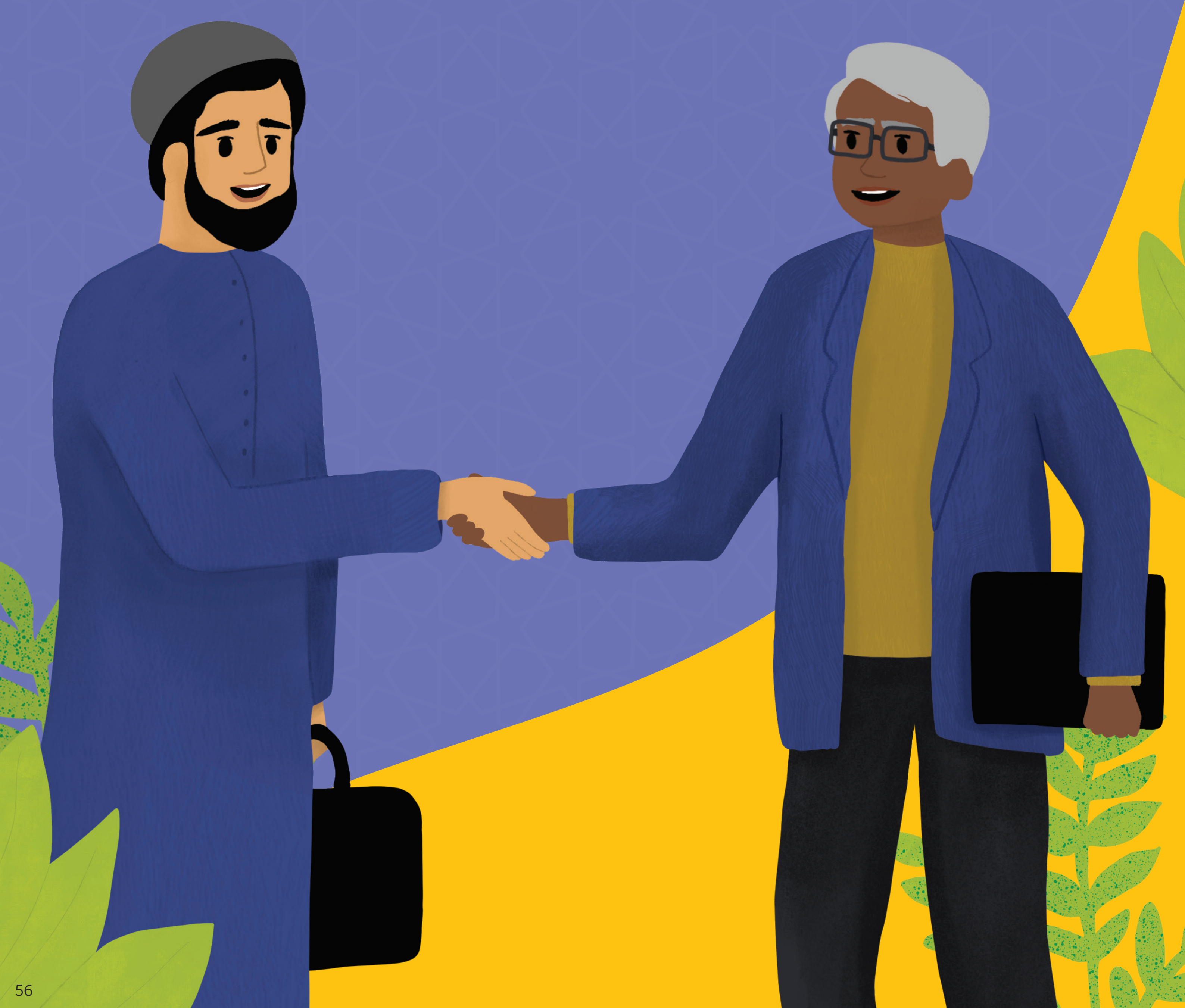

COLLABORATIONS AND APPLYING FOR SMALL GRANTS

AIMS:

- The health group, including faith setting leaders and decision makers aim to apply for available small grants

OUTCOMES:

- Faith setting leaders and decision makers know how to collaborate with external agencies and apply for small grants

TARGET PARTICIPANTS:

- Staff, management, leaders, local organisations

LENGTH OF WORKSHOP:

- 45 minutes

EQUIPMENT REQUIRED:

- Notebooks/technology for researching and taking notes on planning

LOCATION:

- Faith setting or any suitable space where participants can gather

HEALTH AND SAFETY:

- N/A

FOOTWEAR AND CLOTHING:

- N/A

ISLAMIC NARRATIVE

And do not throw [yourselves] with your [own] hands in to destruction.
[Qur'an, 2:195]

One of the two women said, "O my father, hire him. Indeed, the best whom you might hire is the strong and the trustworthy."
[Qur'an, 28:26]

[There are] two blessings in which many people are deceived: good health and free time.
[Al-Bukhari]

*City of Bradford Metropolitan District Council, and Born in Bradford do not own the Islamic Narrative in the toolkit and maintain this to be the independent work of Mufti Mohammed Zubair Butt. All enquiries relating to the Islamic Narrative should be referred to Mufti Mohammed Zubair Butt directly. For detailed disclaimer, please see page 2.

And help one another in righteousness and piety, and do not help one another in sin and aggression.
[Qur'an, 5:2]

And hold firmly to the rope of Allah all together and do not be divided.
[Qur'an, 3:103]

Whilst offering advice to a man, the Prophet Mohammed (pbuh) said, "Value five [things] before five [other things]: Your youth before your old age, your health before your illness, your affluence before your poverty, your availability before your occupation, and your life before your death.
[Al-Mustadrak]

Usman Ibn Maz'un was a companion of the Prophet Mohammed (pbuh) who had decided to put himself through some hardship. The Prophet (pbuh) came to learn of this and advised him against this saying, "Indeed, your body has a right over you."
[Abu Dawud]

This toolkit is coproduced by Born in Bradford, Bradford Council, and Faith in Communities. For details, see introduction.

ACTIVITY 1
Inviting Organisations to the Faith Setting

15 mins

Develop a plan to invite organisations to your faith setting, to introduce their services and messages.

Facilitator discusses the following steps required by participants for the plan to be implemented successfully (refer also to **Workshop 4**, with emphasis on event for multiple organisations).

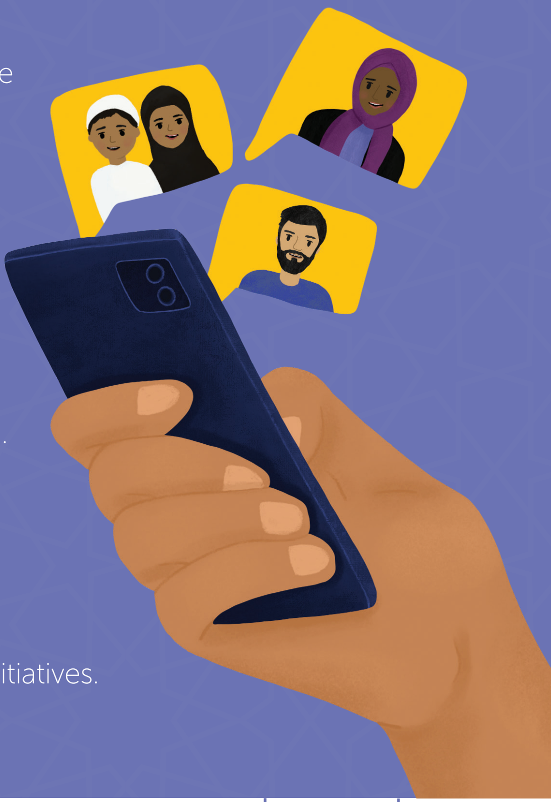

Action:

1. Health group makes a plan and identifies organisations they would like to invite.
2. Assign a volunteer/staff to communicate with the organisation.
3. Invite parents and children to attend the event.
4. Organisation presents and delivers their message.
5. Make partnership and collaboration arrangements for future initiatives.

ACTIVITY 2
Developing a Funding Proposal

30 mins

Introduction on how to develop a small funding proposal.

Action:

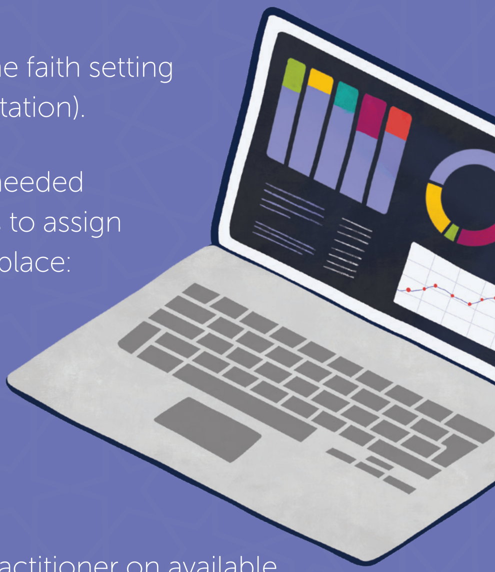

1. Participants identify an area to focus on, relevant to the needs of the faith setting and the community (e.g. healthy diet or physical activity implementation).
2. Facilitator should encourage participants to think through what is needed before starting the funding application, and encourage participants to assign the following actions for development if these are not currently in place:
   1. Funder criteria
   2. Bank account/registration
   3. Team/volunteers/groups
   4. Beneficiaries (children & families)
   5. Plan of action
3. Talk to the relevant community engagement manager or health practitioner on available funding opportunities in your area. Contact Faith in Communities in Bradford if you need any further assistance or support from the Living Well Community Health Development team, at *bit.ly/3HQKq52*.

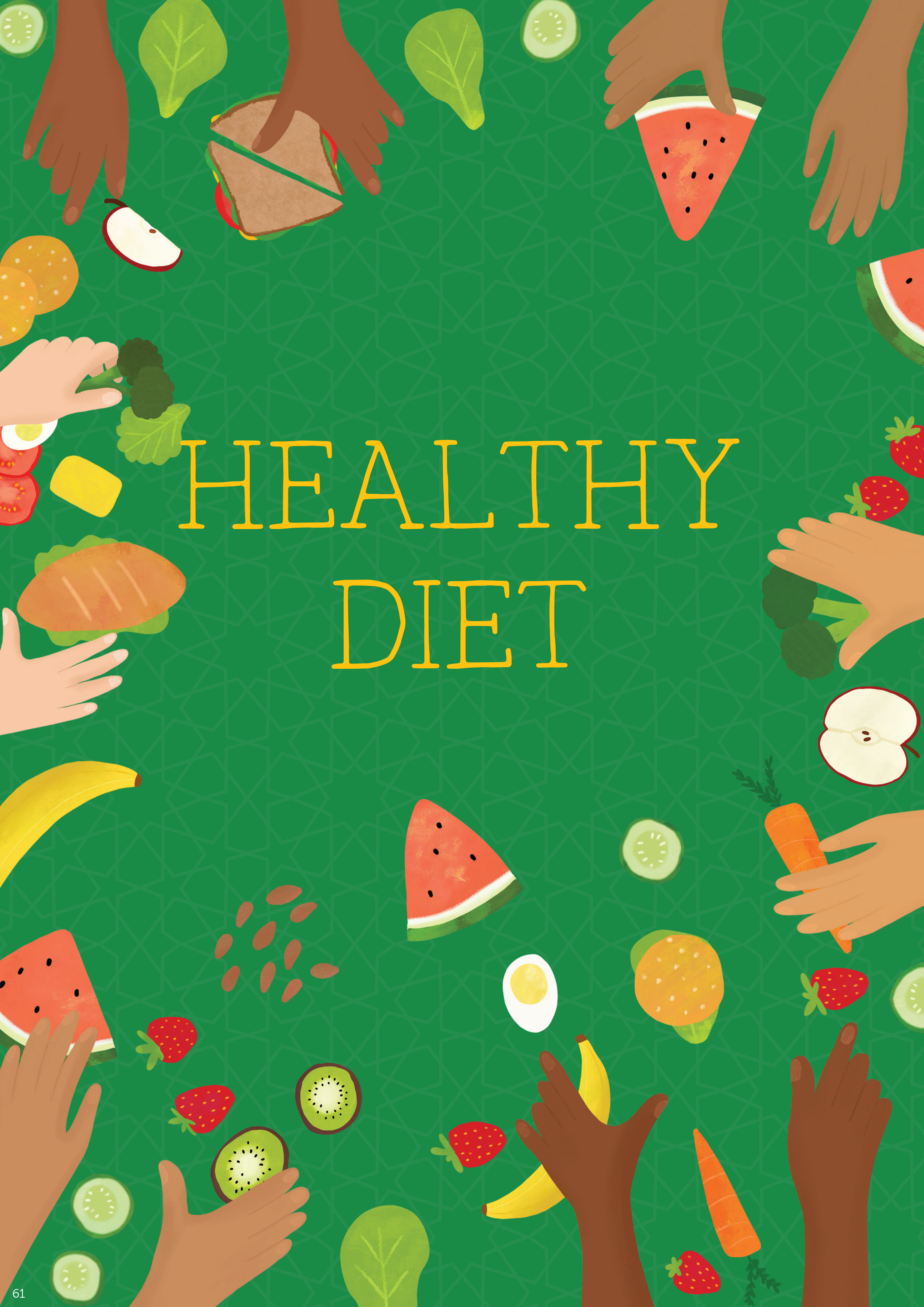

HEALTHY DIET

HEALTHY DIET

This section will focus on healthy diet workshops to be delivered by trained faith setting staff/ local champions for a healthy diet for children and families. The healthy diet workshops should initially be delivered by a subject matter expert or by an externally funded community engagement manager and attended by staff or volunteers of the faith setting. This will enable the staff of the faith setting or any volunteer designated by the health group to be trained in the delivery of healthy diet workshops by a subject matter expert or a community engagement manager. The staff or a volunteer in the faith setting can deliver healthy diet workshops on subsequent occasions after they are trained, and are confident in doing so.

AIMS:

- To motivate children and families to learn and adopt healthy dietary habits
- Children and families to become more confident in preparing nutritious food
- Children to learn etiquette when eating or serving food
- Children and families understand why portion size is important and know what appropriate portion sizes look like for children
- Education on healthy diet and shopping

WORKSHOPS:

1. Introduction to Nutrition
2. Creative Kitchens
3. Drinking Water
4. Mealtimes and Manners
5. Portion Size
6. Shopping and Food Labels
7. Healthy Snacking

NOTE:

This section contains a cooking activity with children (**Workshop 2: Creative Kitchens**). In order to run a cooking session, the facilitator must hold a Food Safety and Hygiene Level 2 Certificate. This is a basic food hygiene course with an approved certificate, which ensures that anyone working in an environment where food is cooked, prepared or handled complies fully with current UK legislation.

Any of these accredited courses are suitable and cost under £20:

- *food-safety.org.uk*
- *foodsafetyatwork.co.uk/level-2-food-safety*
- *reed.co.uk/courses/food-hygiene-level-2/162300*

We are family BORNINBRADFORD
JU:MP
FAITH IN COMMUNITIES
LIVING WELL
City of BRADFORD METROPOLITAN DISTRICT COUNCIL
Department of Health & Social Care
BRADFORD INSTITUTE FOR HEALTH RESEARCH — MAKING RESEARCH REAL
SPORT ENGLAND
Local Government Association

HEALTHY DIET WORKSHOP 1

INTRODUCTION TO NUTRITION

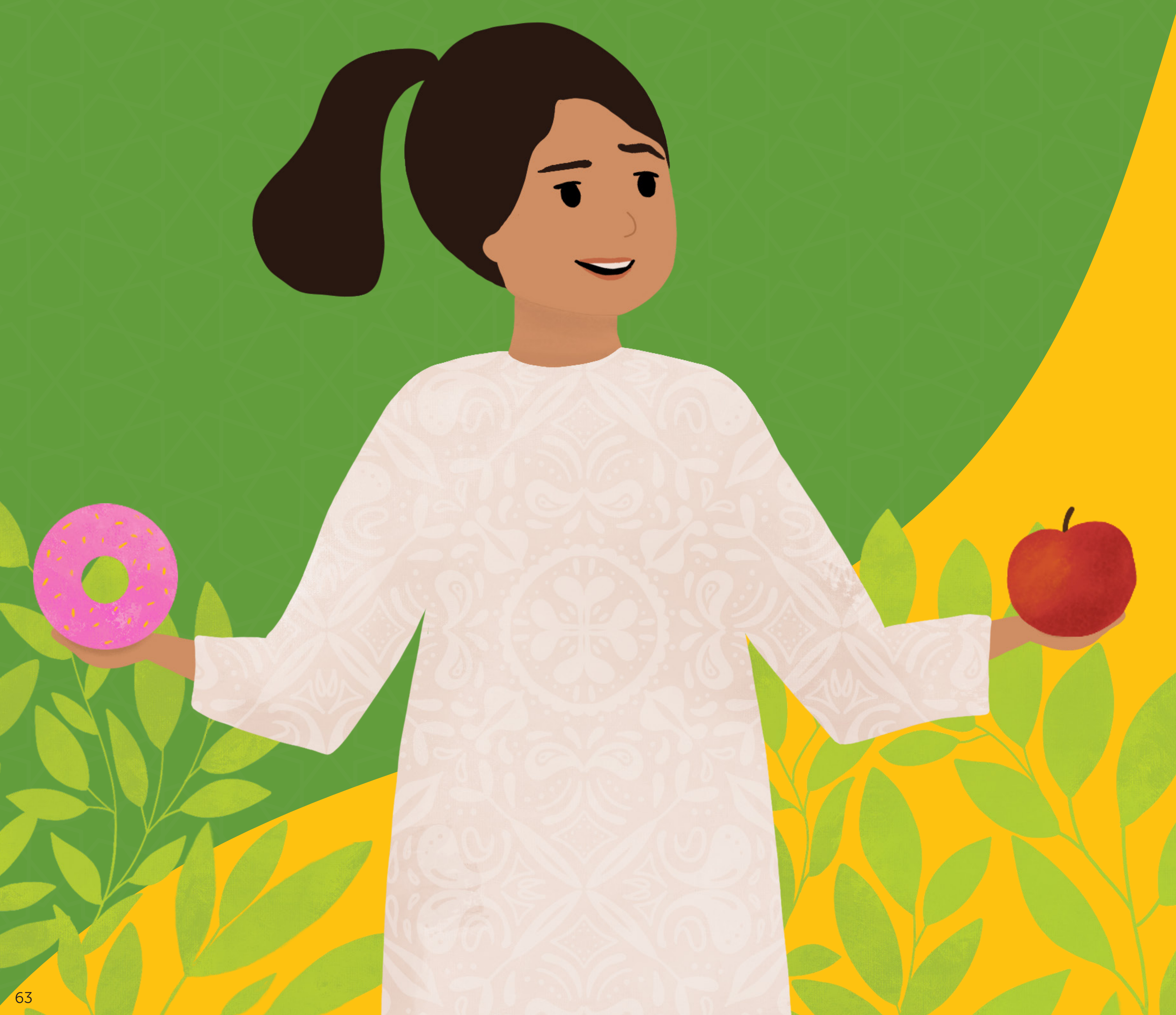

HEALTHY DIET WORKSHOP 1

INTRODUCTION TO NUTRITION

AIMS:

- To introduce the Eatwell Guide
- To learn about how food fuels our bodies
- To make healthy changes to a recipe

OUTCOMES:

- Children and families will be encouraged to read and follow a recipe, understanding where healthy substitutions can be made.
- Children will understand basic food hygiene, kitchen safety and origin of foods
- Children will learn which foods belong in each food group on the Eatwell guide

TARGET PARTICIPANTS:

- Parents and children

LENGTH OF WORKSHOP:

- 50 minutes

EQUIPMENT REQUIRED:

- Pens and paper
- Copies of the Eatwell Guide/South Asian Eatwell Guide (blank version and complete version)
- Printouts of popular recipes (for children to adapt)

LOCATION:

- Faith setting or any suitable space where participants can gather

HEALTH AND SAFETY:

- N/A

FOOTWEAR AND CLOTHING:

- N/A

This toolkit is coproduced by Born in Bradford, Bradford Council, and Faith in Communities. For details, see introduction.

ISLAMIC NARRATIVE

And He (Allah) makes lawful for them pure, wholesome things and He makes unlawful for them noxious things.
[Qur'an, 7:157]

And eat and drink but do not be excessive. Indeed, He does not like those who go to excess.
[Qur'an, 7:31]

The Prophet (pbuh) said, "Eat, drink and spend in charity without going to excess nor ostentation."
[Al-Mustadrak]

Jabir reported, the Messenger of Allah (pbuh) said, "The food of one person is enough for two people, the food of two people is enough for four, and the food of four is enough for eight."
[Muslim]

*City of Bradford Metropolitan District Council, and Born in Bradford do not own the Islamic Narrative in the toolkit and maintain this to be the independent work of Mufti Mohammed Zubair Butt. All enquiries relating to the Islamic Narrative should be referred to Mufti Mohammed Zubair Butt directly. For detailed disclaimer, please see page 2.

ACTIVITY 1
Introduction to the Eatwell Guide

20 mins

Introduce the Eatwell Guide by asking children to complete the following two activities:

1. Using a blank copy of the Eatwell Guide by the NHS, ask children to guess which food group should go into each segment (5 minutes).

2. Ask children to draw in their own carbohydrates, fruit and vegetables, protein, dairy products, and a small section for spreads and oils (15 mins).

Use the Eatwell and South Asian Eatwell Guides which are available at *bit.ly/3IPTh88,* if children are not sure what items to draw into different sections.

3. Provide children with a completed copy of the Eatwell Guide and South Asian Eatwell Guide from *bit.ly/42hqT5J* and *bit.ly/42kaXiW*

ACTIVITY 2
Discussion

15 mins

Facilitator leads a discussion on the different ways that recipes can be made healthier by thinking about ingredients and cooking techniques.

For each of the items below, the facilitator should ask participants which substitutions they think could be made and what would be appropriate, making sure the following is covered:

- **FAT:** Reduce the saturated fat content by using less oil, ghee or butter. A small amount of unsaturated fat such as extra virgin olive oil or rapeseed oil can be used instead.

  Eating foods high in saturated fat can raise cholesterol levels and increase the risk of heart disease. Unsaturated fats found in nuts, olive oil, fish and avocados should be included as part of a healthy, balanced diet as they contain essential vitamins and minerals for growth and development.

- **SALT:** You can reduce salt or remove it completely. Swap salt for herbs or spices to make dishes tastier. Reducing salt can prevent high blood pressure and heart disease.

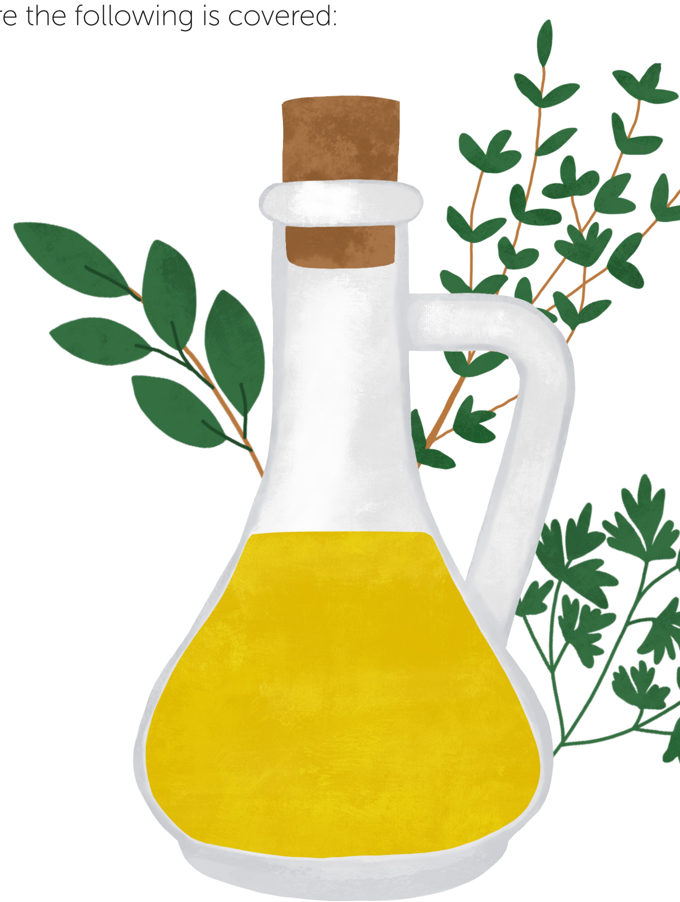

This toolkit is coproduced by Born in Bradford, Bradford Council, and Faith in Communities. For details, see introduction.

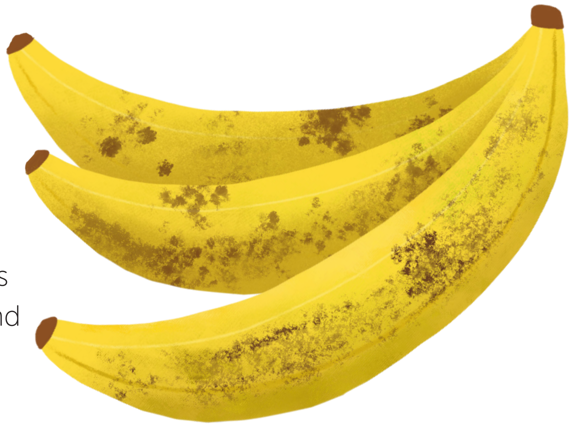

- **SUGAR:** It is important to reduce food and drinks high in sugar. Swap sugar for a small amount of date paste or use fruit to sweeten a recipe. Ripe bananas can be used, which will add vitamins and nutrients as well as sweetness to the recipe. Too much sugar can lead to tooth decay and diabetes.

- **FIBRE:** You can increase the amount of fibre in a recipe by using whole wheat pasta or whole wheat flour instead of the plain white options. Fibre helps us to digest food, and reduces the risk of constipation.

- **COOKING TECHNIQUE:** You can also change the cooking technique. Instead of frying, try to grill, poach, steam, boil or sauté. If you do fry, use a small amount of oil spray or a non-stick pan.

ACTIVITY 3
Adapting Existing Recipes

15 mins

Based on the discussion from Activity 2 and the Eatwell guides from Activity 1, participants should think about how recipes they use might be changed to be healthier.

This can be a themed session e.g. cooking a healthy breakfast, making a healthy packed lunch, etc.

Facilitators may also ask parents or children to bring in their favourite recipes to cook at home, to look at how they can be adapted.

Ask children:

- How many portions of fruit or vegetables does your recipe contain?
- How can you add more fibre into the recipe?
- Is there an ingredient from each section of the Eatwell Guide?
- Is there a source of protein? (Savoury recipes)
- How can we add more vitamins and minerals into the recipe?
- Can you reduce the amount of fat, salt and sugar in the recipe?

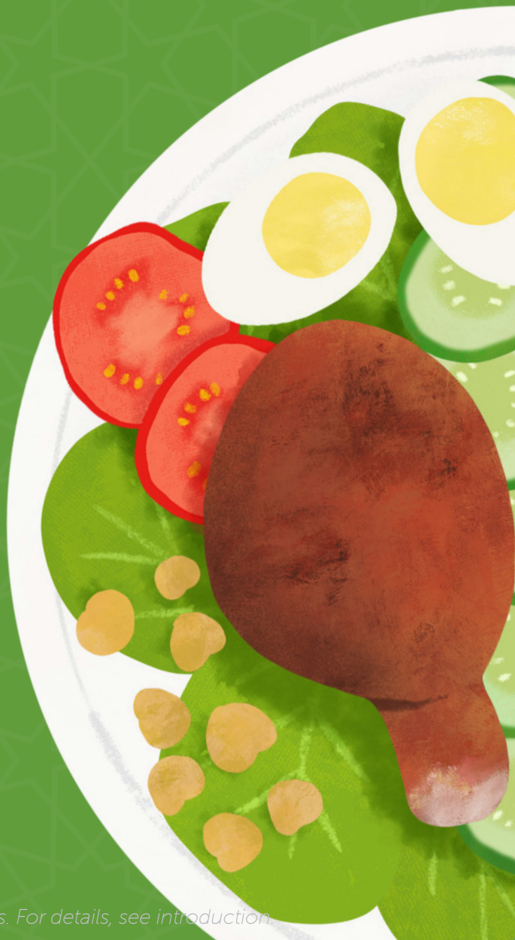

This toolkit is coproduced by Born in Bradford, Bradford Council, and Faith in Communities. For details, see introduction.

HEALTHY DIET WORKSHOP 2

CREATIVE KITCHENS

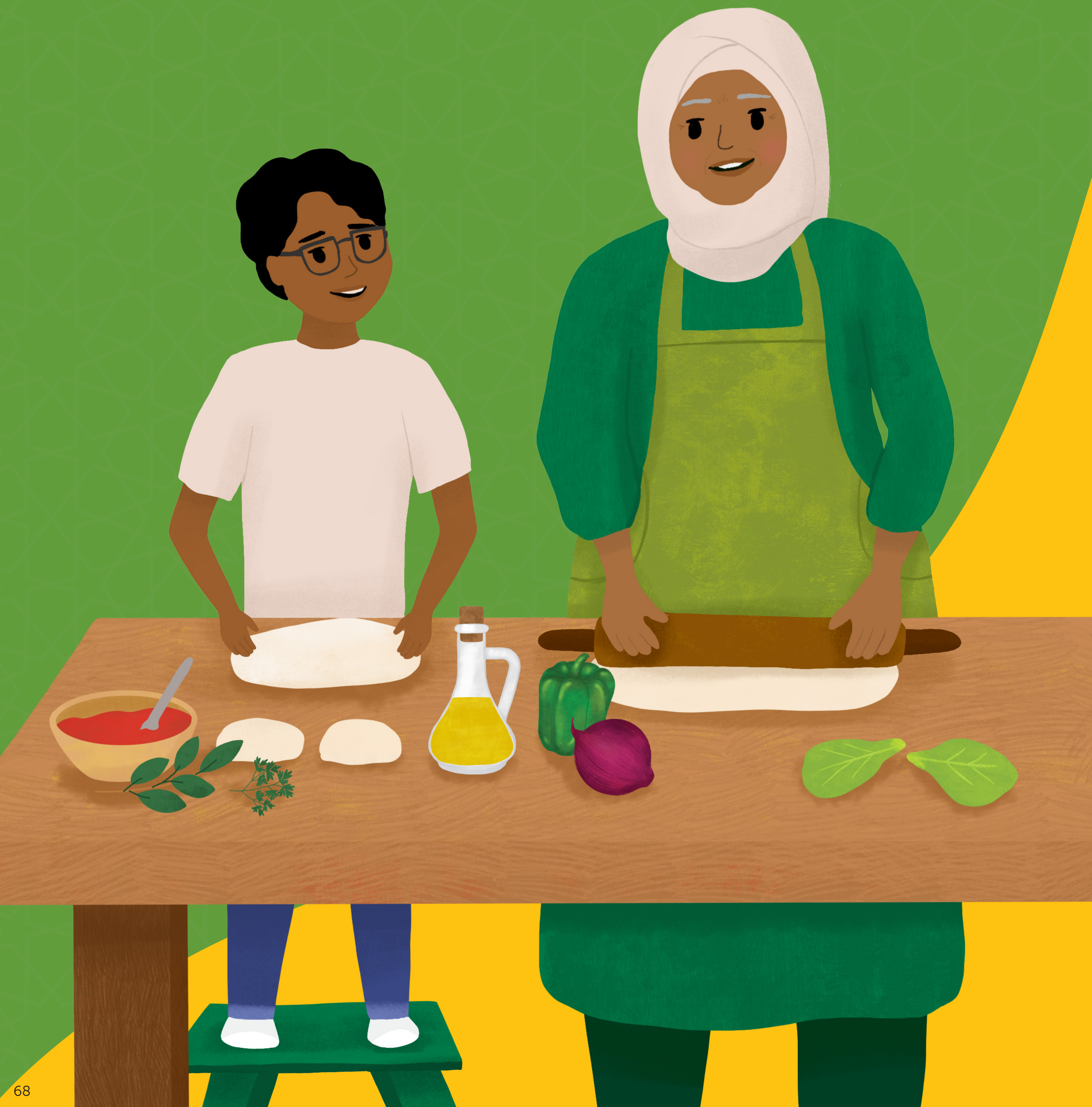

HEALTHY DIET WORKSHOP 2

CREATIVE KITCHENS

AIMS:

- To equip children and families with food preparation skills and healthy cooking techniques
- To develop confidence and creativity in the kitchen

OUTCOMES:

- Children and families will be encouraged to read and follow a recipe
- Children will understand basic food hygiene, kitchen safety and origin of foods

TARGET PARTICIPANTS:

- Children, families, womens groups, and staff in faith settings

LENGTH OF WORKSHOP:

- 1 hour 30 minutes

EQUIPMENT REQUIRED:

Activity 2 - Cooking a Healthy Pizza:

- Oven or grill, chopping board, knife, oven tray
- Recipe ingredients are given in the workshop activities

Activity 3 – Growing a Tomato Plant:

- Children asked to bring in yoghurt tubs, compost bag, approximately 2 tomatoes, water (give children a week's notice)

LOCATION:

- Kitchen in the faith setting or outside the faith setting

HEALTH AND SAFETY:

- Facilitator must hold Food Hygiene Level 2 award
- Facilitator/parental supervision needed at all times when cooking
- Children should be warned to be careful whilst helping in the kitchen with sharp knives or with hot foods

FOOTWEAR AND CLOTHING:

- Encourage children to wear aprons to protect food from contamination
- Roll up long sleeves and wash hands before starting

This toolkit is coproduced by Born in Bradford, Bradford Council, and Faith in Communities. For details, see introduction.

And He (Allah) makes lawful for them pure, wholesome things and He makes unlawful for them noxious things.
[Qur'an, 7:157]

And eat and drink but do not be excessive. Indeed, He does not like those who go to excess.
[Qur'an, 7:31]

The Prophet (pbuh) said, "Eat, drink and spend in charity without going to excess nor ostentation."
[Al-Mustadrak]

Jabir reported, the Messenger of Allah (pbuh) said, "The food of one person is enough for two people, the food of two people is enough for four, and the food of four is enough for eight."
[Muslim]

*City of Bradford Metropolitan District Council, and Born in Bradford do not own the Islamic Narrative in the toolkit and maintain this to be the independent work of Mufti Mohammed Zubair Butt. All enquiries relating to the Islamic Narrative should be referred to Mufti Mohammed Zubair Butt directly. For detailed disclaimer, please see page 2.

ACTIVITY 1
Discussion

15 mins

Facilitator leads a discussion on cooking techniques and recipes.

Facilitator can ask the following questions, and make sure the points given below are covered:

- What are your favourite healthy recipes to cook?
- Which healthy ingredients do these recipes contain?
- Can anyone name cooking methods or techniques that we can use in a kitchen? (E.g. boiling, frying, poaching, grilling, chopping, kneading, mixing, etc.)
- What safety precautions must we take when cooking? (E.g. Wash hands, carry sharp knives or scissors correctly, use a chopping board, clean down the surfaces)
- Where do our ingredients come from?
- What are the benefits of helping in the kitchen and learning to cook?
  - Learning to cook is a life skill that teaches us about nutrition and food safety
  - It can also incorporate maths, literacy, motor skills and teamwork
  - To live a healthy life, we need to know about nutritious foods and how to prepare and cook tasty dishes
  - Adapting a recipe can help to educate children about the health benefits, and social and environmental consequences of healthy eating
  - Cooking with children is quality time spent together and strengthens bonds
  - Cooking can introduce children to scientific concepts, increases focus and attention, boosts confidence and encourages a love for cooking. It's an essential life skill

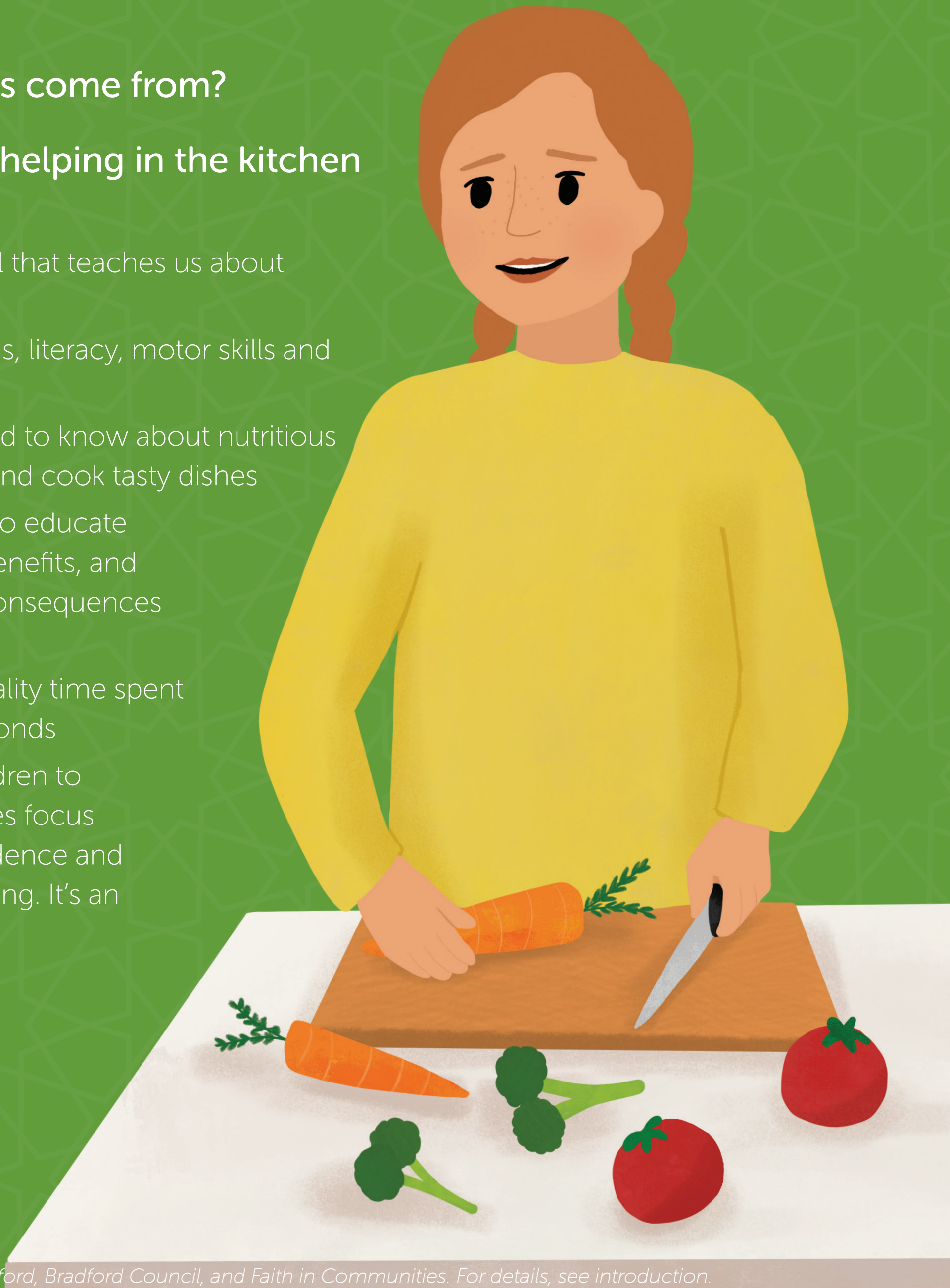

This toolkit is coproduced by Born in Bradford, Bradford Council, and Faith in Communities. For details, see introduction.

ACTIVITY 2
Cooking a Healthy Pizza

40 mins

If children have completed Workshop 1, they can choose to make one of the recipes that they adapted during that workshop, or they can make the healthy pizza recipe below.

If time is short, the facilitator can allow children to make a mini pizza at the faith setting, which they can wrap in foil to cook at home.

Action

1. Facilitator to mention all the healthy ingredient options (e.g. olive oil, mushrooms, onion) that can be used in this recipe.

2. The facilitator leads a brief discussion, asking the children what a healthy pizza would look like compared to an unhealthy pizza. Make sure the discussion covers:

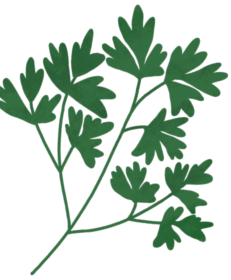
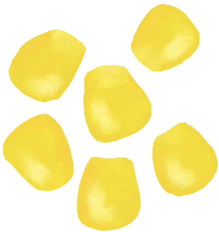
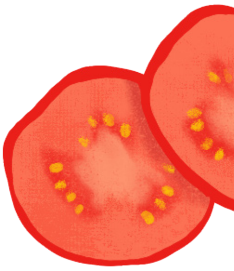
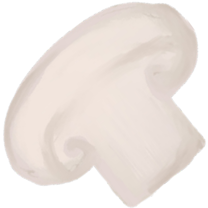

- A healthy pizza doesn't have too much cheese
- No salty meats
- Toppings could be vegetables or chicken
- No sugar in the sauce or dough
- A wholewheat base would be healthier than a plain flour base

3. Children can get creative and make a pizza using all their favourite healthy toppings.

4. Facilitator can explain common words used in different recipes and recap the basic food safety rules below if necessary:

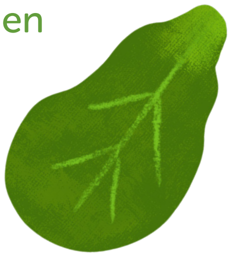

- Parents/carers should always supervise children in the kitchen
- Explain the ground rules of using the kitchen:
  1. Always wash hands first
  2. Tie long hair back
  3. Keep work surfaces clean
  4. Keep raw food such as meats and fish away from cooked or ready to eat foods
  5. Cook food to correct temperatures to ensure it is cooked or heated all the way through
  6. Keep food in the fridge when it is not being used
  7. Don't lick fingers when touching raw foods such as cookie dough, or meats and chicken.
  8. Use chopping boards and wash in between using it for different items
  9. Reheating food: Only reheat food once, this prevents food poisoning. The food should be reheated very hot (over 70°C) to kill any germs
- Make sure to store any leftovers in the fridge

This toolkit is coproduced by Born in Bradford, Bradford Council, and Faith in Communities. For details, see introduction.

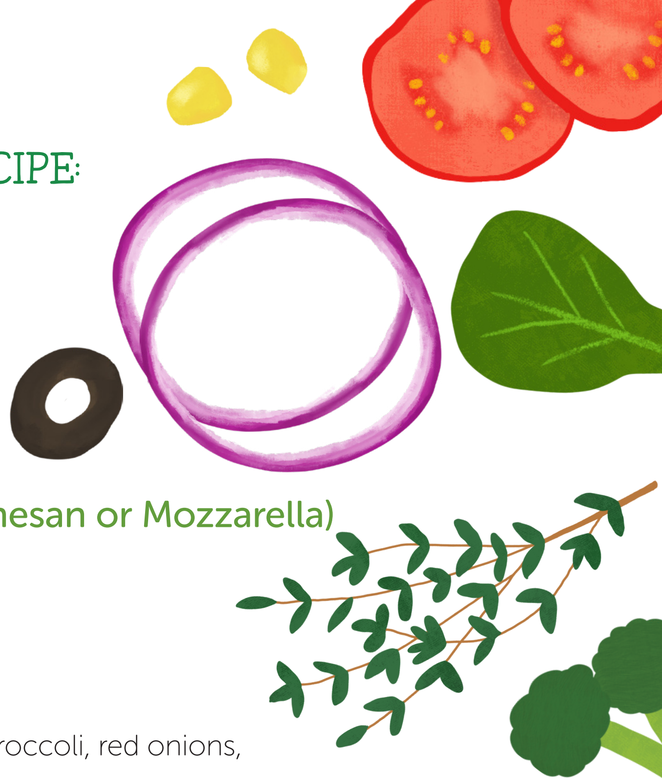

INGREDIENTS FOR HEALTHY PIZZA RECIPE:

Choice of pizza base (1 per child):

- **Chapatti/roti, pitta bread or naan bread**
  Whole wheat if possible, home-made if you like

Toppings:

- **Grated cheese (handful of vegetarian Parmesan or Mozzarella)**
- **Passata/tomato puree**
- **Herbs and spices (try to grow your own!)**
- **Vegetables** E.g. bell peppers, onions, mushrooms, broccoli, red onions, olives, fresh spinach or cherry tomatoes.

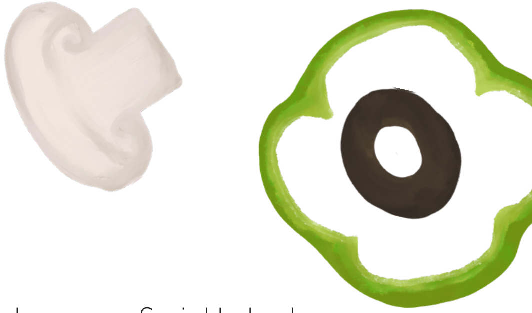

METHOD:

1. Preheat the oven or grill to Gas 6/180°C.
2. Help the children to chop the vegetables and cooked chicken.
3. Spread the passata or tomato puree onto the chapatti, pitta bread or naan. Sprinkle herbs or spices on top and place the pizza base onto an oven tray.
4. Get creative and top with a choice of vegetables. Choose as many different colours as you can!
5. Sprinkle the grated cheese on top and place in the oven or grill for 6-8 minutes, until the cheese is golden brown. Serve and enjoy!

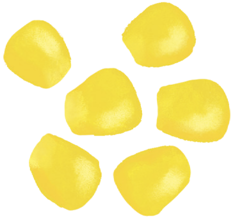

ACTIVITY 3
Growing a Tomato Plant

20 mins

When children learn where their food comes from, they are more likely to try new flavours and textures.

There is nothing like growing and eating their own produce to give children an appreciation of how plants need to be nurtured with earth, warmth, and water, in order to feed us.

Growing fruit and vegetables will develop a caring and appreciative nature in children, as well as patience for when harvest time comes. Growing food that you can eat straight from the plant, such as strawberries, tomatoes and peas is always a hit with children.

This toolkit is coproduced by Born in Bradford, Bradford Council, and Faith in Communities. For details, see introduction.

Instructions

This activity must be done in March or April, and can also be done with dried peas or strawberries, as they are quick to grow.

Having no garden doesn't mean you can't grow anything. You just need a window sill.

1. Facilitator to cut up some ripe tomatoes and carefully take out the seeds so children can see.
2. Separate seeds so that each child has approximately 3-5 seeds. There are usually around 90 seeds in one tomato.
3. Children should place a little soil into their named yoghurt pot, put seeds in and cover with ¼ inch soil.
4. Sprinkle with water and place by a sunny window.
5. Ask the child to water a little every week, either at the faith setting or at home.
6. In 4-6 weeks the plant will be ready to plant outside or into a larger pot, and should be watered daily. Tomatoes will be ready to eat after about 2 months.

If the faith setting would like to take children to visit a farm, some farms local to Bradford are:

- Swithens Farm and Play Barn, Leeds. *(swithensfarm.co.uk)*
- Harewood House, Leeds. *(harewood.org)*
- Meanwood Valley Urban Farm, Leeds. *(mvuf.org.uk)*
- Hesketh Farm Park, Skipton. *(heskethfarmpark.co.uk)*

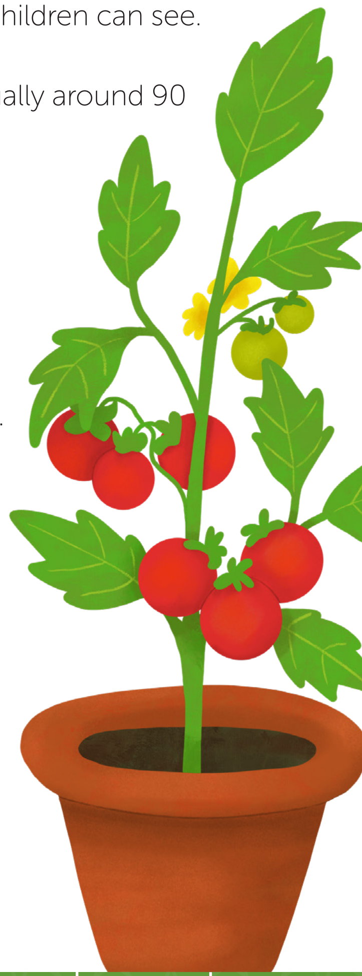

ACTIVITY 4
Discussion

15 mins

Facilitators use this time to have an open-ended discussion with children about what they think may be the barriers to them or others cooking healthy foods.

What helpful tips can they give one another? Some barriers might be:

- Cooking skills
- Facilities to cook
- Space to store healthy food
- Time to cook and parents' time
- Cost of healthy foods
- Availability of cheap takeaway foods
- Personal taste, or picky eaters in their families
- Cultural and family traditions
- Peer pressure

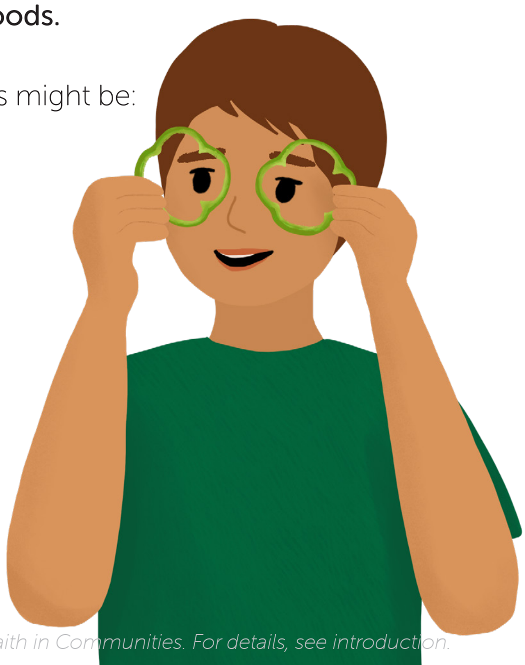

This toolkit is coproduced by Born in Bradford, Bradford Council, and Faith in Communities. For details, see introduction.

HEALTHY DIET WORKSHOP 3

DRINKING WATER

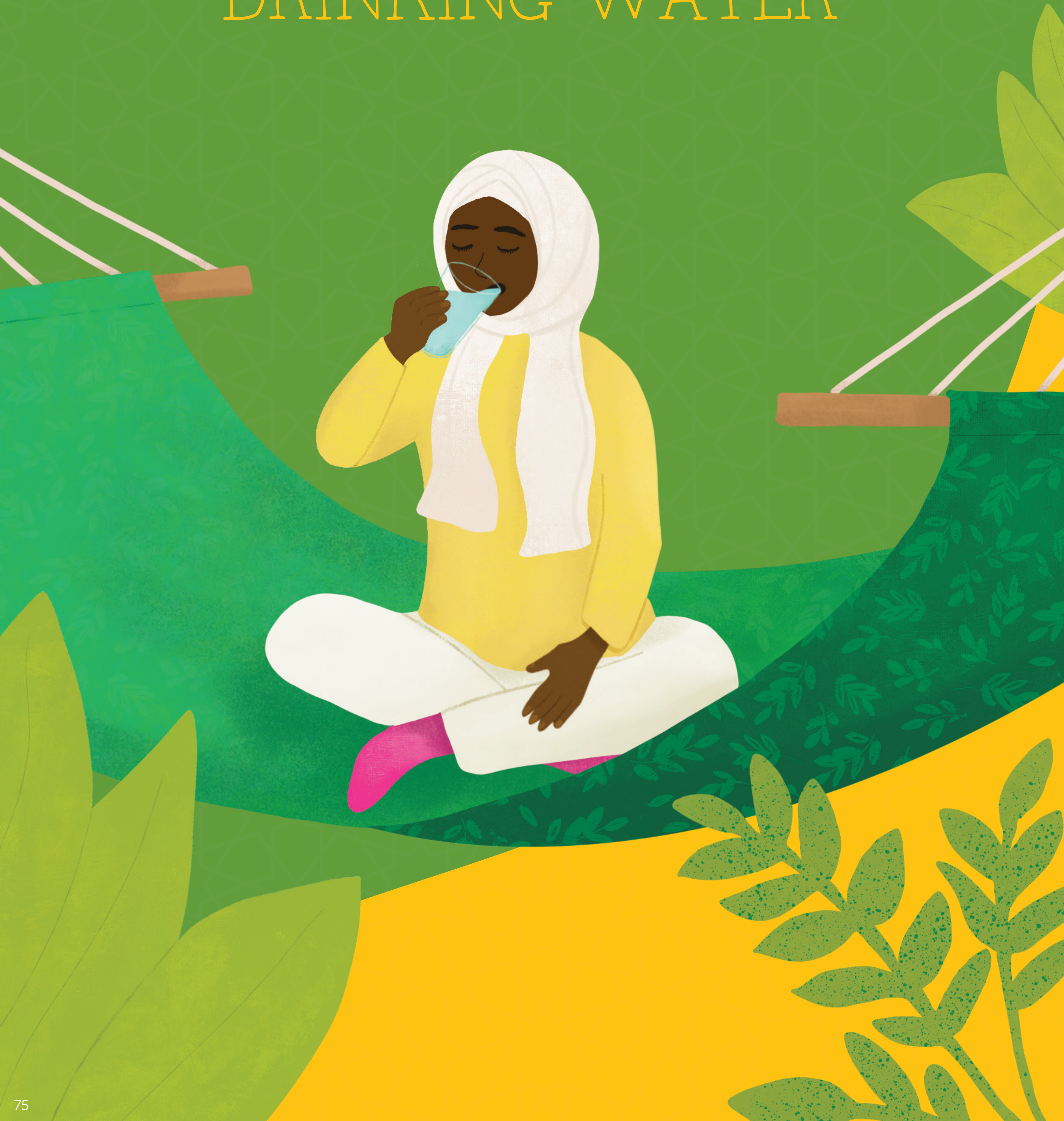

HEALTHY DIET WORKSHOP 3

DRINKING WATER

AIMS:

- To encourage families to drink more water, and understand the importance of doing so

OUTCOMES:

- Families drink more water in place of fizzy/sugary drinks

TARGET PARTICIPANTS:

- Children, families, and staff in faith settings

LENGTH OF WORKSHOP:

- 50 minutes

EQUIPMENT REQUIRED:

Activity 2 - Making Flavoured Water:

- Sharpie pens
- Illustrations (provided at end of workshop)
- A selection of fruits to flavour water with, such as berries (strawberry, blueberries, raspberry), lemon, cucumber, mint, dates (1 per child). (You may want to involve children in choosing flavours). Ensure all fresh fruit is washed and suitable for consumption
- Recycled plastic bottles

Activity 3 – Keeping a Water Diary:

- Paper/notebook

Activity 4 – Investigating Sugar Quantities in Drinks:

- Kitchen scales
- Sugar
- Clear plastic cups (or preferably recycled bottles cut in half)
- Teaspoon

LOCATION:

- Classroom with access to drinking water tap

HEALTH AND SAFETY:

- Facilitator must hold Food Hygiene Level 2 award
- Young children to be given help cutting fruit to put in water bottles

FOOTWEAR AND CLOTHING:

- N/A

ISLAMIC NARRATIVE

The Arabic word for water is mā’ and is mentioned 63 times in the Holy Qur’an.

Water is the source of all life.
“... and We made from water every living thing.”
[Qur’an: 21:30]

In explaining the verse above, Qatadah said,
“Every living thing was created from water.”
[Tafsir al-Tabari]

Ibn Kathir said, “The origin of every living thing is in water.”
[Tafsir Ibn Kathir]

Prophet Isa (Jesus) AS used to advise the children of Israel to drink pure water.
[Muwatta’ Imam Ma-lik]

Please share:

- The story of Zam Zam (well)
- The story of the well of Uthman

*City of Bradford Metropolitan District Council, and Born in Bradford do not own the Islamic Narrative in the toolkit and maintain this to be the independent work of Mufti Mohammed Zubair Butt. All enquiries relating to the Islamic Narrative should be referred to Mufti Mohammed Zubair Butt directly. For detailed disclaimer, please see page 2.

ACTIVITY 1
Discussion

15 mins

What are the benefits of drinking water?

Facilitator to ask children if they know the benefits of drinking water.

Ensure the following points are covered:

- Water is very important for our body to function properly. It helps with the digestion of food, regulates our body temperature and prevents constipation. It is needed for a healthy brain and liver
- Children's bodies are 75% water
- Keeping hydrated improves the condition of our skin and hair
- Keeping hydrated can improve concentration levels to help children learn, helping brain power and energy
- Children need about 6-8 cups of water per day
- Having high sugar drinks (e.g. Coke, Pepsi, etc.) can cause tooth decay
- Water is sugar free, making it the best alternative. In Activity 3 we will explore sugar content in drinks

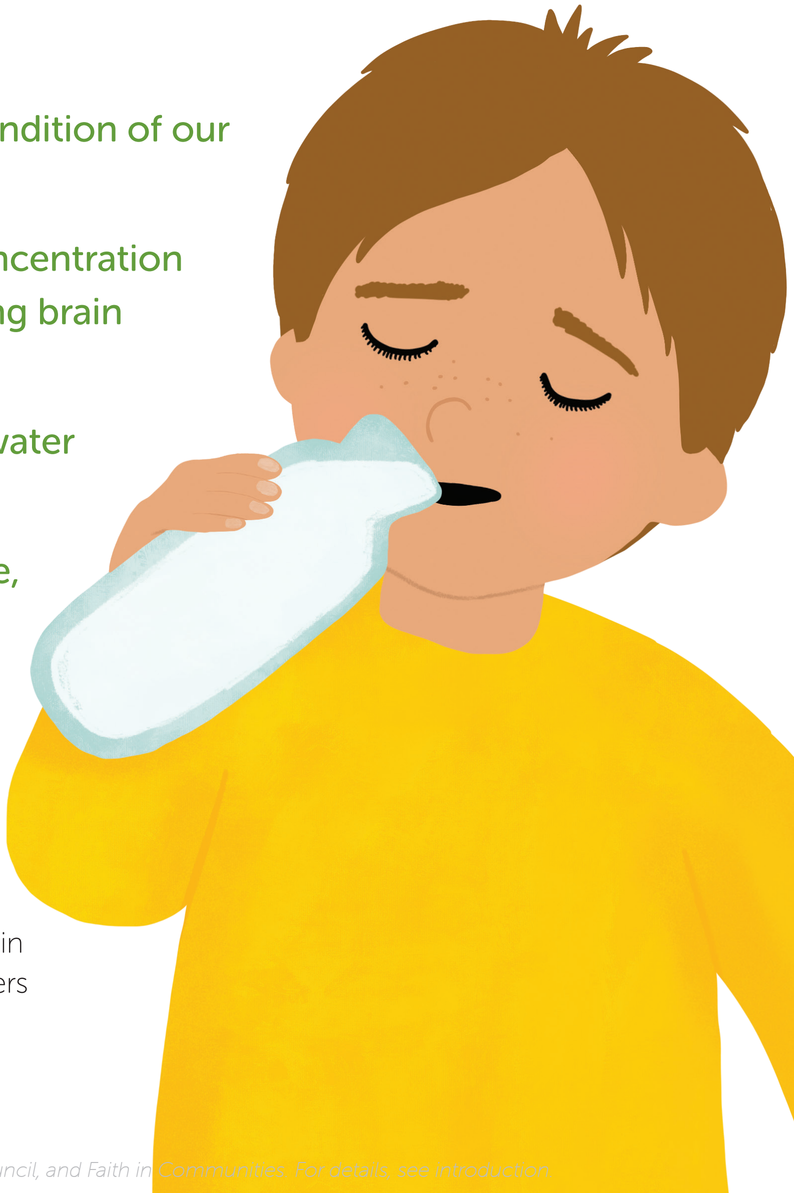

The posters overleaf can be printed and displayed in the faith setting for regular and prominent reminders on the benefits of drinking water.

PRINT & CUT

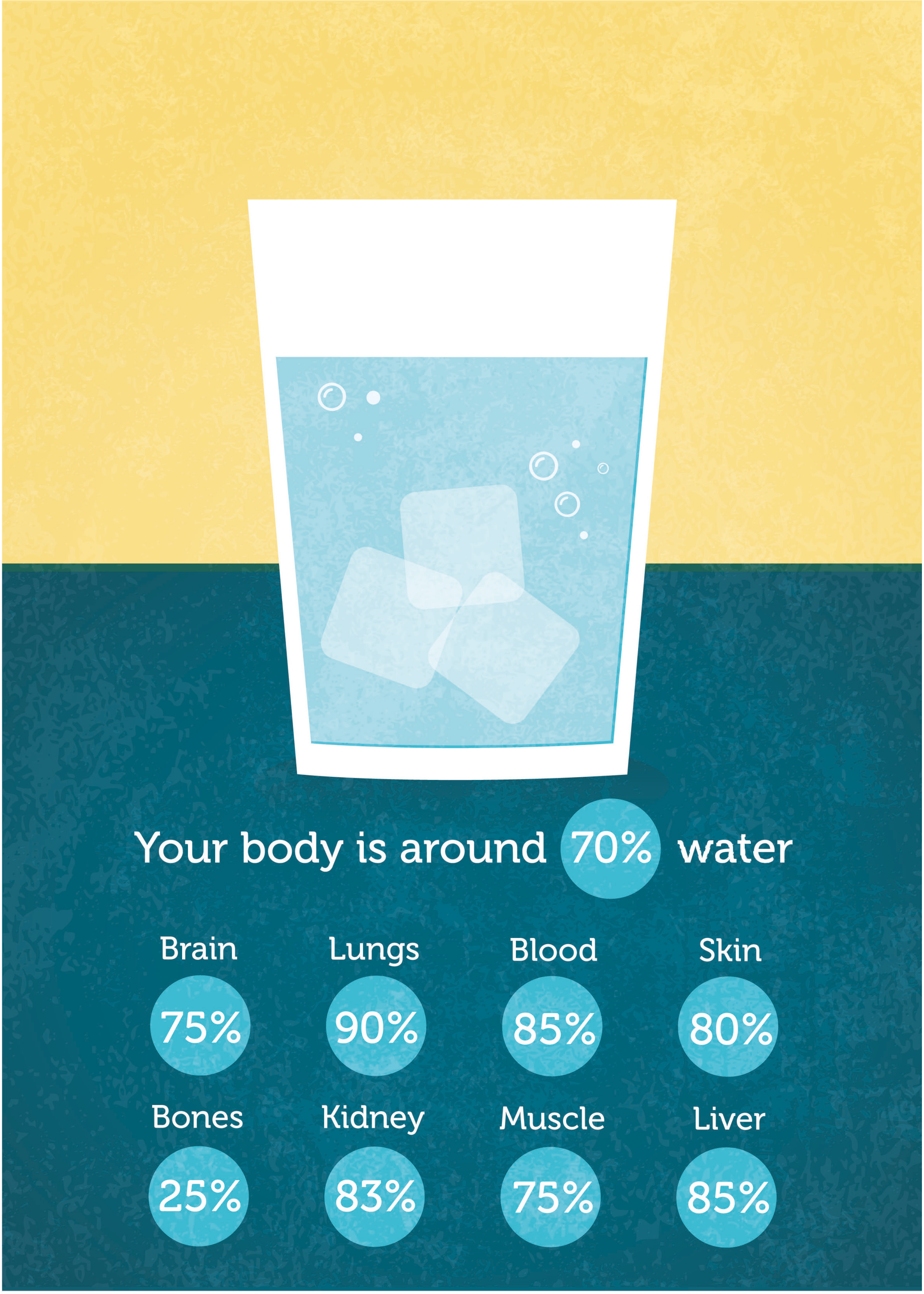

This toolkit is coproduced by Born in Bradford, Bradford Council, and Faith in Communities. For details, see introduction.

PRINT & CUT

Water is not only essential for quenching your thirst, but it also has superpowers that can make you feel great!

Super Hydration Power: Just like plants need water to grow, your body needs water to stay hydrated. Water helps you feel refreshed, keeps your body cool, and makes you feel energised to play and learn all day long!

Brain Boosting Magic: Water is your brain's secret weapon! When you drink water, it helps your brain work at its best, making it easier to remember things, concentrate, and think creatively.

Strength and Stamina: Water is like a secret potion for your muscles. When you're running, jumping, or playing sports, water keeps your muscles strong and flexible.

Happy Tummy: When you drink water, it helps your tummy stay healthy and happy. It keeps away tummy troubles, and helps things to move smoothly, just like a river!

Superhero Skin Shield: Your skin is like a protective shield that covers your whole body, and drinking lots of water helps your skin to glow. It also makes your skin soft and smooth, and even helps prevent spots!

Energy to Explore: If you feel tired and low on energy, water can be your very own magic potion! When you drink water, it gives you a boost of energy, like a superhero power-up.

Mouth-Care Marvel: Your teeth and gums need some love too! Water helps keep them strong and healthy and gives them a super shield against sugar bugs!

It's important to drink water every day. While other drinks can be tasty, nothing beats the power of water. So, keep a water bottle handy and take sips during the day to stay hydrated and enjoy all the amazing benefits!

This toolkit is coproduced by Born in Bradford, Bradford Council, and Faith in Communities. For details, see introduction.

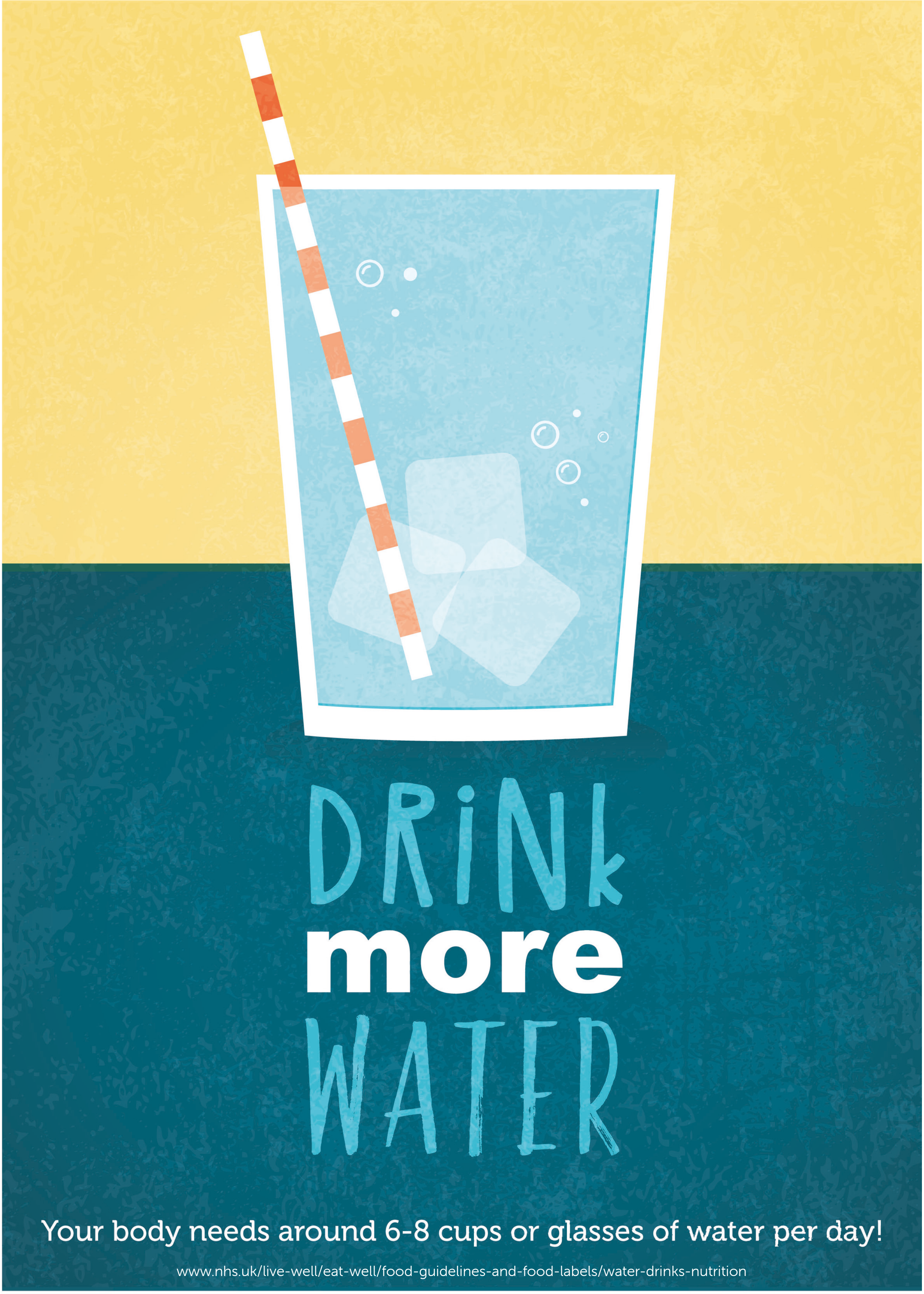
DRINK
more
WATER
Your body needs around 6-8 cups or glasses of water per day!
www.nhs.uk/live-well/eat-well/food-guidelines-and-food-labels/water-drinks-nutrition

ACTIVITY 2
Making Flavoured Water

10 mins

This activity allows children to practise making fruit-flavoured water, which can have more flavour than tap water and is a healthier alternative to fizzy drinks. (This can be done whilst at the faith setting or given as instructions for children to try at home).

Instructions

1. Using empty water bottles (remove all labels) ask the children to write their own name in large English or Arabic letters across the bottle, with a Sharpie pen.
2. Encourage children to decide what flavour they want to use in their water and demonstrate how to add fruit to their bottles (assist younger children if required).
3. Assist children with filling their bottle with water (you may want to consider half filling to avoid wastage). The water is then ready to drink or take home and freeze for use another day.
4. If possible, use water bottles with a visual 'timer' which supports children to know when and how much water they should drink in a day, from the bottle.

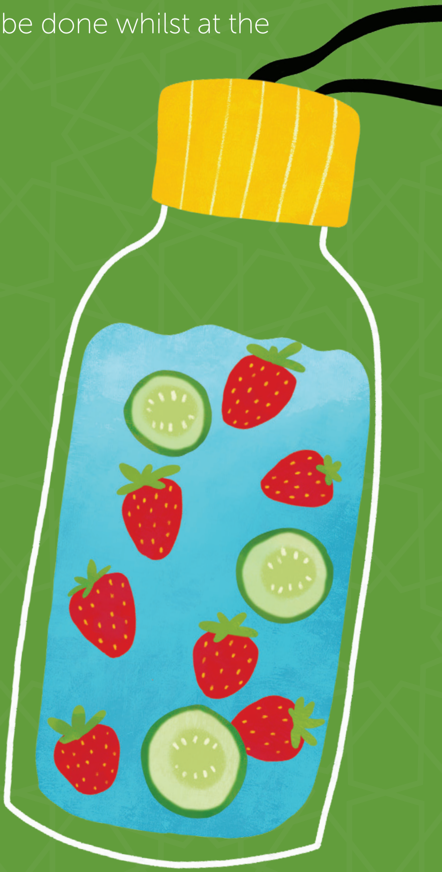

ACTIVITY 3
Keeping a Water Diary

5 mins

Children should be encouraged to keep a water diary to monitor their water intake.

This should be set as homework for one or two weeks.

Action

1. Facilitator to ask children to keep a record of how many glasses of water they drink each day for one week.
2. Children should write down any interesting things they did (e.g. add fruit flavours to water).

- If feasible, the faith setting can use this activity as a competition and offer rewards for achievements, or it can be used as a record.

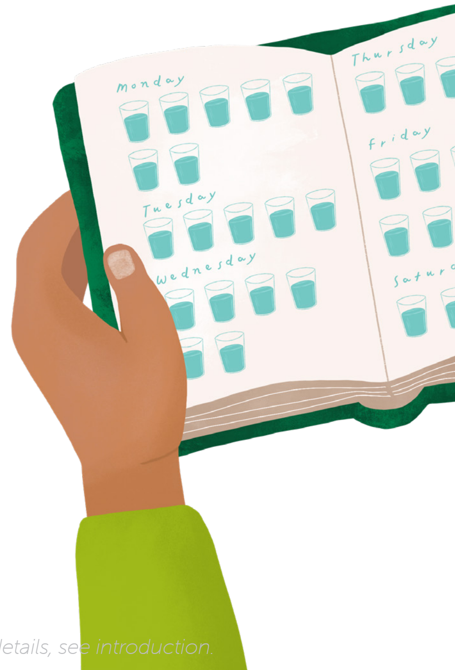

ACTIVITY 4

Investigating Sugar Quantities in Drinks

20 mins

The amount of sugar in children's favourite drinks can often go unnoticed. This activity will visually show children the amount of sugar in popular soft drinks.

For facilitators use: Below is a table of popular drinks and the conversions from grams to teaspoons of sugar. *Children should have no more than 6 teaspoons (24g) of added sugar in all their food and drink throughout the day*

Popular Drink	Grams of Sugar	Teaspoons of Sugar
Can of Coke (330ml)	35g	9
Can of Diet Coke (330ml)	0g<br>• Discuss sweeteners - artificial chemicals that make drinks sweet without adding any sugar<br>• High or regular artificial sweeteners consumption is not good for health	0
Red Bull energy drink (250ml)	27.5g	7
Fresh orange juice (300ml)	26g. 150ml = 1 of 5 a day<br>• Talk to children about natural sugars found in fruit juices, we can drink 150ml of fresh fruit juice each day	6.5
Chocolate milkshake (400ml)	35g	9
Ribena (500ml)	21g	5
Oasis Summer Fruits (500ml)	20g	5
Bottle of water (500ml)	0g<br>• Always try to choose water - it's the best option	0

This toolkit is coproduced by Born in Bradford, Bradford Council, and Faith in Communities. For details, see introduction.

Facilitator provides a range of drinks that children usually consume (e.g. fizzy drinks, milkshake, energy drinks, Ribena, Fruit Shoot, etc.) and lays them out on a display table.

Empty clear plastic cups, recycled plastic bottles or small glass cups can be used for this activity.

Instructions

1. Facilitator places the chosen clear container or cup in front of each drink.
2. Children count teaspoons of sugar into the cups to estimate the amount of sugar in each drink.
3. Children then count teaspoons and calculate how close they were to the actual measurement on the drink (converted in grams).
4. Facilitator can compare the differences between estimates and measurements.

Optional: create an exhibit with weighed out sugar and corresponding drink packaging to display in the madrasa for other children and parents, as below:

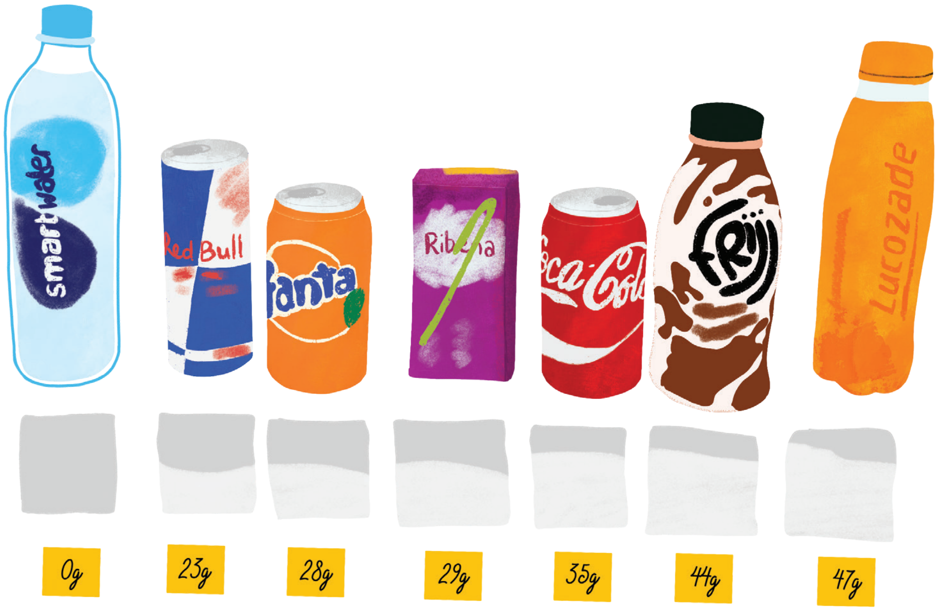

Helpful Tips (Note for faith setting- reinforce these messages as a summary):

- **Encourage yourself and your friends and family to drink more water by carrying a water bottle wherever it is possible to do so**
- **Add a little fruit flavour to water, to make it more interesting**
- **If you like fizz, try sparkling water instead**
- **Further reading on artificial sweeteners and risk of cardiovascular diseases is available at bit.ly/3N8EzLX**

HEALTHY DIET WORKSHOP 4

MEALTIMES AND MANNERS

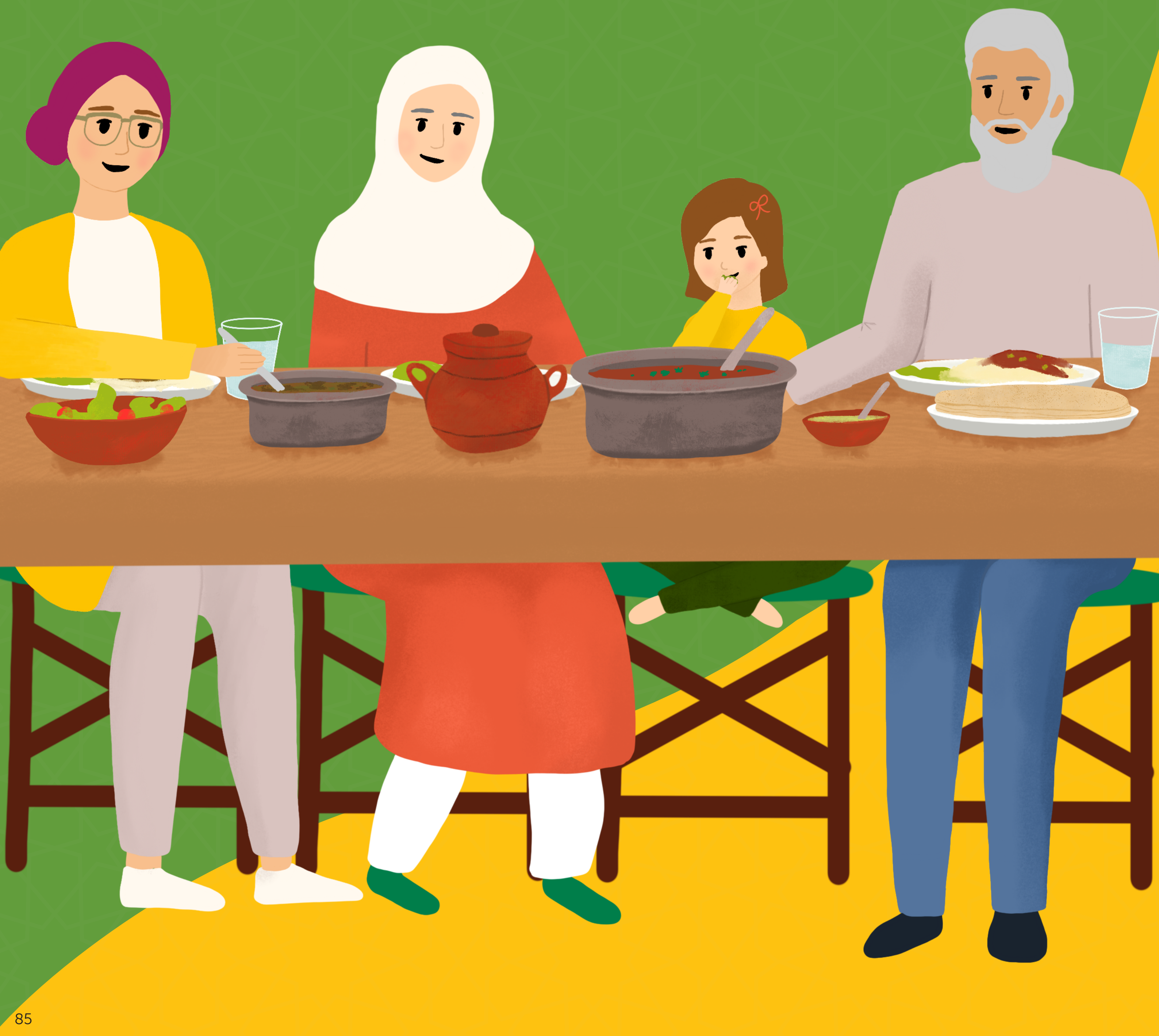

HEALTHY DIET WORKSHOP 4

MEALTIMES AND MANNERS

AIMS:

- Teach children about good manners for eating
- Children and families learn the importance of mealtimes together

OUTCOMES:

- Families make effort to have mealtimes together whenever possible
- Children take learning home and adopt good manners when eating all meals

TARGET PARTICIPANTS:

- Children and families

LENGTH OF WORKSHOP:

- 1 hour

EQUIPMENT REQUIRED:

Activity 2 - Mealtime Manners Poster:

- Colouring pens and blank A4 paper

LOCATION:

- Classroom and at home

HEALTH AND SAFETY:

- N/A

FOOTWEAR AND CLOTHING:

- N/A

This toolkit is coproduced by Born in Bradford, Bradford Council, and Faith in Communities. For details, see introduction.

And eat and drink but do not be excessive. Indeed, He does not like those who go to excess.
[Qur'an, 7:31]

Miqdam ibn Madikarib reports that he heard the Messenger of Allah (pbuh) saying, "No human ever filled a vessel worse than a stomach. Sufficient for any son of Adam are morsels that keep his back straight. But if it must be, then a third for his food, a third for his drink and a third for his breath.
[Al-Tirmidhi]

Abu Saeed Al-Khudri reports, "The Messenger of Allah (pbuh) prohibited drinking from the broken portion of the vessel and from blowing into the drink."
[Abu Dawud]

*City of Bradford Metropolitan District Council, and Born in Bradford do not own the Islamic Narrative in the toolkit and maintain this to be the independent work of Mufti Mohammed Zubair Butt. All enquiries relating to the Islamic Narrative should be referred to Mufti Mohammed Zubair Butt directly. For detailed disclaimer, please see page 2.

Abu Huraira reported, "The Prophet (pbuh) never found fault in any food. If he desired it, he ate it, otherwise he left it."
[Al-Bukhari]

The Messenger of Allah (pbuh) said, "I do not eat whilst I am reclining."
[Al-Bukhari]

Ibn Abbaas reported that the Prophet (pbuh) said, "The blessing descends in the middle of the food, so eat from the two edges of it and do not eat from the middle of it."
[Al-Tirmidhi]

This toolkit is coproduced by Born in Bradford, Bradford Council, and Faith in Communities. For details, see introduction.

ACTIVITY 1
Discussion

30 mins

Showing good manners and eating meals together as a family can help us eat in a healthy way.

This workshop focuses on how children can practise good manners during family mealtimes.

Action

1. Children split into two groups. One group starts by discussing good manners, and the second group starts by discussing the importance of eating meals together as a family.
2. Facilitator leads discussion, sharing information from the points below.
3. Facilitator invites children to contribute how they can ensure this is practised during all meals.
4. After 15 minutes, groups swap over and discuss the other topic.

The facilitator should try to cover the following discussion points:

Good Manners Group Discussion:

Always:

- **Eat modestly:** The maximum capacity we should eat is ⅓ of our stomachs for food and ⅓ for water. The Prophet (pbuh) said the worst vessel man could fill is his stomach, so we should be careful that we do not stuff ourselves.
- **Do not hurry your food, eat slowly:** Eating slowly helps you to enjoy and savour your food and often eat less, because eating slowly gives the brain time to register when you are full.
- **Do not blow on your food, allow it to cool down slowly:** Today we realise how germs can be spread by our breath. If we were sharing a drink or eating together from one plate, then it would not be a good idea to blow on food.
- **Sharing food with others:** Sharing food creates bonds. Cooking with family or friends can help strengthen these relationships by encouraging a sense of trust, community, belonging, and closeness. It brings people together as they are automatically involved in a sharing process. This social activity has a cumulative effect – it can save money, build a deeper appreciation for the foods that provide energy, and ultimately meet the basic human need of connection with others. It can raise self-esteem and confidence. Sharing the food itself becomes the connecting factor between each individual present. Young and old, rich and poor, black and white, scholar or layman: sharing the same food from the same source somehow renders everyone present equal for that moment in time.

This toolkit is coproduced by Born in Bradford, Bradford Council, and Faith in Communities. For details, see introduction.

- **Do not criticise your food - if you don't like it, just leave it:** Doing this is being respectful to the person who cooked it and also to Allah, who ultimately provided it. Learning not to criticise food will help a child to be more thankful for what they have. We should also think of children and others who have no food to eat. They would be so grateful for anything, even if it was just dry bread.

To ensure good manners, it is important that you:

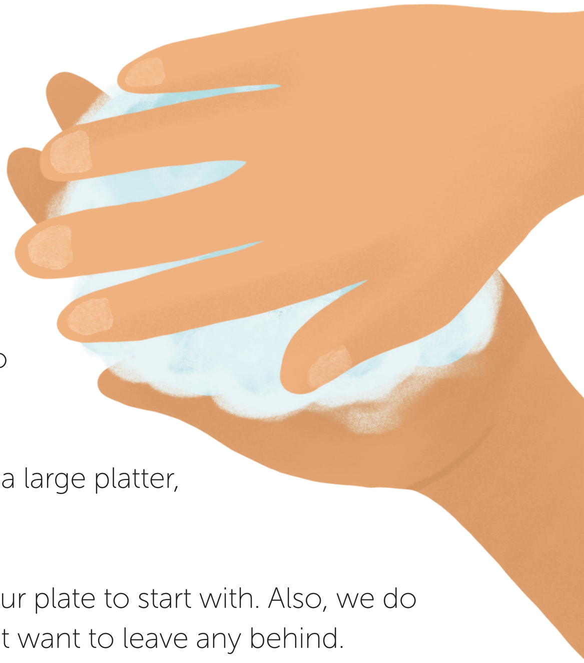

1. Wash your hands.
2. Sit up straight, do not lean back or eat whilst lying down.
3. Sitting on the ground with your knees bent or legs folded is an Islamic practice. This is an act of humbleness.
4. Say the prayer 'Bismillah' and start your meal. If you forget to do so at the beginning, say it whenever you do remember.
5. Eat from the side closest to you. Even if you are not sharing a large platter, always eat from the side closest to you.
6. Wasting food is not good. Do not put too much food on your plate to start with. Also, we do not know where on your plate the blessings are, so we don't want to leave any behind.
7. Be grateful for the food you eat and say thanks to whoever made the meal for you.

Eating Meals Together Group Discussion:

Facilitator discusses with children the benefits and barriers of having mealtimes together, discussing and sharing some of the key benefits. Facilitator can select the most relevant of the following points for discussion.

Benefits:

- **Earn blessings:** Sharing meals with your family, helping to cook, serving others, washing up, and giving them water at the table, all earn you many blessings and will make others happy.
- **Feel good factor:** When you eat, relaxing and happy hormones ('endorphins') are released.
- **Positive role models:** Family mealtimes offer the adults time to be role models in healthy eating, politeness, and Sunnah table manners, and encourage children to eat a variety of foods (depending on availability of foods).
- **Strengthen family bonds:** Eating together creates bonds and gives you time to talk about your day. This fosters a sense of belonging, leading to greater self-esteem. When you are talking, you spend longer eating and you tend to eat slower, which makes you eat less and feel fuller. However, note that it is generally considered rude to talk with your mouth full, and talking should only happen between mouthfuls.

This toolkit is coproduced by Born in Bradford, Bradford Council, and Faith in Communities. For details, see introduction.

- **Healthier diet:** A Harvard study found that families that eat together are twice as likely to eat their five servings of fruit and vegetables than those who do not eat together.

Overcoming barriers:

- What do the children think may be barriers to them or others, sitting down together at mealtimes, and what helpful tips can they give one another to overcome these?
- Discuss social norms which discourage a family eating together at mealtimes, e.g. eating in front of the TV, or buying fast food from a drive-through and eating in the car
- Discuss how eating together at mealtimes and helping to cook can be beneficial for your mental and physical health, helping you to bond with loved ones. This is just as important for boys to do, as well as girls
- Discuss how mealtimes can be a social experience, getting up to date with each others' daily lives
- Talk about the Sunnah of sitting on the floor and eating. Do any of the children do this? If not, they could try it with their family

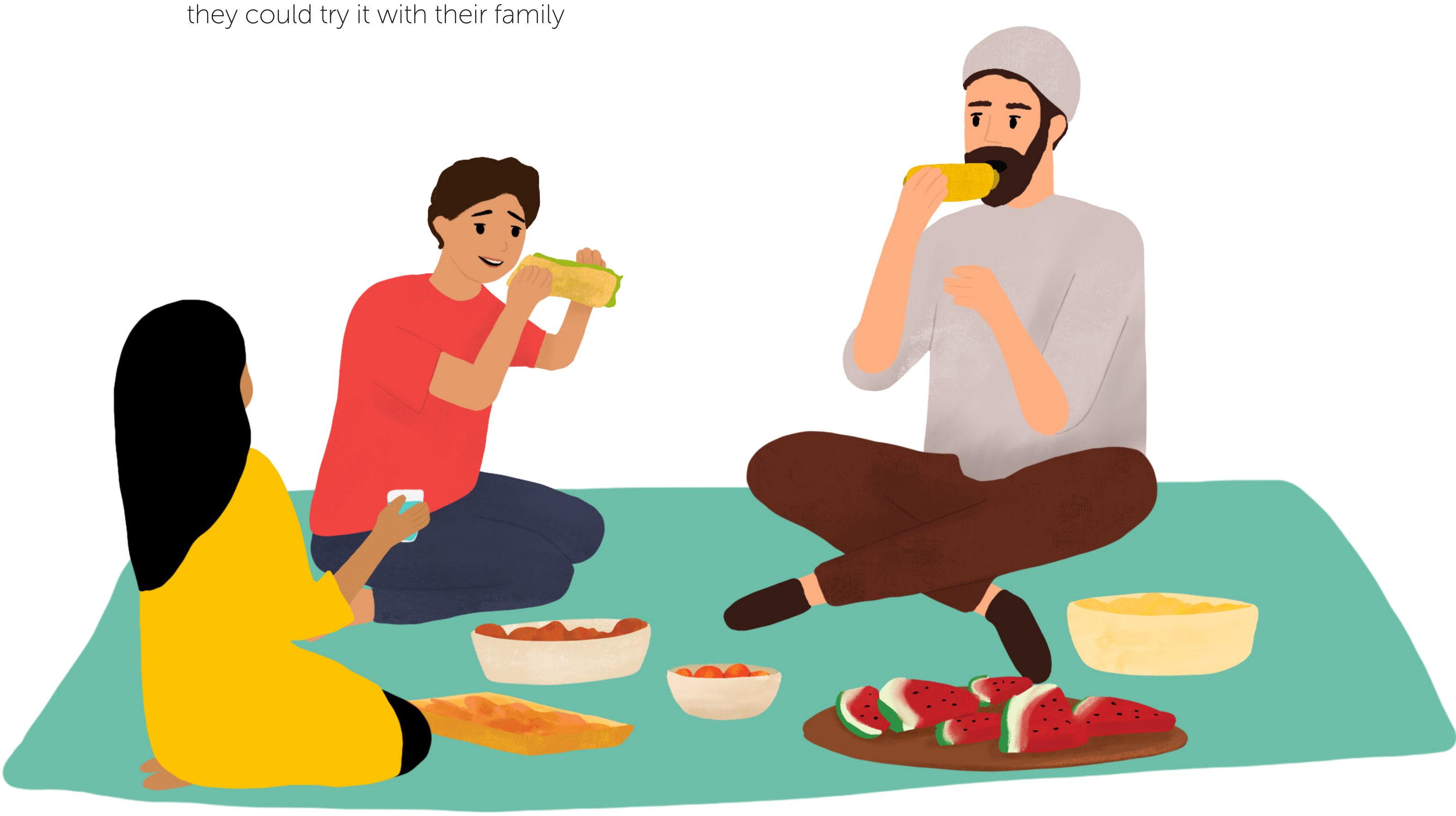

ACTIVITY 2
Mealtime Manners Poster

25 mins

Using A4 blank paper and pens, children have 20 minutes to create a poster containing important rules and practices for mealtimes, that they have just discussed in their groups.

These should include the reasons why we share mealtimes together, and also important manners that they would like to practise at home.

This toolkit is coproduced by Born in Bradford, Bradford Council, and Faith in Communities. For details, see introduction.

ACTIVITY 3

Homework: Reflective Diary

5 mins

After creating a poster, the facilitator sets a homework task to create a reflective diary.

Action

1. Ask the children to help their parents or family members in the kitchen that weekend. They can help to mix, chop, clear away and serve food and water.
2. Facilitator should encourage children to take the initiative at home and get everyone to sit and eat together (this may have to be on a specific day if parents work).
3. Children must record how they followed the rules set on their posters.
4. Diary reflections can be discussed in madrasa the following week. Teachers should encourage children to be honest when writing in their reflective diary.

? Follow-up questions to ask:

- Who helped with the preparation of the meal?
- Did you serve anyone?
- Did you say ‘Bismillah’ or read the eating Dua?
- Were mum and dad thanked for the food?
- Did you help clear up or wash the dishes?
- How did it feel to help?
- How did your parents react? What did they say and how did that make you feel? *Maybe ask mum or dad to write a comment here.*
- What did you talk about whilst eating?
- Did it feel good to eat together?
- Would you do this again?

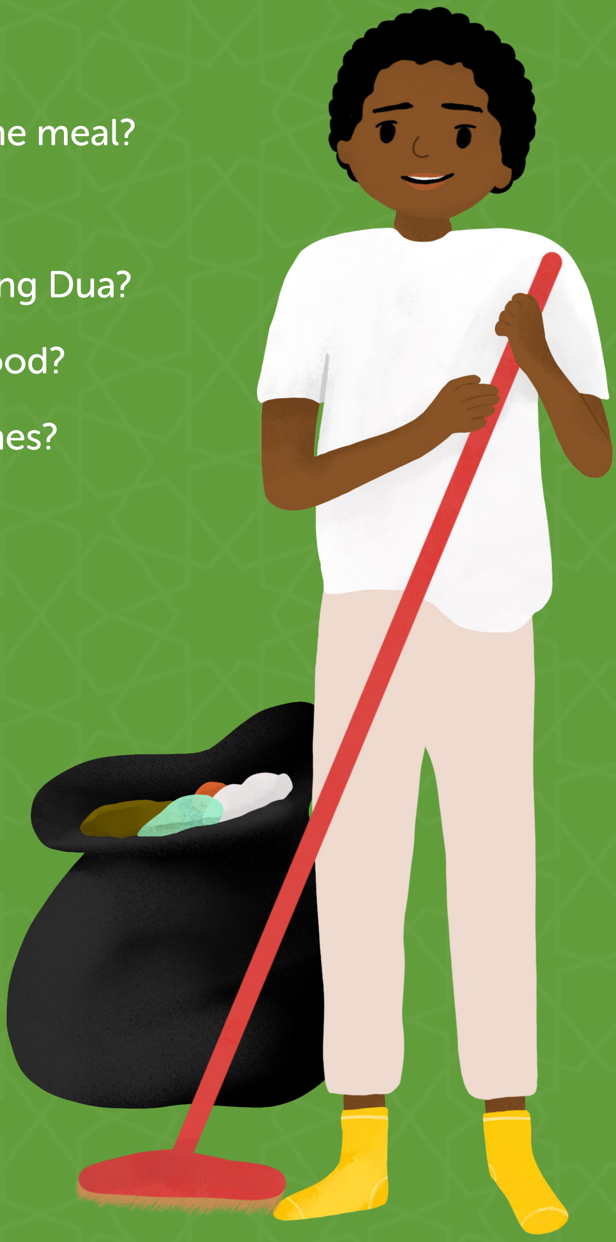

We are family BORNINBRADFORD | JU:MP | FAITH IN COMMUNITIES | LIVING WELL | City of BRADFORD METROPOLITAN DISTRICT COUNCIL

Department of Health & Social Care | BRADFORD INSTITUTE FOR HEALTH RESEARCH MAKING RESEARCH REAL | SPORT ENGLAND | Local Government Association

HEALTHY DIET WORKSHOP 5

PORTION SIZE

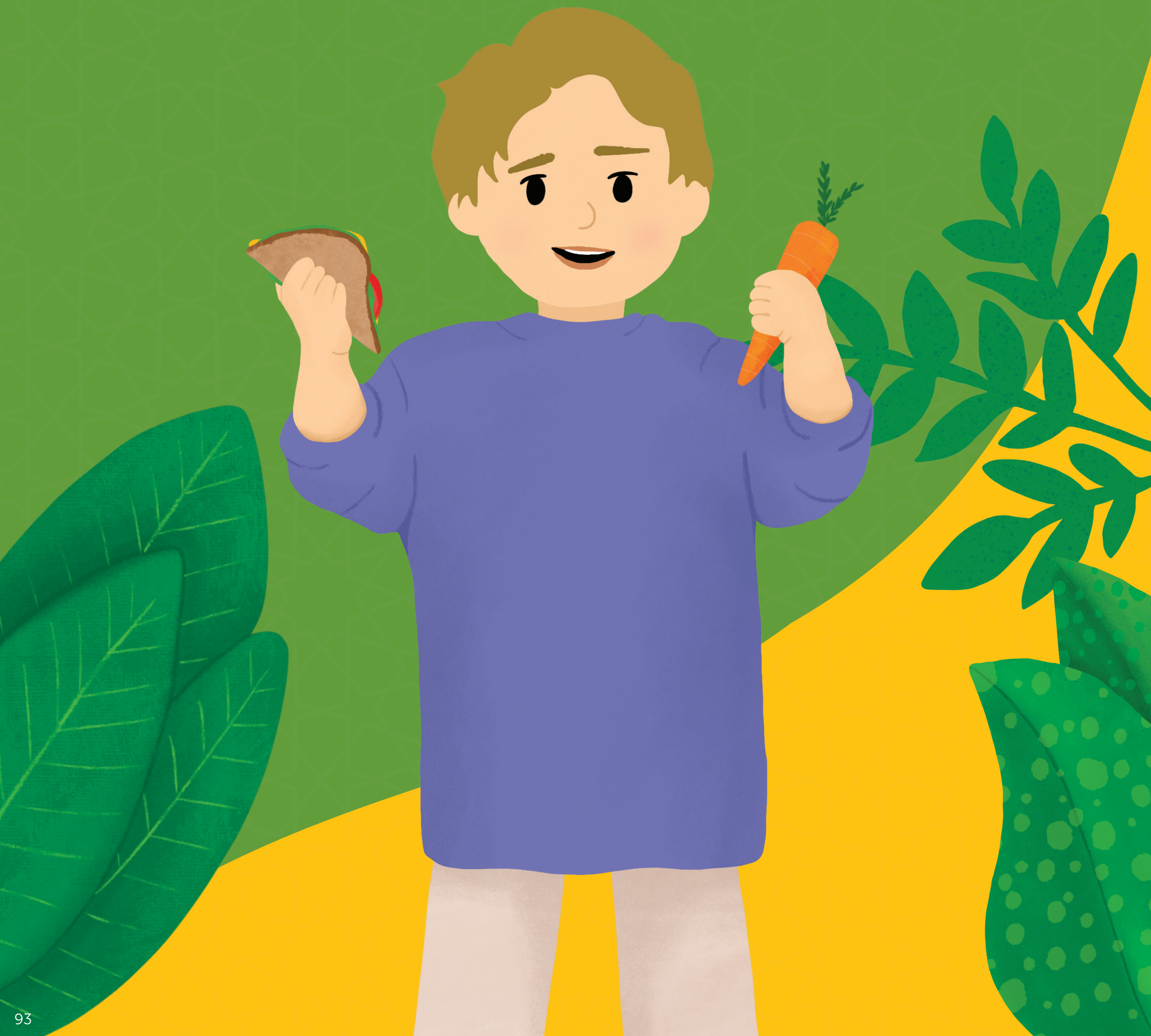

HEALTHY DIET WORKSHOP 5

PORTION SIZE

AIMS:

- Children understand the importance of portion size
- Children understand what appropriate portion sizes look like for different foods

OUTCOMES:

- Children adopt healthy portion size and encourage their families to do the same

TARGET PARTICIPANTS:

- Children

LENGTH OF WORKSHOP:

- 30 minutes in class

EQUIPMENT REQUIRED:

Activity 2 - Personalised Portion Sizes:

- A4 paper
- Different coloured pens
- Handouts on portion sizes

LOCATION:

- Classroom and at home

HEALTH AND SAFETY:

- N/A

FOOTWEAR AND CLOTHING:

- N/A

This toolkit is coproduced by Born in Bradford, Bradford Council, and Faith in Communities. For details, see introduction.

The companions of the Prophet (pbuh) said, "O Messenger of Allah! We do eat but we do not satisfy our apetite." He remarked, "Perhaps you disperse [and eat individually]." They replied, "Yes Indeed!" So he said, "Gather together over your food and mention the name of Allah, you will be granted blessing in it."
[Abu Dawud]

The Prophet (pbuh) said, "Eat, drink and spend in charity without going to excess nor ostentation."
[Al-Mustadrak]

Jabir reported, the Messenger of Allah (pbuh) said, "The food of one person is enough for two people, the food of two people is enough for four, and the food of four is enough for eight."
[Muslim]

*City of Bradford Metropolitan District Council, and Born in Bradford do not own the Islamic Narrative in the toolkit and maintain this to be the independent work of Mufti Mohammed Zubair Butt. All enquiries relating to the Islamic Narrative should be referred to Mufti Mohammed Zubair Butt directly. For detailed disclaimer, please see page 2.

ACTIVITY 1
Discussion

10 mins

Why is portion size important?

Facilitator leads discussion on why portion size is important, sharing the following facts and information on why it is important to have suitable portion size:

- It is important to eat regular meals to help prevent overeating and control the portion size of meals
- Having the right portion size can make it easier for you to digest food
- Our brains and stomachs can get used to eating large portions
- Appropriate portion size is determined by your age, weight, gender and how active you are. Children do not need as much food as adults
- It takes 20 minutes from when you start eating for your stomach to tell your brain you are full, so we should eat slowly and give our bodies time to register fullness
- Salt and sugar are often hidden in our food and drinks, so it is best to avoid adding them to meals. Remember, the portion sizes of salt and sugar. Children should have no more than 3g of salt a day (half a teaspoon) and adults no more than 6g of salt (a teaspoon)
- Sugar allowance for adults is around 6-9 teaspoons. Children should have less than 6 teaspoons per day. Consider that a can of fizzy drink contains around 8 teaspoons! It is recommended that there should be no added sugar in children's diets, with sugar coming from natural sources such as fruit

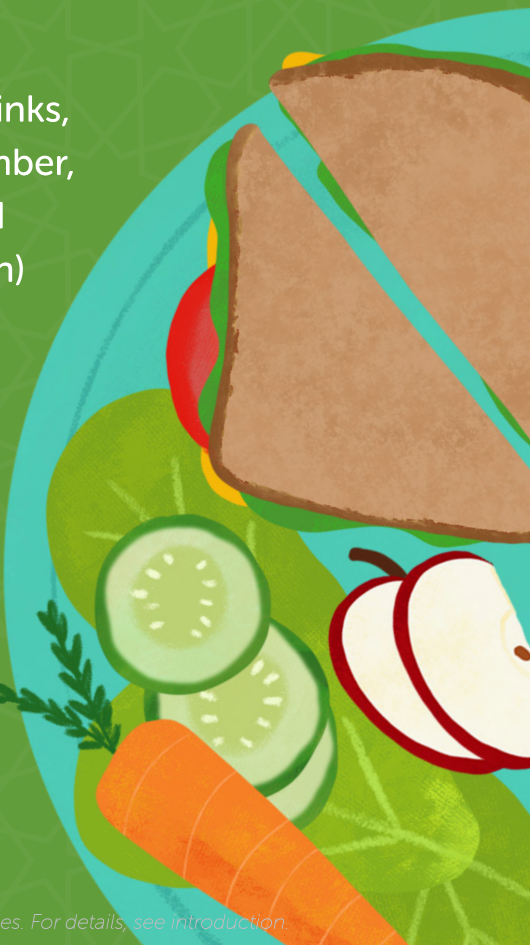

ACTIVITY 2
Personalised Portion Sizes

10 mins

This activity can be done whilst at the faith setting or given as instructions for children to try at home, using the image below for reference.

1. Children draw around their own hands.
2. In different colours, children must draw in the different portion sizes of different foods. For example, draw a line around the thumb indicating the size of a portion of cheese. This will give them their very own portion sizes.

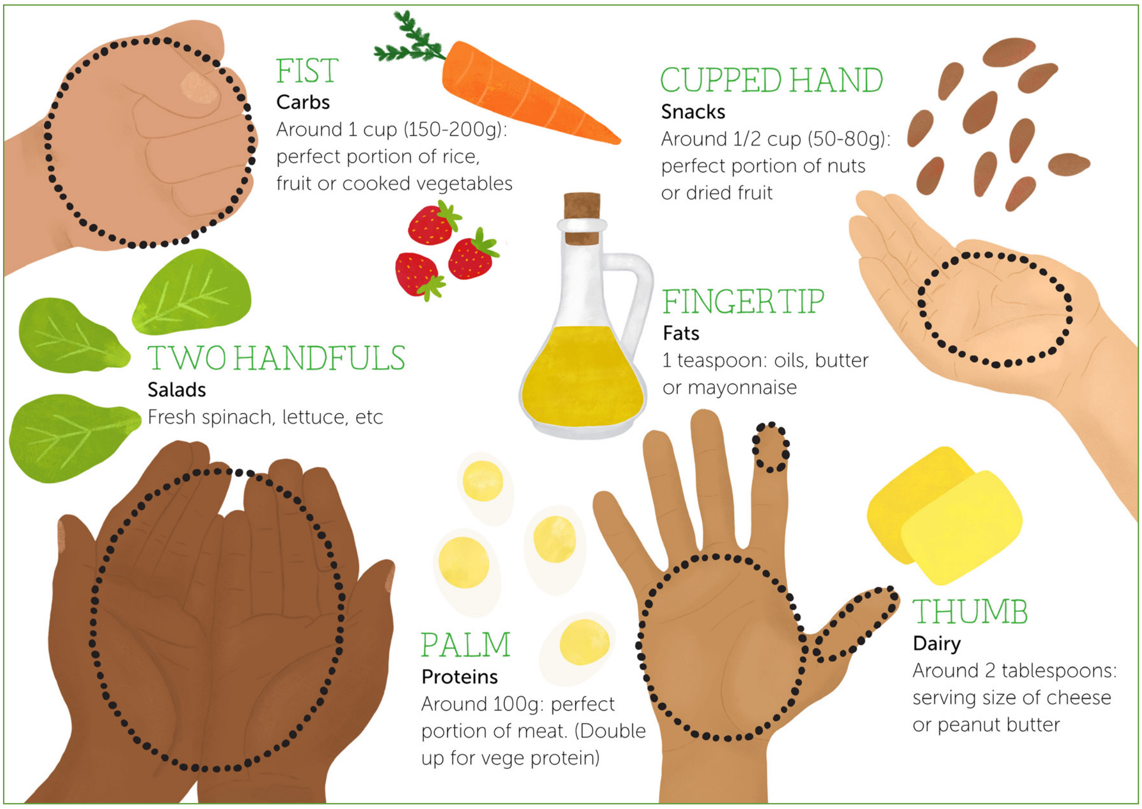

ACTIVITY 3
Guess the Correct Portion

10 mins

Following Activity 2, put children into pairs. One child calls out a food and the other child has to use their hands to demonstrate the right portion size for that food. The first child to get three in a row correct is the winner.

Some examples are below:

Fist: Sweet potato, pineapple chunks, apple
Palm: Chicken breast, beans, unsalted nuts, fish, dahl
Cupped hands: Rice, porridge, bread roll, chapatti
Thumb: Cheese, peanut butter

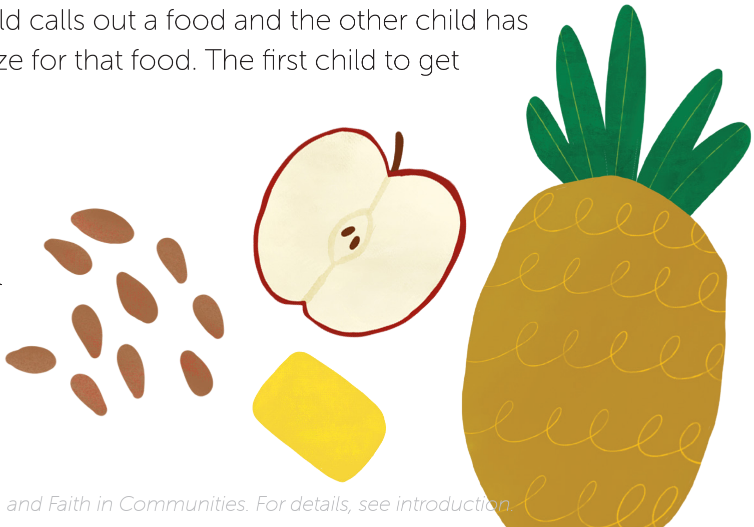

This toolkit is coproduced by Born in Bradford, Bradford Council, and Faith in Communities. For details, see introduction.

PRINT & CUT

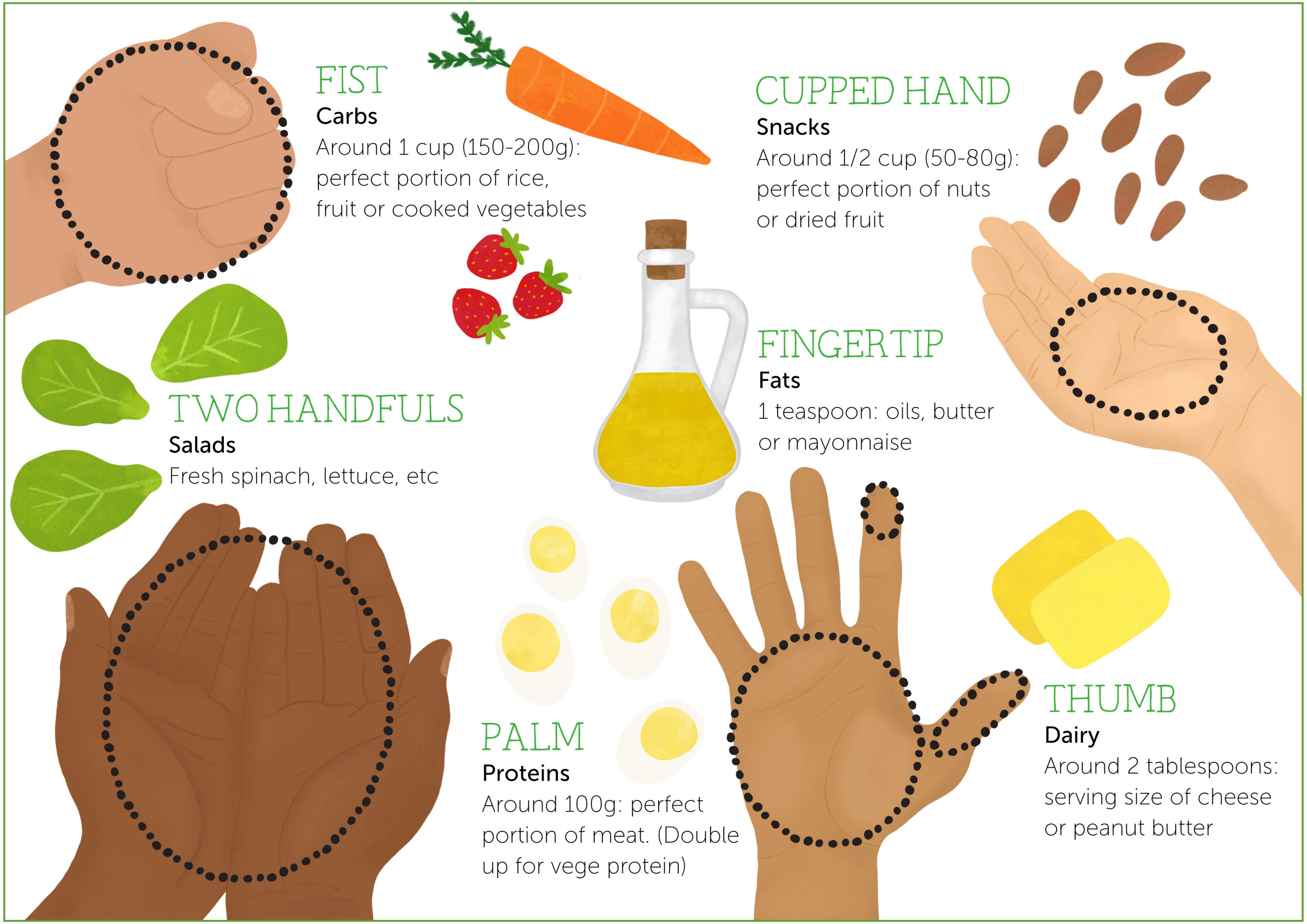

FIST
Carbs
Around 1 cup (150-200g): perfect portion of rice, fruit or cooked vegetables
CUPPED HAND
Snacks
Around 1/2 cup (50-80g): perfect portion of nuts or dried fruit
TWO HANDFULS
Salads
Fresh spinach, lettuce, etc
FINGERTIP
Fats
1 teaspoon: oils, butter or mayonnaise
PALM
Proteins
Around 100g: perfect portion of meat. (Double up for vege protein)
THUMB
Dairy
Around 2 tablespoons: serving size of cheese or peanut butter

HEALTHY DIET WORKSHOP 6

SHOPPING AND FOOD LABELS

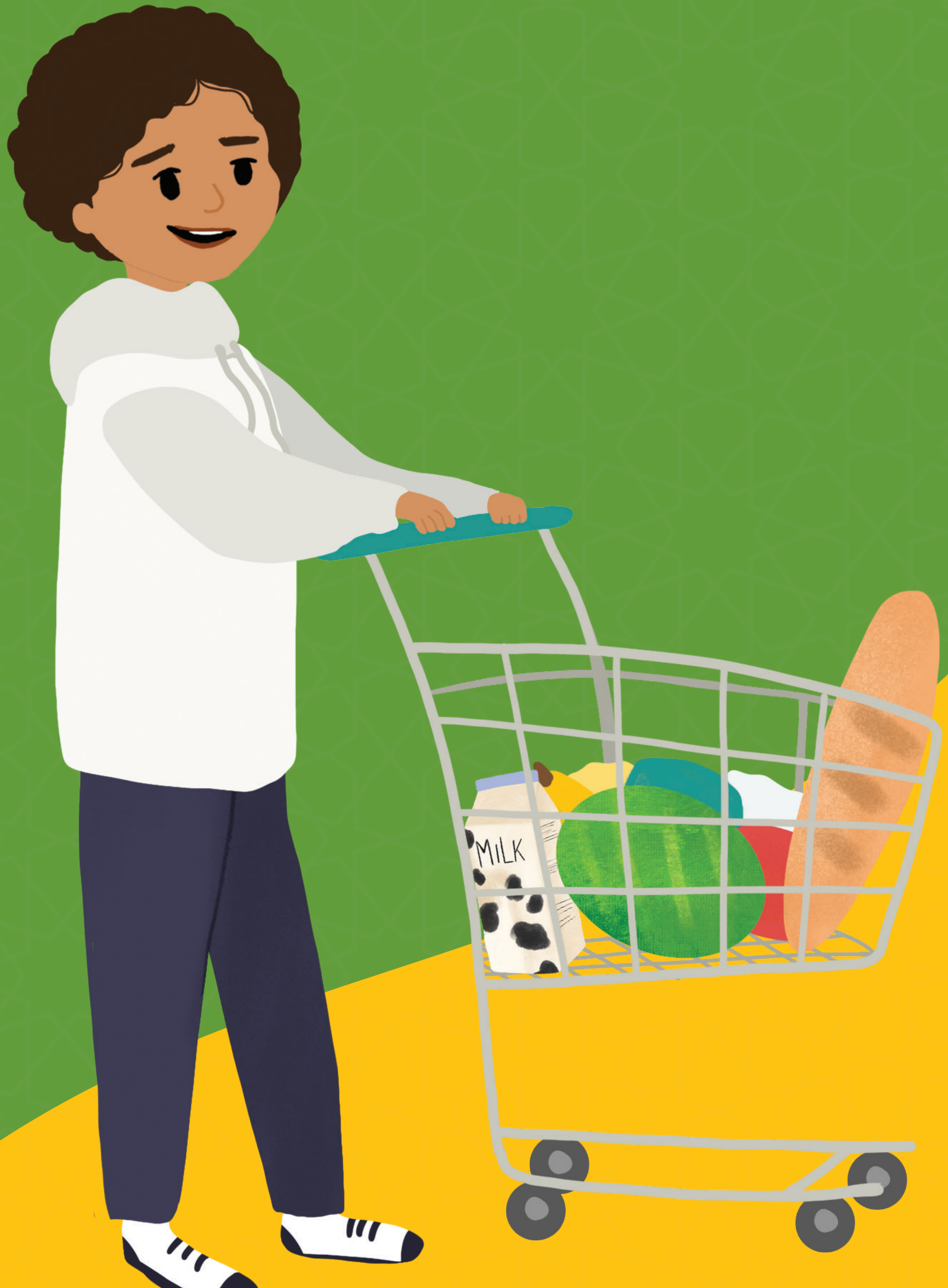

HEALTHY DIET WORKSHOP 6

SHOPPING AND FOOD LABELS

AIMS:

- Children and families gain knowledge about food labels and nutritional information contained on food packaging

OUTCOMES:

- Children and families should be able to identify nutritional information on food labels and determine which is healthier, between two items
- Children gain social and life skills from taking part in family food shopping

TARGET PARTICIPANTS:

- Children and families

LENGTH OF WORKSHOP:

- 1 hour

EQUIPMENT REQUIRED:

Activity 1 - Discussion & Activity 2 - Analysing Food Labels:

- Pens and paper

Activity 3 - Homework:

- Old packaging and labels from items of food from home

LOCATION:

- Classroom and at home (shopping with families)

HEALTH AND SAFETY:

- Parents should be mindful of children going to parts of the supermarket by themselves if taking responsibility for some shopping list items

FOOTWEAR AND CLOTHING:

- N/A

This toolkit is coproduced by Born in Bradford, Bradford Council, and Faith in Communities. For details, see introduction.

Jabir reported,the Messenger of Allah (pbuh) said, “The food of one person is enough for two people, the food of two people is enough for four, and the food of four is enough for eight.”
[Muslim]

Allah mentions in the Holy Qur’an, “And eat and drink but do not be excessive. Indeed, He does not like those who go to excess.
[Qur’an, 7:31]

Miqdam ibn Madikarib reports that he heard the Messenger of Allah (pbuh) saying, “No human ever filled a vessel worse than a stomach. Sufficient for any son of Adam are morsels that keep his back straight. But if it must be, then a third for his food, a third for his drink and a third for his breath.
[Al-Tirmidhi]

*City of Bradford Metropolitan District Council, and Born in Bradford do not own the Islamic Narrative in the toolkit and maintain this to be the independent work of Mufti Mohammed Zubair Butt. All enquiries relating to the Islamic Narrative should be referred to Mufti Mohammed Zubair Butt directly. For detailed disclaimer, please see page 2.

ACTIVITY 1
Discussion

20 mins

To start off the session, the facilitator leads a discussion on the benefits of involving children in family food shopping.

Facilitator can ask children if they currently go food shopping with their parents and families, how they find the experience and what they think they learn by helping with food shopping.

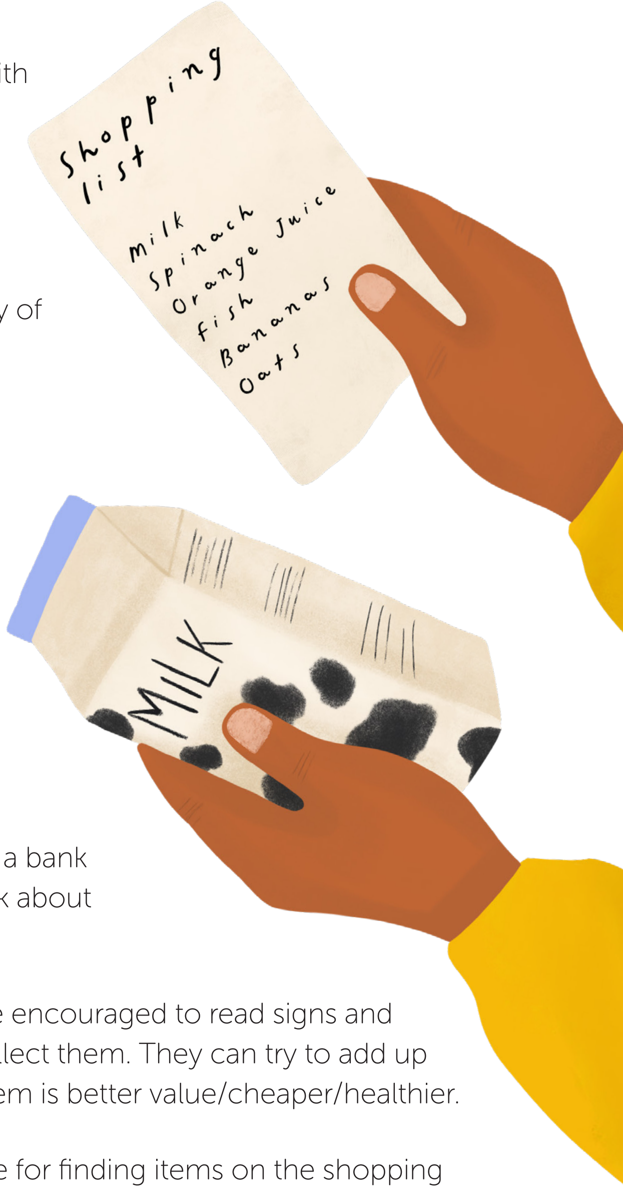

Discussion points should include:

- **Learning about food:** Children can see a large variety of fruit and veg that parents may not buy for home.

- **Reading labels:** Children can learn about labels and advertising, know how to read a label, and compare items.

- **Practical social skills:** Children can acquire practical social skills by seeing the supermarket environment and interacting with a range of different people. They can also practise communication skills and they will learn about waiting their turn, being respectful and asking where an item is.

- **Learning about money:** Children can get hands-on experience of paying at the till and handing over money or a bank card. They can use the self-service scanning till and can talk about how much things cost.

- **Learning letters and numbers:** Children should be encouraged to read signs and symbols and cross off items on the shopping list as you collect them. They can try to add up the prices or work out whether a branded or shop's own item is better value/cheaper/healthier.

- **Can be a fun time out.** If children can be responsible for finding items on the shopping list, it can be a fun searching game. They can swap snacks to healthier alternatives, e.g. fizzy drinks, biscuits, crisps, and chocolate can all be swapped for a healthier item.

At the end of the discussion, the facilitator moves the focus of the discussion to the marketing of food (e.g. awareness of marketing techniques such as 'buy one get one free') and how the nutritional value of food can be seen at a glance.

Key discussion points on the language of food labels are as follows:

- **Ingredients are listed in order of weight:** The main ingredients always come first. So, if the first few ingredients are high-fat ingredients, such as cream, butter, or oil, then that food is a high-fat food. Or, if water is the first ingredient, then you know there is more water than other ingredients.

This toolkit is coproduced by Born in Bradford, Bradford Council, and Faith in Communities. For details, see introduction.

- **Labels:** Can be found on the front and back or sides of packaging.
- **Labels on the front:** Are very useful when you want to compare different food products at a glance. They have squares in 3 colours, RED being high, AMBER being medium and GREEN being low. At a glance, the MORE GREEN on the label means it is healthier, but if there is a lot of RED, then it's high in either fat, saturated fats or sugars. This is a sample label from burger packaging:

Each burger contains:

ENERGY	FAT	SATURATES	SUGARS	SALT
924KJ	13g	5.9g	0.8g	0.7g
220kcal	MED	HIGH	LOW	MED
11%	19%	30%	<1%	12%

% of an adult's reference intake.

Typical values per 100g: Energy 966KJ/230kcal

- **Amounts per 100g:** Labels must include the amount of energy (in KJ and kcal), fat, saturates, carbohydrates, sugars, protein and salt per 100 grams.
- **'Use by':** This is about food safety. There will be a date when you should use the item by. You can freeze foods to extend the 'use by' date.
- **'Best before':** This is about food quality, and for you to know when the item is at its best, before the stated date. Food can still be used after this date as it is just a guide.
- **'No added sugar':** This means sugar has not been added as an ingredient, it does not mean there is no sugar in it. It could have natural sugars like those in fruit or milk.
- **Signs and symbols:** You will also see if the item is suitable for vegetarians or Halal by a sign, or it may be written. It will also state if something is gluten free, sugar free, low fat or low sugar.

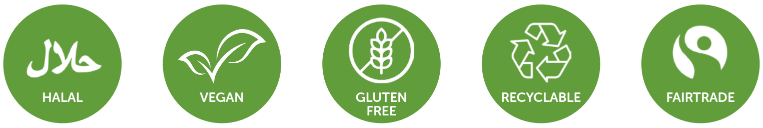

ACTIVITY 2
Analysing Food Labels

20 mins

To consolidate knowledge from the discussion, the facilitator and children bring in labels and packaging from different healthy and unhealthy items, and compare and discuss the amounts of fats, sugars, carbohydrates, etc.

- What other information can the children learn from the packaging?
- Can they identify any of the signs on the packaging? (e.g. vegan, recyclable, Halal produce)
- Would the children buy this in a supermarket?

ACTIVITY 3
Homework

10 mins

This activity can be set as homework for children to complete with their families, and encourages children to pay attention to fruits and vegetables that they do not normally eat.

Action

1. When children go shopping with their families, ask them to find the most unique looking fruit or vegetable. Can the children find a new fruit or vegetable for each colour of the rainbow?
2. Facilitator can encourage the children to buy a new fruit or vegetable, try it and describe the taste.
3. Children discuss their findings with the class the next week.
4. In addition to writing down information, children could draw a giant rainbow and add in all the fruit and vegetables they found.

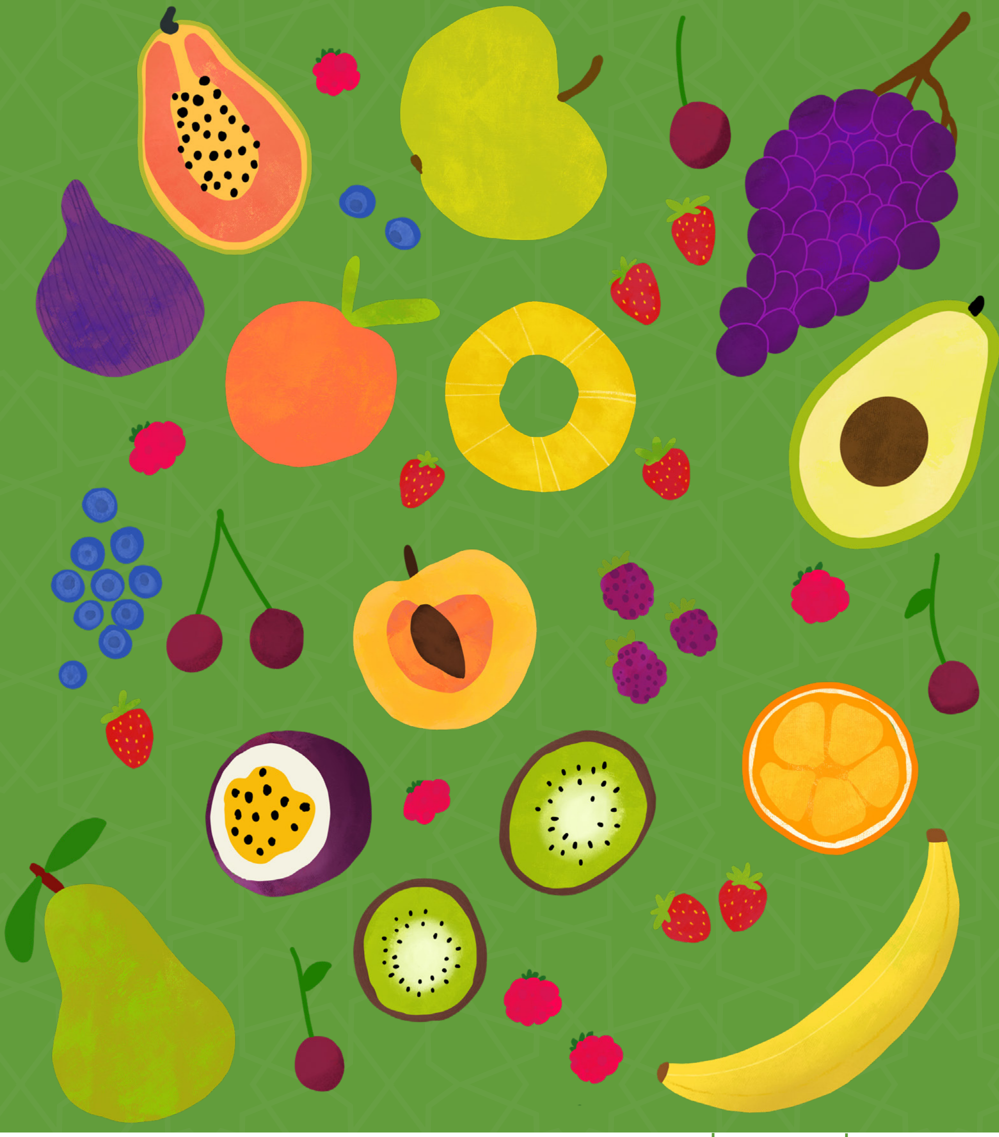

ACTIVITY 4
Homework

10 mins

This homework activity encourages children to discover healthy recipes:

1. Ask the children to pick one healthy recipe with parents, and write a shopping list for it. This will involve the child checking what ingredients they already have at home.
2. They can go with their parents to do the shopping, ensuring that they check labels and buy the correct amount, the healthiest option and at the best price.

? Can they swap some items for a healthier alternative?

We are family BORNINBRADFORD | JU:MP | FAITH IN COMMUNITIES | LIVING WELL | City of BRADFORD METROPOLITAN DISTRICT COUNCIL

Department of Health & Social Care | BRADFORD INSTITUTE FOR HEALTH RESEARCH MAKING RESEARCH REAL | SPORT ENGLAND | Local Government Association

HEALTHY DIET WORKSHOP 7

HEALTHY SNACKING

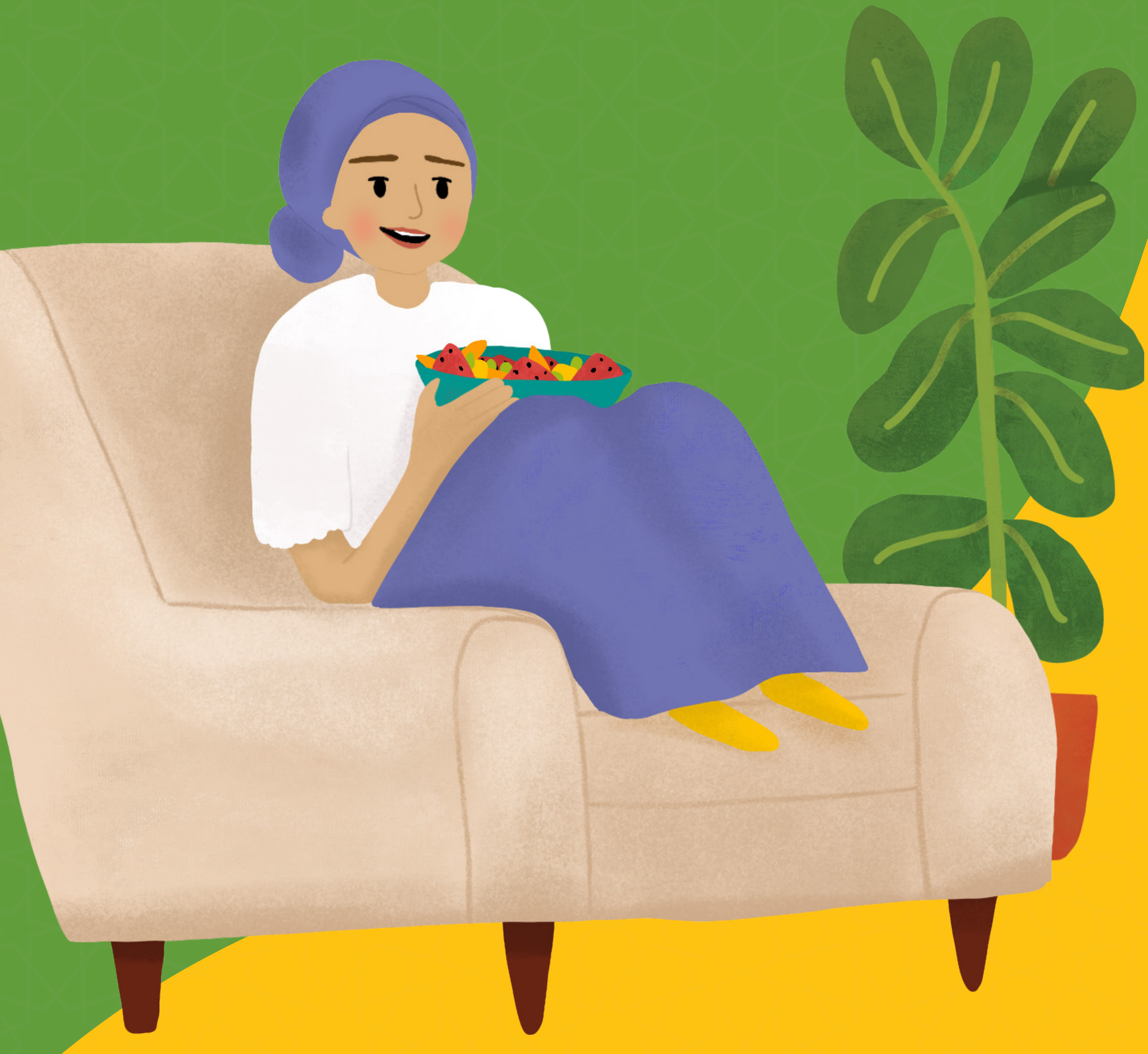

HEALTHY DIET WORKSHOP 7

HEALTHY SNACKING

AIMS:

- Children understand how to substitute unhealthy snacks for healthy ones
- Children, families and faith setting staff and volunteers understand the benefits of healthy snacking

OUTCOMES:

- Children select healthier options when having snacks
- Children use healthy snacking in order to support maintenance of healthy lifestyles and other positive health outcomes (attention levels driven by blood sugar, etc.)
- Children have appropriate number of healthy snacks to maintain a healthy weight

TARGET PARTICIPANTS:

- Children and families, staff and volunteers in faith settings

LENGTH OF WORKSHOP:

- 50 minutes

EQUIPMENT REQUIRED:

Activity 2: Healthy Snacks

- Various fruits and vegetables
- Wooden skewers
- Chopping board and knife

Activity 3: Healthy Swaps

- Whiteboard or blackboard

LOCATION:

- Classroom (and some at home if preferred)

HEALTH AND SAFETY:

- Facilitator must hold Food Hygiene Level 2 award
- Always wash hands, equipment and surfaces before preparing food

FOOTWEAR AND CLOTHING:

- N/A

This toolkit is coproduced by Born in Bradford, Bradford Council, and Faith in Communities. For details, see introduction.

ISLAMIC NARRATIVE

Waqqas reports, “The Messenger of Allah (pbuh) said, ‘Indeed, never do you spend on a living expense seeking with that the pleasure of Allah except that you are rewarded for it, even [for] that [morsel] that you put in your wife’s mouth.”
[Al-Bukhari]

[There are] two blessings in which many people are deceived: good health and free time.
[Al-Bukhari]

And He (Allah) makes lawful for them pure, wholesome things and He makes unlawful for them noxious things.
[Qur’an, 7:157]

Ibn Abbaas reported that the Prophet (pbuh) said, “The blessing descends in the middle of the food, so eat from the two edges of it and do not eat from the middle of it.”
[Al-Tirmidhi]

Jabir reported, The Messenger of Allah (pbuh) said, “The food of one person is enough for two people, the food of two people is enough for four, and the food of four is enough for eight.”
[Muslim]

The Messenger of Allah (pbuh) said,
“I do not eat whilst I am reclining.”
[Al-Bukhari]

*City of Bradford Metropolitan District Council, and Born in Bradford do not own the Islamic Narrative in the toolkit and maintain this to be the independent work of Mufti Mohammed Zubair Butt. All enquiries relating to the Islamic Narrative should be referred to Mufti Mohammed Zubair Butt directly. For detailed disclaimer, please see page 2.

ACTIVITY 1
Discussion

15 mins

Facilitator leads discussion on when snacking can be beneficial, and what kinds of foods make healthy snacks, sharing information and facts with children and families.

Facilitator should ask children what kind of food they eat as snacks currently, what time during the day they usually have a snack, etc.

The discussion should then cover the following information:

- **What is snacking?** Snacking is anything eaten between your main meals. For example, crisps or fruits.
- **What are some common reasons for snacking?** If we feel a little hungry, where we are, social environment, time of day, and just if there is food about, we are tempted but may not be hungry.
- **Is it okay to have a snack?** Although you often hear, 'do not eat in between meals' it is okay to have a snack if we are hungry between meals. 'Snack foods' are usually seen as crisps, biscuits or chocolate, and they are foods which are high in sugar, fat and salt, and low in nutrients. This type of snacking can cause tooth decay and is not good for our health. Fruits, dates, sultanas, nuts or olives are great snack ideas that can be enjoyed if we are hungry between meals.

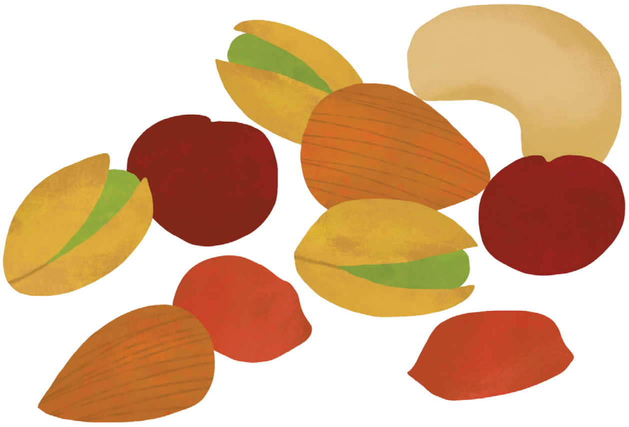

- **What are some good reasons for eating healthier snacks?**
  - Snacking can stop you over eating at mealtimes, which stops you eating too quickly.
  - Snacking can boost your energy levels through the day, between your meals.
  - Snacking helps fuel your brain and body, keeping you alert between meals.
  - Going for long periods of time without food can cause us to eat more food at mealtimes.
  - It is best to plan ahead and eat smaller meals, and have healthy, high fibre, satisfying snacks available throughout the day.

Helpful tips *(The facilitator should reinforce these messages as a summary):*

1. Sometimes when we think we are hungry, we are actually thirsty. Have a drink of water first, then see if you need a snack.
2. Avoid temptation by having fewer unhealthy snacks around at home.
3. Facilitator to discuss with children the effects of advertising on their choices in selecting a snack.

This toolkit is coproduced by Born in Bradford, Bradford Council, and Faith in Communities. For details, see introduction.

4. Facilitator can discuss Sunnah foods with children, and how these could be part of a healthy diet, or eaten as snacks. These can include dates, milk, figs, melon, vinegar, olive oil, mushrooms, grapes, water, pomegranate, barley and honey.

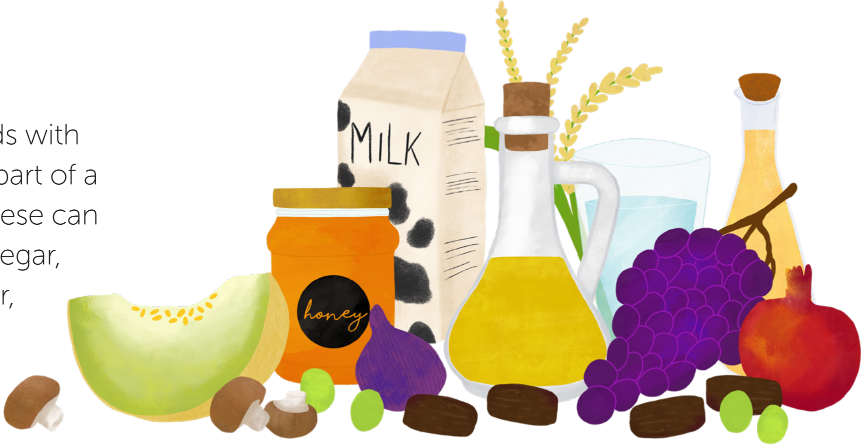

ACTIVITY 2
Healthy Snacks
20 mins

In this activity, children discuss common snack foods and try to think of healthy snack ideas that contain lots of vitamins and minerals. Children can discuss their favourite fruits or vegetables.

Action

1. Facilitator could bring in some varieties that children may not have tasted, e.g. star fruit, guava, papaya, lychee, rambutan, passion fruit, cape gooseberry, coconut in shell, romanesco cauliflower, red and white carrots, parsnips, celeriac, sweet potatoes, sugar snap peas, freeze dried fruit chips, etc.

2. Facilitator to make a fruit kabob with different fruits and vegetables for students to try.

ACTIVITY 3
Healthy Swaps
15 mins

The facilitator encourages children to think about healthy swaps for popular snacks.

The table below includes examples, but children should be encouraged to consider their own, and could use the 'sugar calculator' app created by the NHS *(bit.ly/3CiqmpJ)* to compare popular snack items and find a healthy alternative.

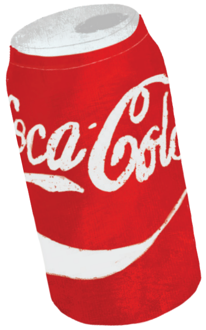

Original Snack	Healthy Swap
Handful of sev mix	Palm-sized portion of dried fruit
A gulab jamun	An apple
1 packet of crisps	2 handfuls of plain popcorn
Full-sized Mars chocolate bar	Bite-sized chocolate bar
Fizzy drink can (330ml)	Fruit-infused water

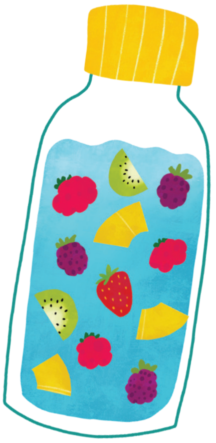

Facilitator can then make a list of some food swaps that the children have found, on a whiteboard or blackboard. Facilitator to discuss how the healthy swaps reduce fat, sugar and salt but also increase the amount of vitamins and minerals children are getting from making smart snack swaps.

This toolkit is coproduced by Born in Bradford, Bradford Council, and Faith in Communities. For details, see introduction.

PHYSICAL ACTIVITY

PHYSICAL ACTIVITY

This section of the toolkit will focus on physical activity. Sessions will be delivered by faith setting staff and/or local champions for physical activity for children and families.

Being more physically active has a range of benefits, including improved physical health, improved mental health, and improved social and emotional wellbeing.

AIMS:

- To embed an ethos of physical activity within the faith setting through a whole setting approach
- To involve and empower staff, leaders, parents and children to be responsible for increasing the physical activity levels of all
- To create sustained changes in behaviour of being physically active within and beyond the faith setting

WORKSHOPS:

1. Reducing Sedentary Time and Increasing Physical Activity
2. Physical Activity in the Curriculum
3. Engaging Parents through Fun Days and Sports
4. Extracurricular and Leisure Time
5. Being Active through Travel
6. Using Islamic Narrative to Support Physical Activity

NOTE:

Please ensure that **Physical Activity Introductory Workshop 1: Reducing Sedentary Time and Increasing Physical Activity** is completed before any of the other physical activity workshops. The remaining physical activity workshops can be completed in any order.

TRAINING:

The JU:MP programme in Bradford offers Children's Physical Activity Training. If you would like to register and get this training, please contact the community engagement manager in your area or email *info@activebradford.co.uk* or *info@faithincommunities.co.uk*

More details are available at *joinusmoveplay.org.* and *faithincommunities.co.uk*

We are family BORNINBRADFORD | JU:MP | FAITH IN COMMUNITIES | LIVING WELL | City of BRADFORD METROPOLITAN DISTRICT COUNCIL

Department of Health & Social Care | BRADFORD INSTITUTE FOR HEALTH RESEARCH MAKING RESEARCH REAL | SPORT ENGLAND | Local Government Association

PHYSICAL ACTIVITY WORKSHOP 1

REDUCING SEDENTARY TIME AND INCREASING PHYSICAL ACTIVITY

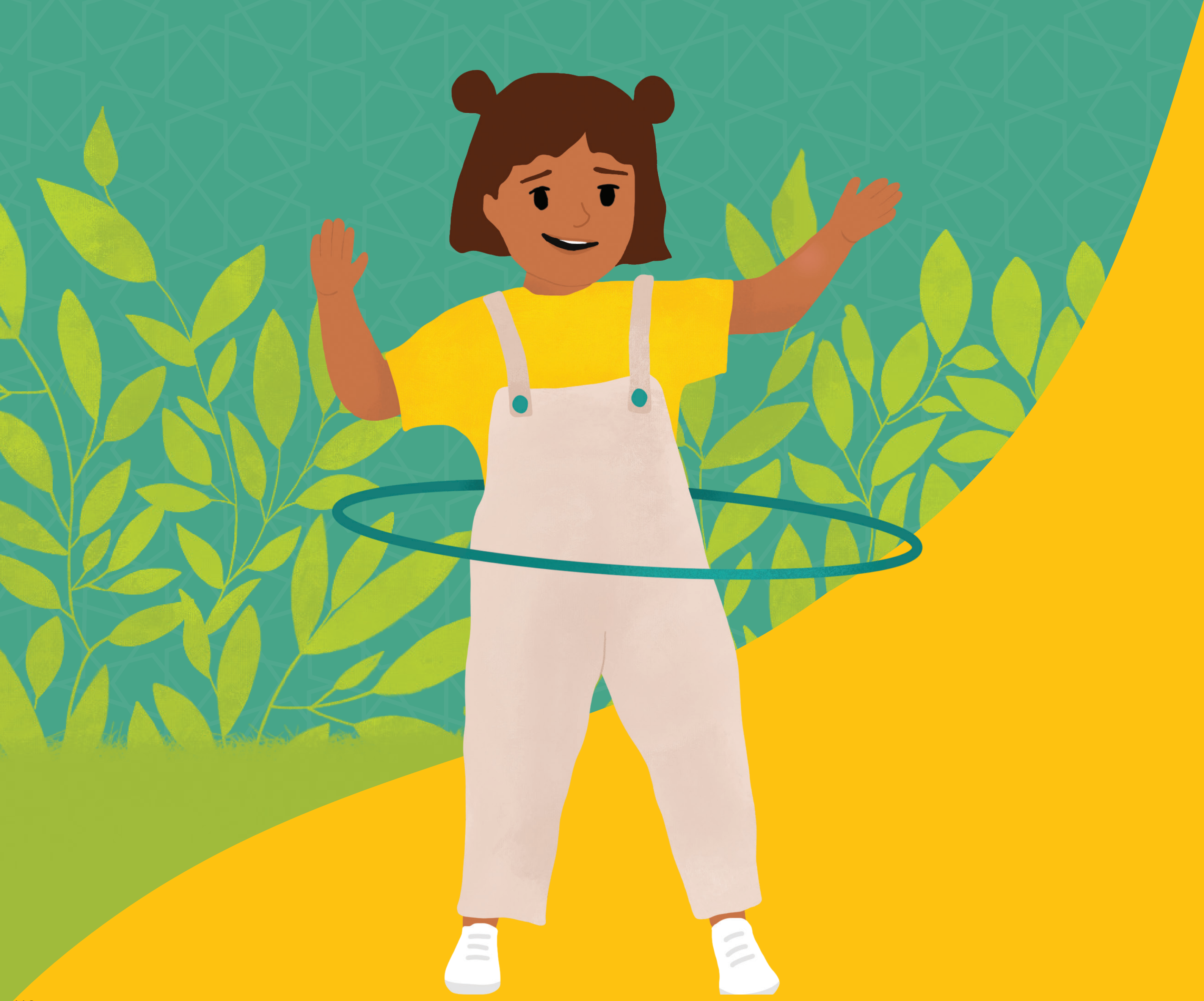

PHYSICAL ACTIVITY WORKSHOP 1

REDUCING SEDENTARY TIME AND INCREASING PHYSICAL ACTIVITY

AIMS:

- Children and families will understand why reducing sedentary time and increasing physical activity is a priority for the faith setting
- Children, families and staff should know the social and health benefits of reducing sedentary time (time spent sitting or not moving) and increasing physical activity
- Priorities for the faith setting are established, with areas to action identified

OUTCOMES:

- Children, families and faith setting staff have a greater understanding of the benefits of reducing sedentary time and increasing physical activity
- Workshop facilitators have an understanding of opportunities to reduce sedentary time and increase physical activity levels
- Priority areas and actions are co-developed to support the motivation of all stakeholders

TARGET PARTICIPANTS:

- Children and parents, staff and volunteers

LENGTH OF WORKSHOP:

- 1 hour

EQUIPMENT REQUIRED:

- Pens and paper

LOCATION:

- Faith setting

HEALTH AND SAFETY:

- Ensure there is adequate space away from walls or furniture when children perform active break activities.

FOOTWEAR AND CLOTHING:

- N/A

And that there is nothing for the human being except what he/she strives [for].
[Qur'an 53:39]

All of you are shepherds [responsible persons], and all of you will be asked about his flock [for whom he is responsible].
[Al-Bukhari]

Abu Huraira reported that the Prophet (pbuh) said, "Whoever goes to the mosque in the morning and evening, Allah will prepare for him a place in Paradise for every morning and evening."
[Al-Bukhari]

O you who believe! When the call is made for prayer on the day of congregation [Friday], hasten to the remembrance of Allah and leave off trade. That is better for you, if you but knew.
[Qur'an, 62:9]

*City of Bradford Metropolitan District Council, and Born in Bradford do not own the Islamic Narrative in the toolkit and maintain this to be the independent work of Mufti Mohammed Zubair Butt. All enquiries relating to the Islamic Narrative should be referred to Mufti Mohammed Zubair Butt directly. For detailed disclaimer, please see page 2.

The Prophet (pbuh) told us that the entire process of attending the mosque, including every step taken to the House of God, is rewarded. “He who purifies himself (performs Wudhu) at his home and then walks to a house from the houses of Allah (mosque) so that he may discharge an obligation from the obligations of Allah (perform an obligatory prayer), one of his two step will wipe out a sin and the other (step) will elevate one rank (in Jannah).”

[Muslim]

In another Hadith, his cousin Ali described his manner of walking as follows: “When he walked, he lifted his leg with vigour. As though he was descending from a high place.”

[Al-Mustadrak]

This toolkit is coproduced by Born in Bradford, Bradford Council, and Faith in Communities. For details, see introduction.

ACTIVITY 1
Discussion

20 mins

The facilitator should introduce what sedentary behaviour is and what physical activity is.

Sedentary time is time spent doing things whilst you are awake and sitting or lying down, that require little energy to do.

Physical activity is any bodily movement produced by skeletal muscles that requires energy expenditure. By doing physical activity that makes your heart beat faster and you breathe harder, you are keeping your body fit and healthy

The facilitator then asks the children what types of activities they might do when they are being sedentary (sitting and lying down).

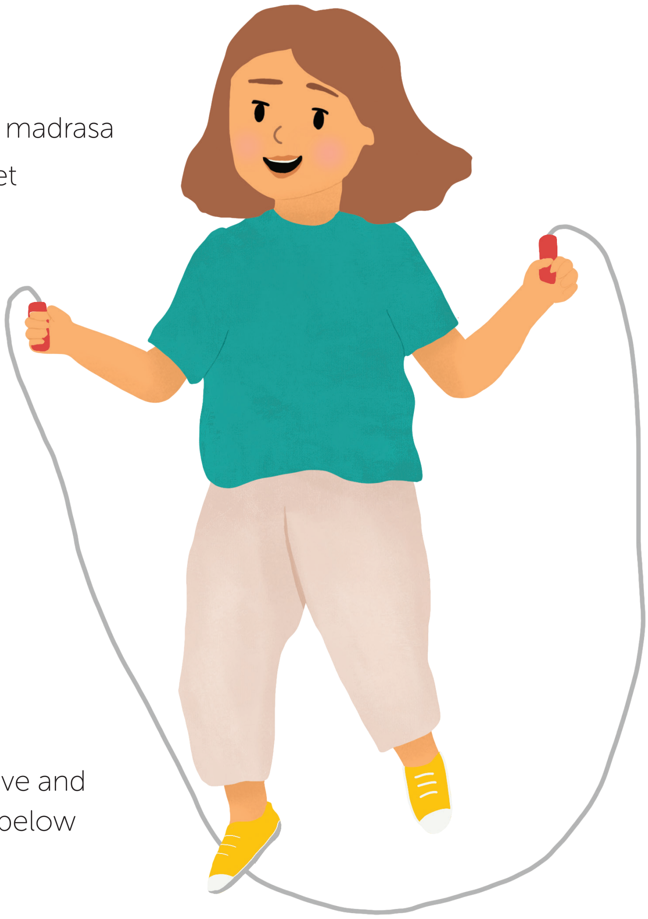

Answers might include:

- Sitting down and learning in a classroom at school or madrasa
- Watching TV, playing computer games, on the internet
- Homework

The facilitator then asks what types of physical activities they might do that would make their hearts beat faster and them breathe harder:

Answers might include:

- Playing in the playground at school
- Playing in the park or garden
- Doing a sport
- Riding a bike
- Fast walking

The facilitator discusses why we need to be physically active and reduce sedentary time. The benefits provided in the table below should be covered in the discussion:

Benefits of Physical Activity for Children: Public Health Review

Physical Health (Physiological)	• Cardio-metabolic health [1]<br>• Muscular strength [1]<br>• Bone health [1]<br>• Cardiorespiratory fitness [1]<br>• Motor skills development [2]<br>• Body composition [2]

1 Consistent evidence
2 Inconsistent evidence or evidence from a small number of studies
3 Insufficient evidence

This toolkit is coproduced by Born in Bradford, Bradford Council, and Faith in Communities. For details, see introduction.

Mental Health (Psychological)	• Self-esteem [1]<br>• Anxiety/stress [1]<br>• Academic achievement [1]<br>• Cognitive function [1]<br>• Attention/concentration [1]<br>• Self-efficacy [2]<br>• Mood [2]<br>• Memory [3]<br>• Body image [3]
Social	• Confidence [1]<br>• Peer acceptance [1]<br>• Positive relationships [2]<br>• Social & communication skills [2]<br>• Self-resilience [2]<br>• School engagement [3]
Behavioural	• Physical activity in adolescence/adulthood [2]<br>• Sleep [2]<br>• Risk-taking behaviour [3]

The facilitator asks children how much physical activity they think they should do every day and then shares the infographic at the end of this workshop, to give them the answer.

ACTIVITY 2
Active Breaks

10 mins

Sedentary time can be reduced through taking active breaks. This activity teaches children four stretches which can, once learned, be used as part of active breaks during teaching sessions at madrasa or at home.

Action

1. Facilitator explains the benefits of stretching to children. Benefits include: improving performance in physical activities, increasing flexibility, improving strength, relaxation and mental wellbeing.

2. If the group is large, the facilitator can split children into smaller groups. Children must be a safe distance away from one another and walls or furniture.

3. Trainer demonstrates each exercise with particular emphasis on maintaining good form (exercises listed below).

1 Consistent evidence
2 Inconsistent evidence or evidence from a small number of studies
3 Insufficient evidence

This toolkit is coproduced by Born in Bradford, Bradford Council, and Faith in Communities. For details, see introduction.

4. After each demonstration, children perform the stretch for 30 seconds. Facilitator to check for correct posture, and offer encouragement and praise.

5. Repeat for all exercises.

Optional: facilitator can create a points system and allocate points based on good stretching posture, good level of effort in the activity, etc. This could be a competition in class.

Exercise 1: Twist and Touch

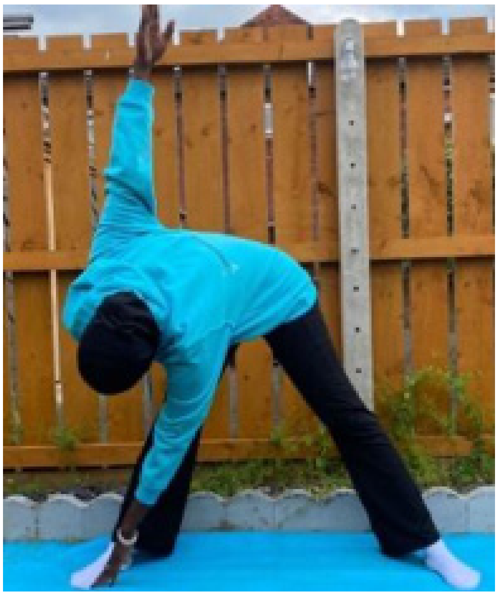

1. Stand with arms outstretched in line with the shoulders.

2. Maintaining arms in this position, bend and twist body down so that right hand touches (or gets as close to touching) left foot, while ensuring arms are maintained in the outstretched position throughout.

3. Return to start position and repeat with opposite side.

Emphasis is on form and not the amount completed – do not rush.

Exercise 2: Arm Swing

1. Starting with hands by your sides, swing your arms in a controlled fashion in a circle up past your ears and down again.

Adaption to this exercise can be for children to swing each arm in the opposite direction e.g. left arm to swing in a clockwise motion while right arm moves anti-clockwise.

Exercise 3: Slide Stretch

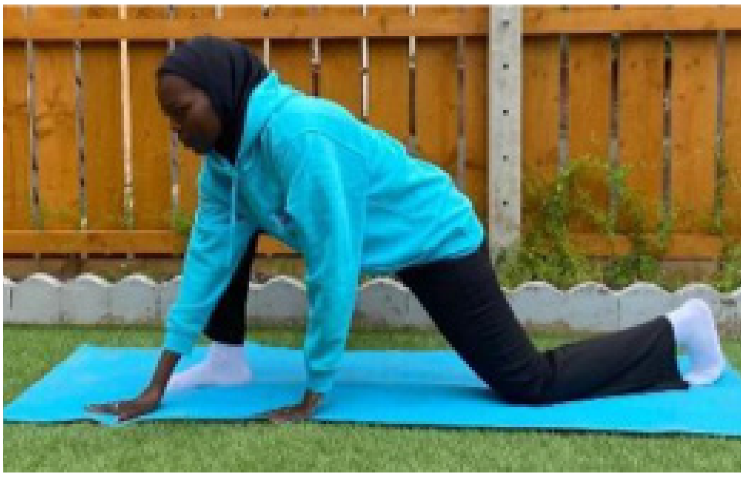

1. Kneel down with left leg outstretched behind, and the right leg bent at 90 degrees in front.

2. Slowly straighten the right leg and move it forwards as much as comfortable (into splits position) and hold, leaning upper body forwards.

3. Repeat with the opposite leg.

Exercise 4: Bent-over Arm and Shoulder Stretch

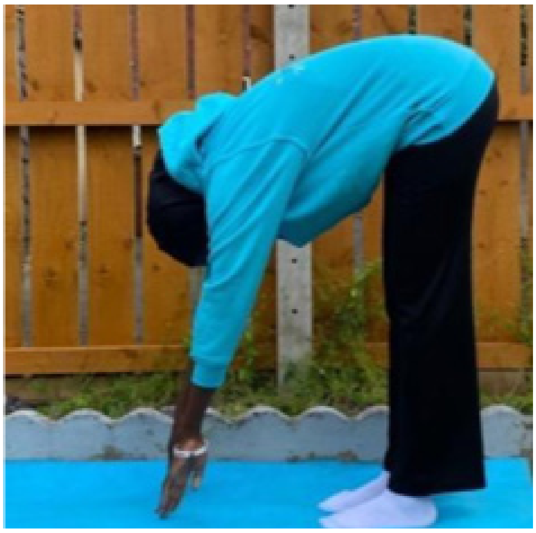

1. Raise both arms until they are in line with the shoulders.

2. Maintain a relaxed posture and bend upper body down to 90 degrees, ensuring head is facing towards the ground and chin is not tucked in towards the neck.

This can be likened to Ruku position during prayers.

ACTIVITY 3
Mind Map
30 mins

In this activity, the workshop facilitator will split participants into groups.

For the first 20 minutes, groups will create a mind map of ideas (a diagram with the central idea written in the middle of the page, and associated ideas arranged around it) of how to reduce sedentary time (time spent sitting or not moving) in the following four scenarios:

1. Faith setting (during curriculum time)
2. Home (with families and independently)
3. Extracurricular and leisure time
4. Travel

For the last 10 minutes, the mind mapping ideas should be shared with the whole group.

Once all groups have shared their ideas, all participants will vote on which of the four settings (faith setting, home, extracurricular, or travel) for increasing physical activity is most important for the community.

The results of this vote need to be fed back to the health group to help decide the order in which the rest of the physical activity workshops are delivered (i.e. the setting with the most votes could be the next workshop to be delivered).

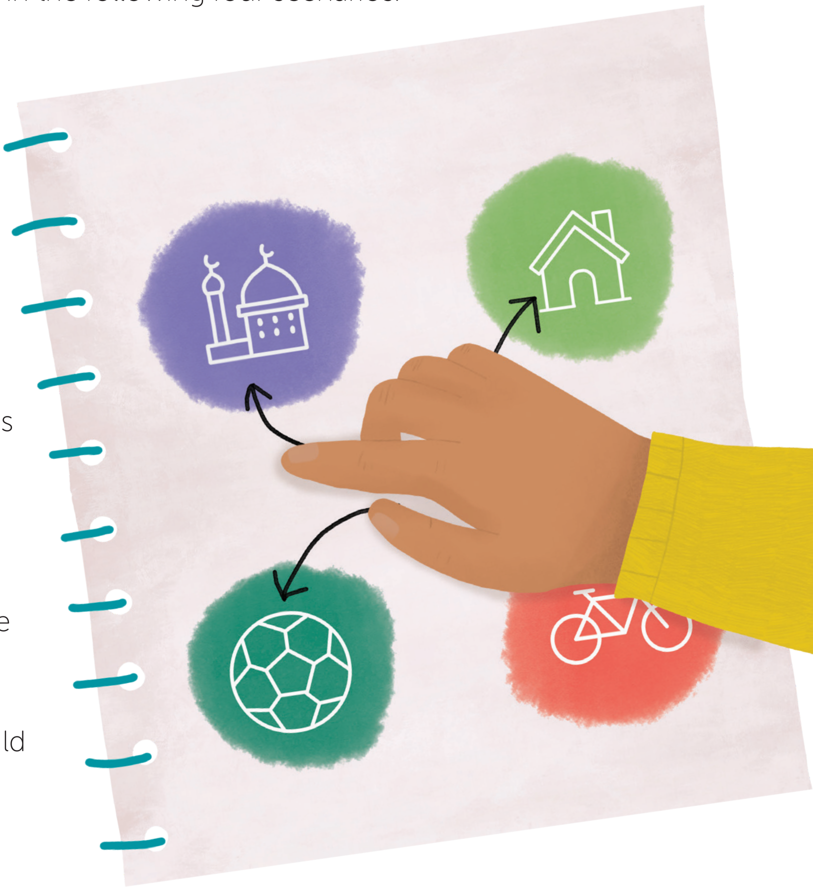

This toolkit is coproduced by Born in Bradford, Bradford Council, and Faith in Communities. For details, see introduction.

PRINT
&
CUT

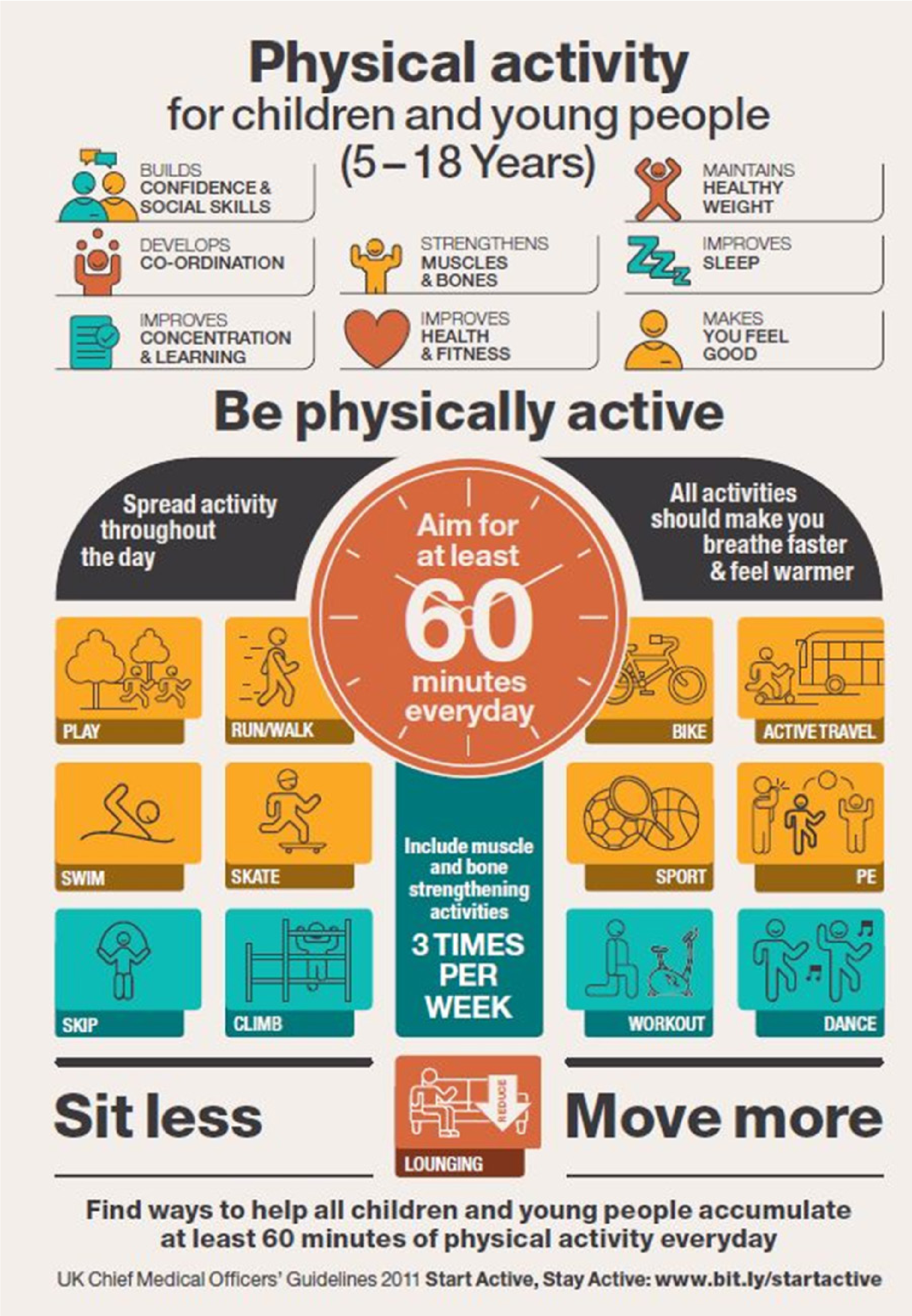
Physical activity
for children and young people
(5–18 Years)
BUILDS CONFIDENCE & SOCIAL SKILLS
MAINTAINS HEALTHY WEIGHT
DEVELOPS CO-ORDINATION
STRENGTHENS MUSCLES & BONES
IMPROVES SLEEP
IMPROVES CONCENTRATION & LEARNING
IMPROVES HEALTH & FITNESS
MAKES YOU FEEL GOOD
Be physically active
Spread activity throughout the day
Aim for at least 60 minutes everyday
All activities should make you breathe faster & feel warmer
PLAY
RUN/WALK
BIKE
ACTIVE TRAVEL
SWIM
SKATE
Include muscle and bone strengthening activities 3 TIMES PER WEEK
SPORT
PE
SKIP
CLIMB
WORKOUT
DANCE
Sit less
REDUCE
LOUNGING
Move more
Find ways to help all children and young people accumulate at least 60 minutes of physical activity everyday
UK Chief Medical Officers' Guidelines 2011 Start Active, Stay Active: www.bit.ly/startactive

PHYSICAL ACTIVITY WORKSHOP 2

PHYSICAL ACTIVITY IN THE CURRICULUM

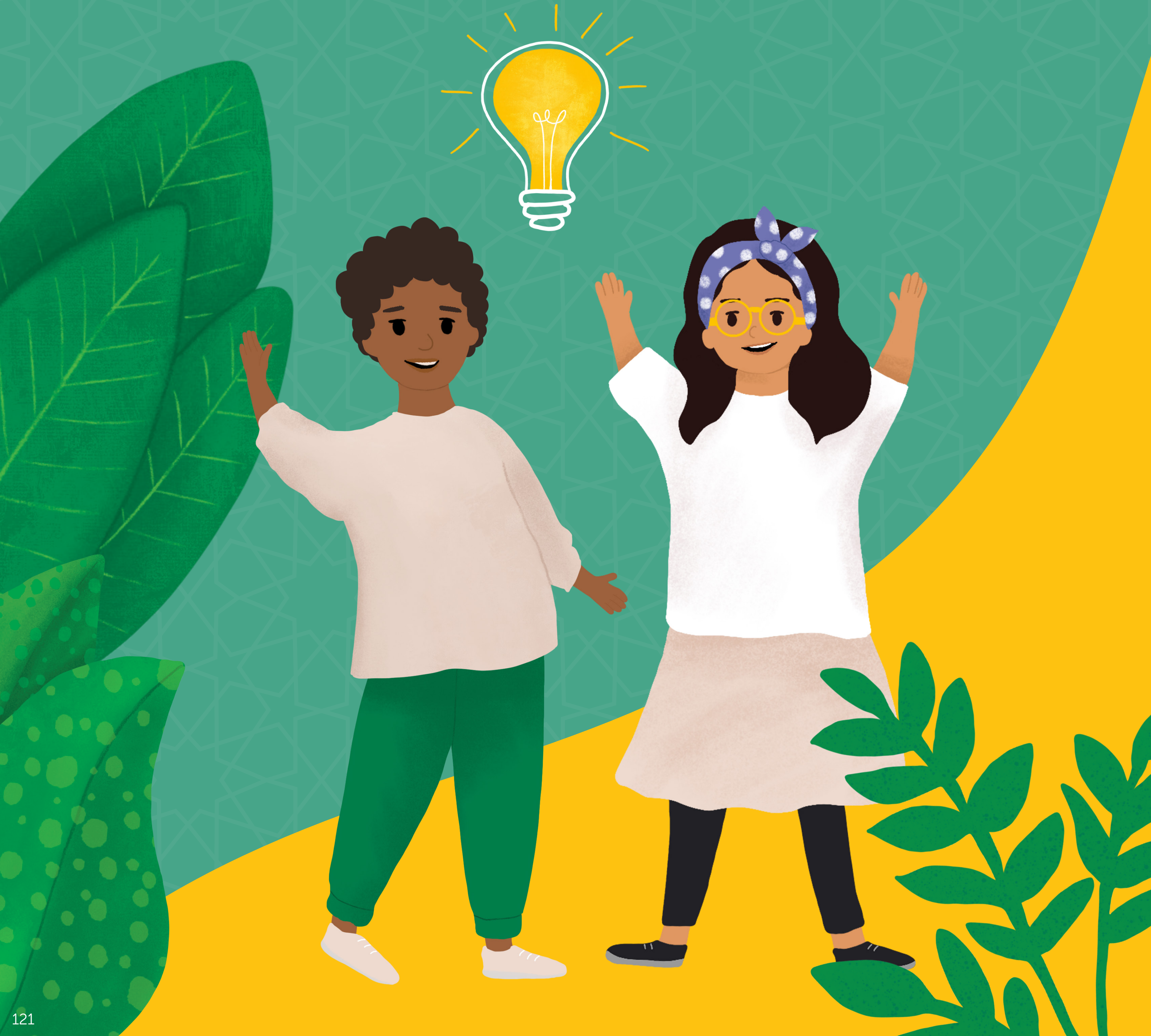

PHYSICAL ACTIVITY WORKSHOP 2

PHYSICAL ACTIVITY IN THE CURRICULUM

AIMS:

- Faith setting teachers learn about the importance of active learning, what active learning looks like in practice, and develop a plan for implementing this in the faith setting

OUTCOMES:

- Faith setting teachers and leaders will have increased confidence in delivering active learning inside the faith setting
- Teachers will develop their understanding of the benefits of active learning in the curriculum and how it looks in practice
- Teachers will be provided with the opportunity to bring active learning into the curriculum

TARGET PARTICIPANTS:

- Faith setting staff and volunteers

LENGTH OF WORKSHOP:

- 45 minutes

EQUIPMENT REQUIRED:

- N/A

LOCATION:

- Faith setting

HEALTH AND SAFETY:

- N/A

FOOTWEAR AND CLOTHING:

- N/A

This toolkit is coproduced by Born in Bradford, Bradford Council, and Faith in Communities. For details, see introduction.

And that there is nothing for the human being except what he/she strives [for].

[Qur'an 53:39]

All of you are shepherds [responsible persons], and all of you will be asked about his flock [for whom he is responsible].

[Al-Bukhari]

Aaisha, the wife of the Prophet (pbuh) reports, "I was with the Prophet (pbuh) on a journey whilst I was [still] young. The Prophet (pbuh) said to his companions, 'Come forward!' So they came forward. Then he said [to me], 'Come, let me race with you.' So I raced with him and raced ahead of him on foot. After some time, I went again on a journey with him and he said to his companions, 'Come forward!' Then he said, 'Come, let me race with you.' I had forgotten what had happened [before] and had gained weight and so I said, How can I race with you, O Messenger of Allah! Whilst I am in this state?' He replied, 'You must do so.' So I raced him and he beat me in the race. Then he said,' This is for that win.'"

[Sunan al-Baihaqi]

*City of Bradford Metropolitan District Council, and Born in Bradford do not own the Islamic Narrative in the toolkit and maintain this to be the independent work of Mufti Mohammed Zubair Butt. All enquiries relating to the Islamic Narrative should be referred to Mufti Mohammed Zubair Butt directly. For detailed disclaimer, please see page 2.

The Prophet (pbuh) used to pray whilst carrying his granddaughter, Umamah, daughter of Zainab and Abu al-Aas on his shoulders. When he prostrated he put her down and when he stood up he picked her up.
[Al-Bukhari, Muslim]

Abdullah ibn Shaddaad reports from his father, "The Messenger of Allah (pbuh) came out to us in one of the evening prayers (Maghrib or Isha) whilst he was carrying Hasan or Husain. The Messenger of Allah (pbuh) came forward, put him down and said Takbir (Allaahu akbar) for the prayer. He then prayed and prostrated during his prayer a prostration that he lengthened. My father said, 'I raised my head and saw the child on the back of the Messenger of Allah (pbuh) whilst he was prostrating so I went back to my prostration.' When the Messenger of Allah completed the prayer, the people said to him, 'O Messenger of Allah! Indeed you prostrated during your prayer a prostration that you lengthened until we thought that something had happened, or that you were receiving Revelation.' He said, 'None of that happened, but my son was riding on me and so I did not want to hurry him up until he had had enough.'"
[Sunan al-Nasa'i]

This toolkit is coproduced by Born in Bradford, Bradford Council, and Faith in Communities. For details, see introduction.

ACTIVITY 1
Discussion

25 mins

The facilitator tells the group what physically active learning is.

Physically active learning is the integration of movement within delivery of academic content.

The facilitator leads a discussion and encourages participants to reflect on the following questions:

- **How can learning be physically active whilst maintaining purpose?**
- **Why is it important to have physically active learning?**
- **What are the benefits of physically active learning?**

In order to do this:

1. The facilitator will create small groups of attendees.
2. Facilitator explains benefits of physical activity during curriculum time (see Figure 1).
3. Facilitator will then explain the methods that can be used to incorporate physically active learning within the faith setting (see Figure 2).
4. All groups will be asked to produce a mind map of ideas of how they can incorporate active learning strategies into current practice within a faith setting classroom.
5. After 15 minutes, groups will feedback and discuss potential changes to their teaching of the curriculum in the madrasa.

Figure 1: Benefits of Physical Activity for Children (Public Health Review)

Physical Health (Physiological)	• Cardio-metabolic health [1]<br>• Muscular strength [1]<br>• Bone health [1]<br>• Cardiorespiratory fitness [1]<br>• Motor skills development [2]<br>• Body composition [2]
Mental Health (Psychological)	• Self-esteem [1]<br>• Anxiety/stress [1]<br>• Academic achievement [1]<br>• Cognitive function [1]<br>• Attention/concentration [1]<br>• Self-efficacy [2]<br>• Mood [2]<br>• Memory [3]<br>• Body image [3]

1 Consistent evidence
2 Inconsistent evidence or evidence from a small number of studies
3 Insufficient evidence

This toolkit is coproduced by Born in Bradford, Bradford Council, and Faith in Communities. For details, see introduction.

Social	• Confidence [1]<br>• Peer acceptance [1]<br>• Positive relationships [2]<br>• Social & communication skills [2]<br>• Self-resilience [2]<br>• School engagement [3]
Behavioural	• Physical activity in adolescence/adulthood [2]<br>• Sleep [2]<br>• Risk-taking behaviour [3]

Figure 2: Move & Learn Approaches

Retrieval (prior knowledge)	• Physically retrieving information<br>• Recalling facts through movement<br>• Move to respond to multiple choice questions
Collection (new knowledge)	• Move to collect new information and strengthen knowledge<br>• Collect information from a range of environments
Modelling	• Children model new knowledge & skills in active way<br>- Modelling mathematical concepts<br>- Role play
Connection	• Group tasks to share learning & resolve misconceptions<br>- Conversation stations<br>- Move, pair & share<br>- Move, view & review
Creation	• Use connections to transfer learning into new contexts<br>- Using movement to tell a story<br>- Constructing a pictogram and models using natural objects<br>- Making a bug hotel based on their knowledge of habitats<br>- Compose and perform music using body percussion<br>- Create large scale maps using chalks / natural items / PE equipment

1 Consistent evidence
2 Inconsistent evidence or evidence from a small number of studies
3 Insufficient evidence

This toolkit is coproduced by Born in Bradford, Bradford Council, and Faith in Communities. For details, see introduction.

ACTIVITY 2
Suggestions for Promoting Active Learning

20 mins

After discussing feedback from the previous discussion activity, the facilitator will highlight the following suggestions as additional techniques for promoting active learning in the faith setting. *Please note that the activities below are suggestions, and can be adapted to best suit the workshop attendees.*

- **Pairs Share:** Children are split into pairs when undertaking a task, such as learning and sharing their lesson with their partner while walking, or one child (while sitting in a circle) can go to another child in the opposite direction to share what they are reading. They can then note these ideas down on a clipboard and share their ideas with the other groups.

- **Summary on Board:** Children will be given a post-it note and, throughout the lesson (possibly every 20 minutes), be encouraged to write down what they have learnt so far and move to stick it to a designated area of the classroom.

- **Prayer Postures:** The teacher will describe and demonstrate prayer postures to the students. Each child will form a pair and they will then practise the prayer postures. The teacher will facilitate by walking around the room and prompting or helping the children as a form of physical activity. The children will then each be asked to perform all the positions of prayer in one go. They will be awarded a point for every correct position.

- **Move, Pair & Share:** Children will all be asked to come to a table. The teacher will have written the names of different prophets on separate pieces of paper, which are folded so that the name is then hidden. The children will then pick one name and be asked to think carefully about that prophet. They will then move around the room until the teacher tells them to stop, when they will then describe the prophet to the person closest to them, without ever mentioning their name. It would be up to the other children to guess. The child that guesses correctly will then be asked to describe the prophet that they picked. This will continue until all the children have described a specific prophet.

- **Move, View & Review:** The students are given paper and asked to draw anything - suggestions could be the Ka'bah, the Dome of the Rock, or their own design of a mosque. Alternatively, the children could write a short story or a piece of text. They will then be asked to put this up on the wall, and all students will then be asked to go on a gallery walk, reviewing and providing kind and productive feedback on their peers' work.

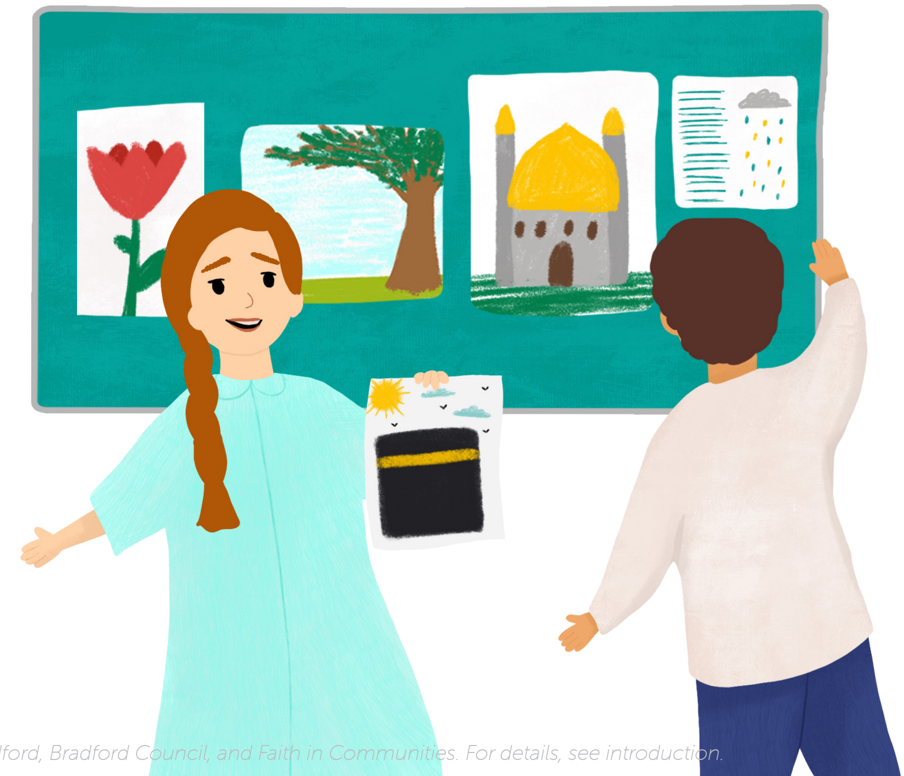

This toolkit is coproduced by Born in Bradford, Bradford Council, and Faith in Communities. For details, see introduction.

PHYSICAL ACTIVITY WORKSHOP 3

ENGAGING PARENTS THROUGH FUN DAYS AND SPORTS

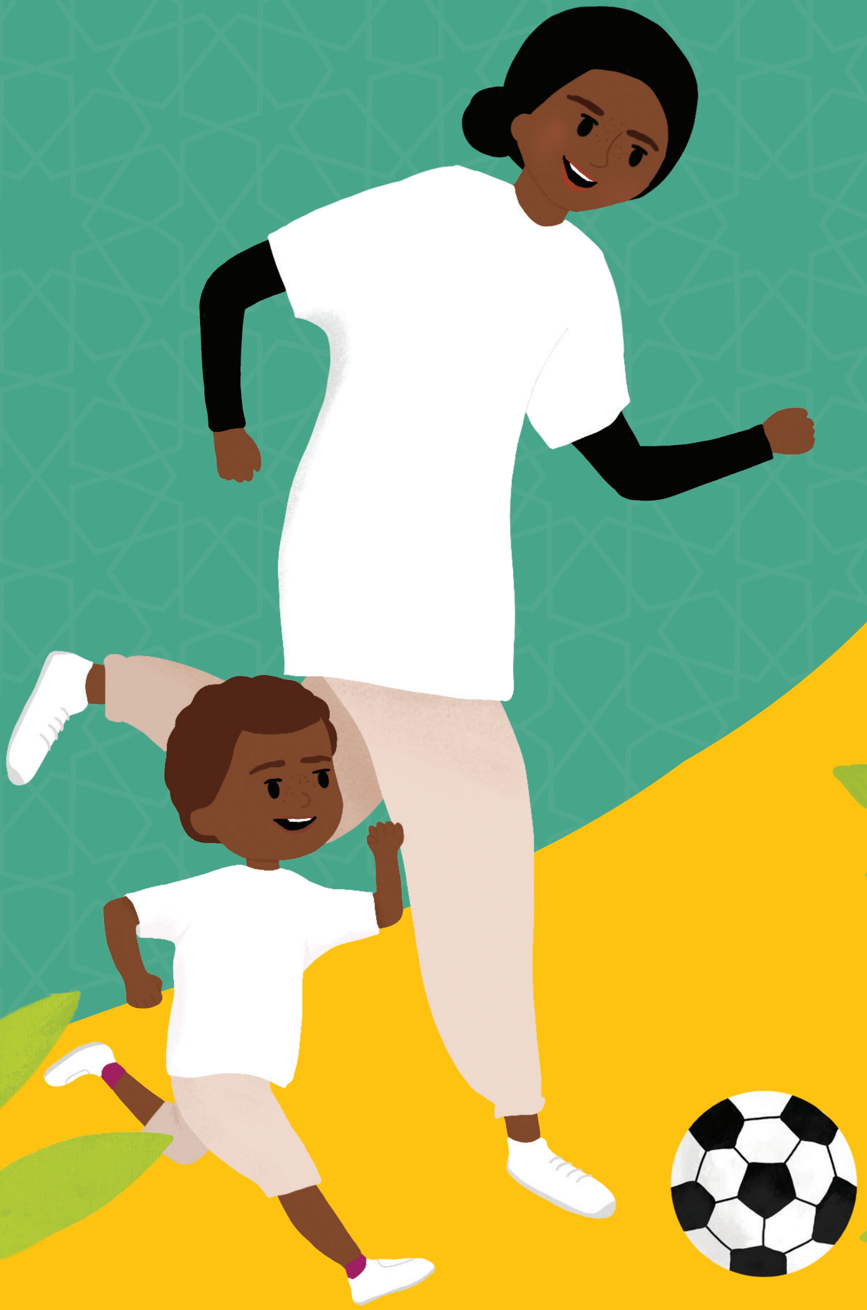

PHYSICAL ACTIVITY WORKSHOP 3

ENGAGING PARENTS THROUGH FUN DAYS AND SPORTS

AIMS:

- To engage parents in physical activities run by or associated with the faith setting
- To increase the physical activity levels of children and families within and beyond the faith setting

OUTCOMES:

- New opportunities are created for children and families to participate in physical activity
- Children are supported to take part in new activities
- Families are motivated to take part in activities outside of the faith setting

TARGET PARTICIPANTS:

- Parents, staff and volunteers

LENGTH OF WORKSHOP:

- 40 minutes

EQUIPMENT REQUIRED:

- Pens and paper

LOCATION:

- Faith setting

HEALTH AND SAFETY:

- N/A

FOOTWEAR AND CLOTHING:

- N/A

This toolkit is coproduced by Born in Bradford, Bradford Council, and Faith in Communities. For details, see introduction.

ISLAMIC NARRATIVE

One of the two women said, "O my father, hire him. Indeed, the best whom you might hire is the strong and the trustworthy."
[Qur'an, 28:26]

"And do not throw [yourselves] with your [own] hands in to destruction."
[Qur'an, 2:195]

Usman Ibn Maz'un was a companion of the Prophet (pbuh) who had decided to put himself through some hardship. The Prophet (pbuh) came to learn of this and advised him against this saying, "Indeed, your body has a right over you."
[Abu Dawud]

Whilst offering advice to a man, the Prophet Mohammed (pbuh) said, "Value five [things] before five [other things]: Your youth before your old age, your health before your illness, your affluence before your poverty, your availability before your occupation, and your life before your death."
[Al-Mustadrak]

*City of Bradford Metropolitan District Council, and Born in Bradford do not own the Islamic Narrative in the toolkit and maintain this to be the independent work of Mufti Mohammed Zubair Butt. All enquiries relating to the Islamic Narrative should be referred to Mufti Mohammed Zubair Butt directly. For detailed disclaimer, please see page 2.

Prophet Moses was lauded for his bodily strength. When Prophet Moses fled Egypt out of fear of being killed by Pharaoh and his men, he travelled during the nights towards Midian whilst hiding during the day.

After eight nights he arrived at the watering hole of Midian where he found a band of shepherds watering their sheep, whilst two young women were holding their sheep back and preventing them from mixing with the others. Prophet Moses asked them why they did not join with the rest of the shepherds and they explained that they were waiting for them to finish first and, as their father was very old, they had to do the work normally done by a man. Prophet Moses offered to water their sheep for them but when he approached the watering hole, he saw that the shepherds had put a large rock over the mouth of the hole, that could only be moved by ten men. Prophet Moses embraced the rock and lifted it away single handed from the hole and then returned it after he had watered their sheep.

The two young women returned home earlier than usual that day, which was a surprise for their father. They relayed to him what had transpired and one of them suggested that he should hire Prophet Moses to do the manual work around the house as Prophet Moses was both trustworthy and physically strong.
[Ibn Kathir, Stories of the Prophets]

ACTIVITY 1
Discussion

20 mins

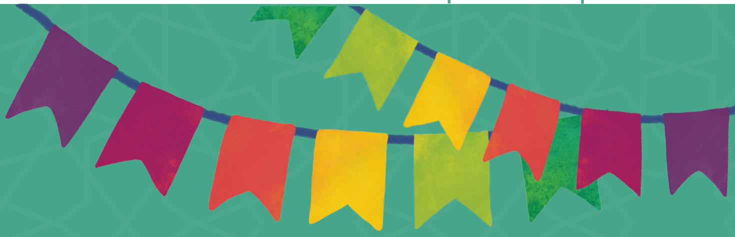

Facilitators lead a discussion and planning for an activity on how parents can be mobilised and motivated to be part of the active faith setting.

How to encourage parental participation:

1. Participants share ideas on how parents can be encouraged to participate in faith setting organised sports and fun days.

2. Facilitator will outline the importance of parental involvement in the education of children and how physical activity is a great way to bond with their child, and contribute to a wide range of positive physical and mental health outcomes.

3. Groups will be asked to produce a mind map of ideas (a diagram with the central idea written in the middle of the page, and associated ideas arranged around it) of the physical activities that can be carried out during a fun day in line with parents' interests, skillsets and capabilities.

4. After creating the mind map, participants are asked to highlight barriers and enablers for engagement.

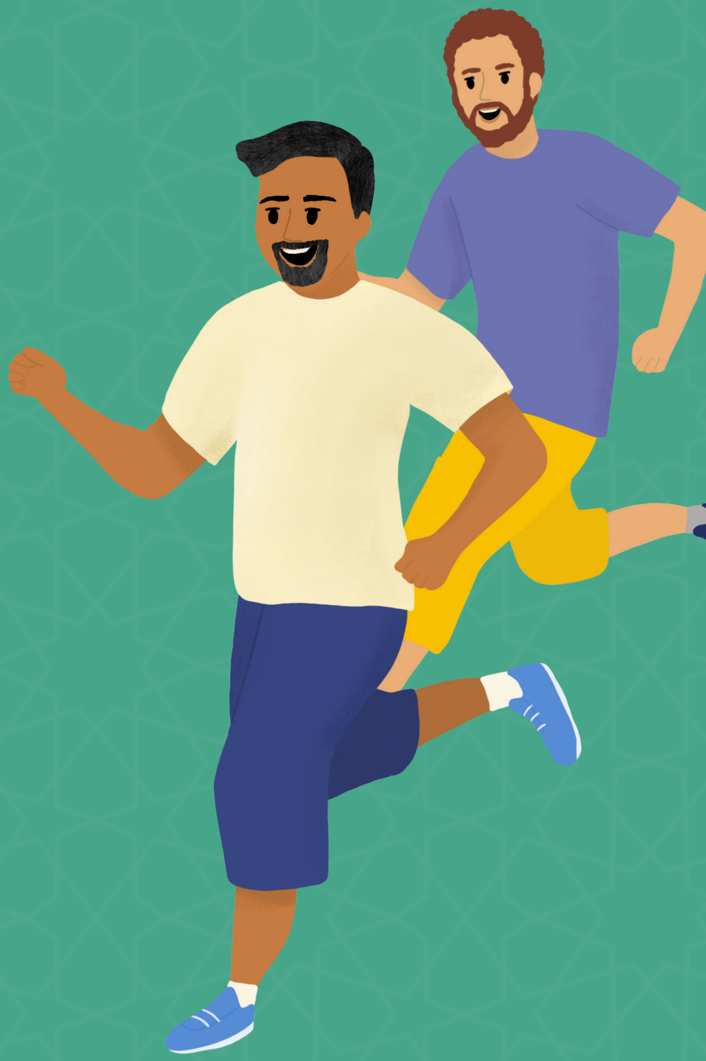

Please note that it is essential to ensure that any parental involvement in religious setting activities are in line with policies and regulations including, but not limited to, insurance, DBS checks, risk assessments, and relevant qualifications.

The following ideas are ways in which parents can get involved in organised fun days within the religious setting:

- **Faith setting delivers a fun/activity day:** The faith setting could create a sports day involving parents/guardians and children. Activities can include a three-legged race, human wheelbarrow, etc. Children can be involved in the promotion of the event to their families and relatives. See *joinusmoveplay.org/jump-fun-day-activities* for some ideas of activities that can be delivered as part of a fun day. To ensure all are motivated to take part, activities should be inclusive and avoid pressure and direct competition.

This toolkit is coproduced by Born in Bradford, Bradford Council, and Faith in Communities. For details, see introduction.

- **Monthly/termly theme:** Parents/guardians could get involved in a monthly/termly themed activity day such as the faith setting sessions around this sporting/physical activity, e.g. Olympics, cricket world cup, football world cup, etc.
- **Activities taster day:** Arrange taster sessions for children and parents with the aim of encouraging parents to continue this as a family activity.
- **Outdoor activities day:** Visit *joinusmoveplay.org/jump-outdoors* for inspiration.

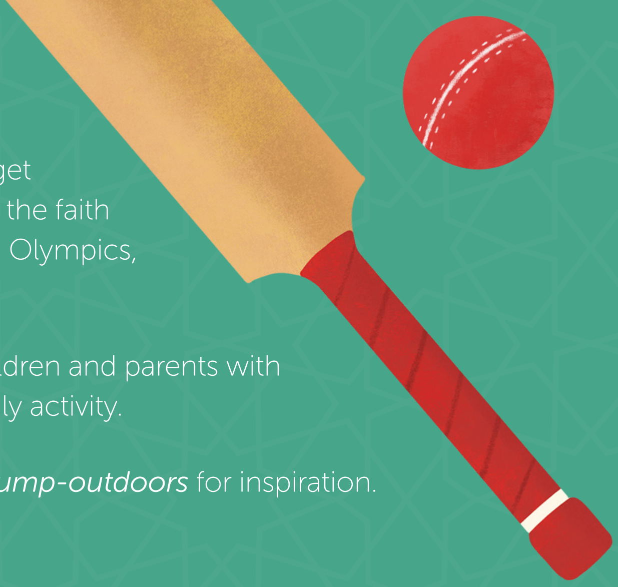

ACTIVITY 2
Developing a Plan

20 mins

Determine aims and actions for the introduction of fun days, considering what needs to be in place to ensure outcomes are effective and have a sustained impact on the participating children.

Below is an example of developing a plan for a fun day:

Aim	• To deliver an inclusive fun day with and for children and families
Actions	• Parents are approached to support the planning and delivery of fun day<br>• Workshop is held with volunteers and leaders to select activities, plan roles and responsibilities and organise logistics e.g. date<br>• Needs and resources for planning fun day are outlined, including further volunteers and equipment
What success looks like?	• An inclusive fun day is delivered, where children and parents contribute to the success of the event
Benefits for children and parents	• Children participate in a range of new activities that they can do in their own time<br>• Parents have ideas of games and activities to play with their children at home
Benefits for madrasas	• Fun days to be organised and repeated over time using the format developed<br>• Families are regularly engaged in physical activities together both at the faith setting and at home

This toolkit is coproduced by Born in Bradford, Bradford Council, and Faith in Communities. For details, see introduction.

We are family BORNINBRADFORD
JU:MP
FAITH IN COMMUNITIES
LIVING WELL
City of BRADFORD METROPOLITAN DISTRICT COUNCIL
Department of Health & Social Care
BRADFORD INSTITUTE FOR HEALTH RESEARCH | MAKING RESEARCH REAL
SPORT ENGLAND
Local Government Association

PHYSICAL ACTIVITY WORKSHOP 4

EXTRACURRICULAR AND LEISURE TIME

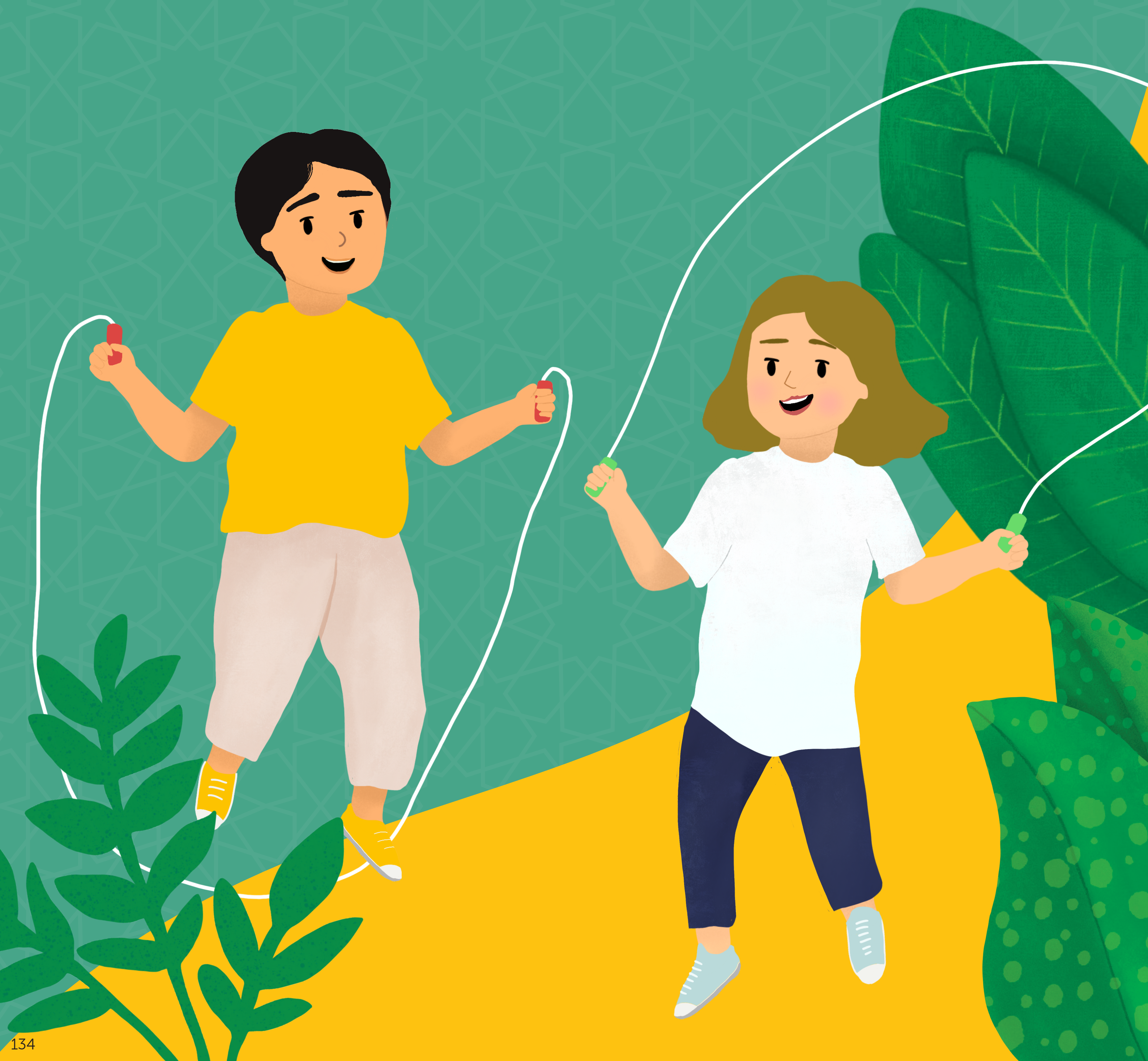

PHYSICAL ACTIVITY WORKSHOP 4

EXTRACURRICULAR AND LEISURE TIME

AIMS:

- To provide faith settings with different ideas about extracurricular physical activities that they can provide for children and families, and support them in planning these activities

OUTCOMES:

- Children, especially girls, have the opportunity to take part in physical activities that are fun, challenging and exciting for them

TARGET PARTICIPANTS:

- Parents, staff and volunteers

LENGTH OF WORKSHOP:

- 40 minutes

EQUIPMENT REQUIRED:

- Pens and paper

LOCATION:

- Faith setting

HEALTH AND SAFETY:

- N/A

FOOTWEAR AND CLOTHING:

- N/A

This toolkit is coproduced by Born in Bradford, Bradford Council, and Faith in Communities. For details, see introduction.

"And that there is nothing for the human being except what he/she strives [for]."
[Qur'an 53:39]

All of you are shepherds [responsible persons], and all of you will be asked about his flock [for whom he is responsible].
[Al-Bukhari]

Abu Huraira reported that the Prophet (pbuh) said, "Whoever goes to the mosque in the morning and evening, Allah will prepare for him a place in Paradise for every morning and evening."
[Al-Bukhari]

O you who believe! When the call is made for prayer on the day of congregation [Friday], hasten to the remembrance of Allah and leave off trade. That is better for you, if you but knew.
[Qur'an, 62:9]

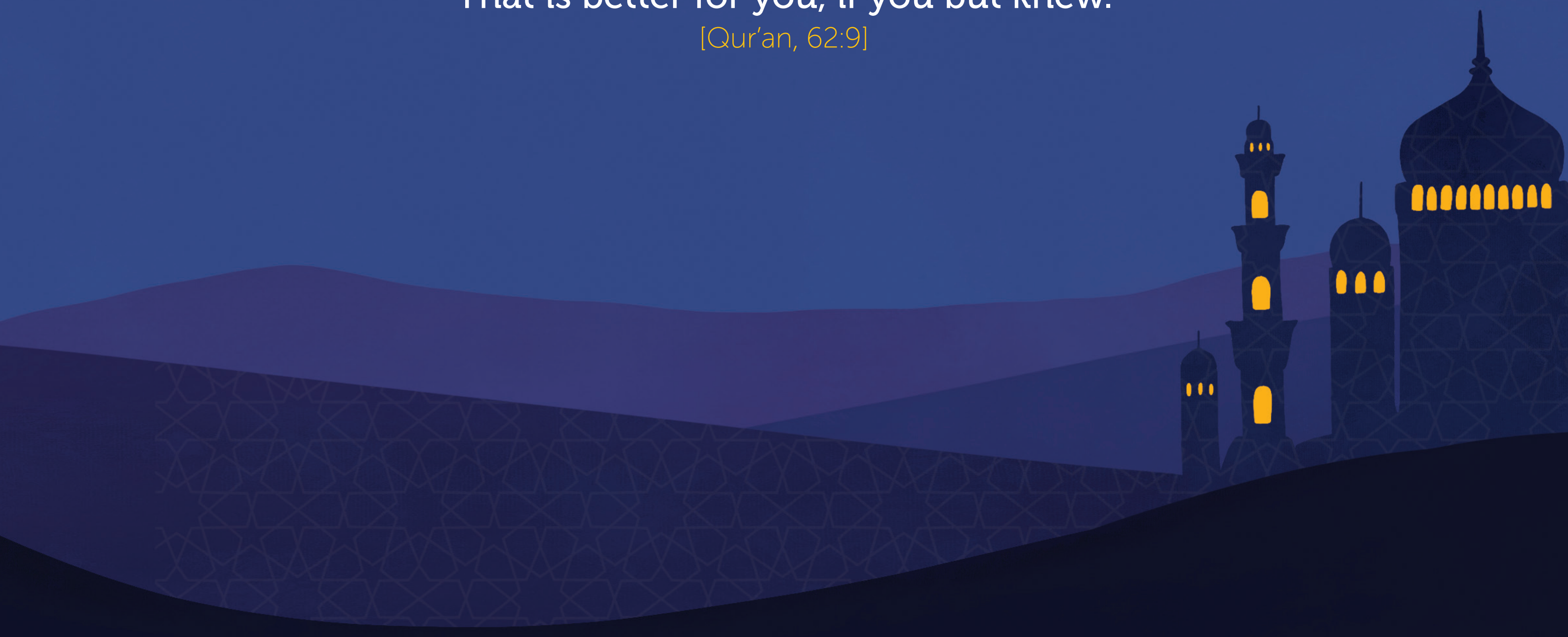

*City of Bradford Metropolitan District Council, and Born in Bradford do not own the Islamic Narrative in the toolkit and maintain this to be the independent work of Mufti Mohammed Zubair Butt. All enquiries relating to the Islamic Narrative should be referred to Mufti Mohammed Zubair Butt directly. For detailed disclaimer, please see page 2.

The Prophet (pbuh) told us that the entire process of attending the mosque, including every step taken to the house of God, is rewarded. "He who purifies himself (performs Wudhu) at his home and then walks to a house from the houses of Allah (mosque) so that he may discharge an obligation from the obligations of Allah (perform an obligatory prayer), one of his two step will wipe out a sin and the other (step) will elevate one rank (in Jannah)."

[Muslim]

In another Hadith, his cousin Ali described his manner of walking as follows: "When he walked, he lifted his leg with vigour. As though he was descending from a high place."

[Al-Mustadrak]

This toolkit is coproduced by Born in Bradford, Bradford Council, and Faith in Communities. For details, see introduction.

ACTIVITY 1
Discussion and Planning

40 mins

The facilitator explains that the faith setting could support various extracurricular physical activities.

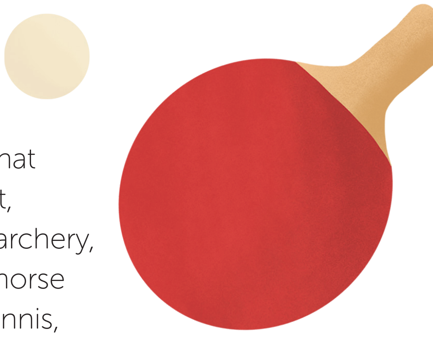

Action

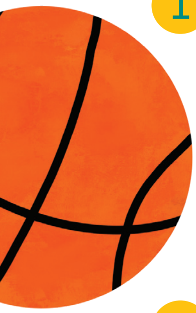

1. To give participants some ideas, consider the following list of activities that other faith settings have delivered: Skipping, baseball, ice hockey, cricket, rowing, dance, trampolining, muraqaba/yoga, badminton, gymnastics, archery, cycling, skateboarding, netball, wrestling, running, volleyball, rounders, horse riding, aerobics, field hockey, rugby, speedball, swimming, basketball, tennis, dodgeball, football, ice skating, pilates, canoeing-kayaking, table tennis, ju-jitsu, martial arts, circuit training, jogging, fencing, golf, exploring nature.

2. The facilitator encourages the group to discuss possible physical activities that the setting could support, and ask them to specifically consider which activities would be particularly appropriate for girls.

3. Choose at least one physical activity (or preferably more) to deliver, and plan to deliver this.

The following pages contain example physical activity session plans for archery, skipping, and exploring nature. These plans are provided as examples for facilitators in planning and managing physical activity sessions within the educational setting. The format can be adapted for any physical activity session that is appropriate for delivery by the facilitator within the educational setting.

These physical activities are options to be delivered either in educational settings or at the sites of specialist providers. It is highly recommended that these physical activity sessions are delivered by trained professionals with appropriate qualifications, or by specialist providers.

Please visit the Living Well website at *mylivingwell.co.uk* for signposting to local organisations and institutions that are recommended for the delivery of other physical activities.

To promote physical activity beyond the faith setting, Bradford Council offer regular guided walks across the countryside. Please visit *visitbradford.com/whats-on/guided-walks* for further information.

To be more active at home, visit *joinusmoveplay.org/campaigns/jump-home-2.*

1. Archery Session for Children

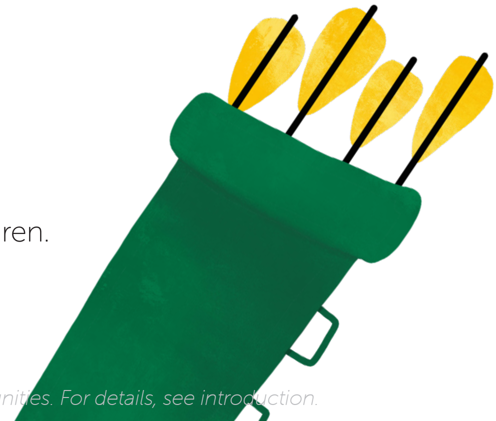

Planning:

1. Health group in faith setting plans an Archery session for children.

This toolkit is coproduced by Born in Bradford, Bradford Council, and Faith in Communities. For details, see introduction.

2. Recruit and enlist children/families who would like to join the session.
3. Link up with physical activity programmes in the area to secure resources, if needed.
4. Book with an archery instructor who can teach children the basic techniques of archery and help them to improve their aim.
5. Create a rota for volunteers to help on the day.

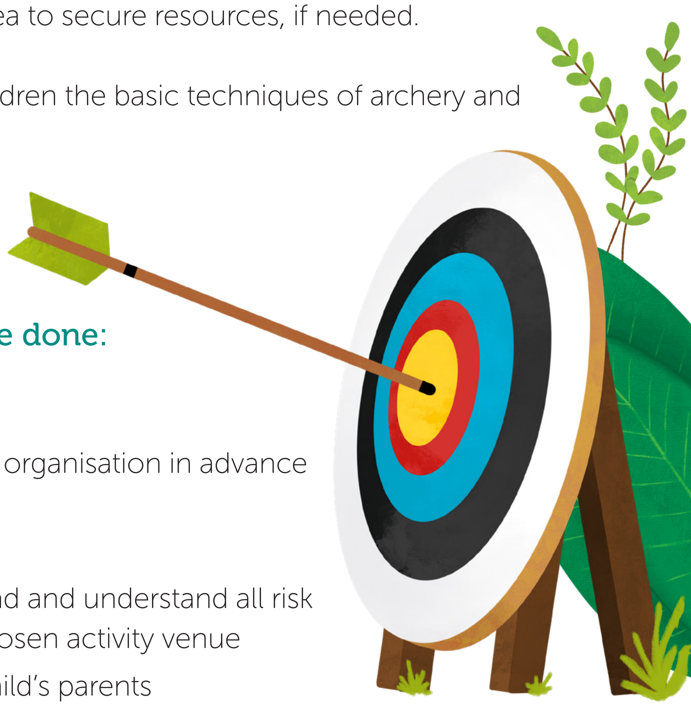

To prepare for the session(s) the following must be done:

1. Prior to the session:
   - All sessions should be booked with the chosen organisation in advance (see list of recommended providers)
   - Up to 8 children per group
   - Faith setting leaders should ensure that they read and understand all risk and health and safety procedures set by the chosen activity venue
   - A consent form must be completed by each child's parents
   - The faith setting should also have confirmation of any medical conditions of children
   - All children must be advised on conducting themselves in a courteous and respectful manner, as would be expected of them if they were in a faith setting
   - Children should also be informed of any rules they must follow for Health and Safety purposes

2. Preparation on the day of sessions:
   - It is easier if parents drop off the children and take them home
   - A register must be taken on arrival (signed by parents) and also signed prior to the child/children leaving
   - Recap Health and Safety rules before session begins (although this is likely to be covered by the Archery instructor)

Archery clubs
- Bradford University Archery club: *bradfordunisu.co.uk/groups/archery-2dce*
- National Archery Bradford: *bit.ly/3JhBK93*
- Yorkshire Archery Association: *www.yorkshirearchery.co.uk/beginning.html*

2. Skipping

Planning:

1. Health group in faith setting plans and organises a Skipping session for children.
2. Recruit children/families who would like to join the session.

3. Link up with a physical activity programme in the area to secure resources, if needed.

4. Book a skipping instructor who can teach children the basic techniques of skipping and help them to improve their skills.

5. Create a rota for volunteers to help on the day.

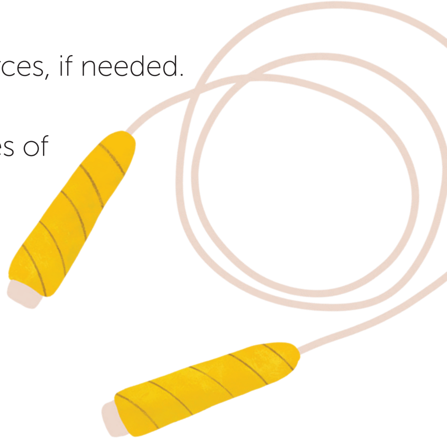

Warm-up:

The warm-up should not be vigorous, as the purpose is only to raise the heart rate so that the body is warmer and more flexible, allowing exercises to be performed in a safer manner.

Instructors to demonstrate the correct way to do each exercise before asking children to participate.

- **Light jogging on the spot:** Demonstrate running on the spot. Ask the children to join in and jog with them for 1 minute. Ask children to run in good form to help reduce injuries.
- **Rotating arms:** Stand straight with legs shoulder width apart then raise the right arm straight up above the head. Slowly rotate the arm forward down past the right leg and back up to original starting position. Repeat exercise for 30 seconds before changing to the other arm.
- **Knee ups:** Start as if demonstrating light jogging on the spot then hold out both hands at 90 degrees in front of the body (waist height) with palms facing down. Continue to jog on the spot but start raising knees so they touch the palms of the hands every time. Do this for one minute.

Ensure that all children have enough space (2 metres squared) between each other to avoid accidents. Each child is given a skipping rope each.

Main Activity:

- **Slow motion skipping:** Demonstrate how to stand upright holding onto the skipping rope with elbows close to the body. Rope should be behind feet. Slowly use both hands to swing the rope over the body until it lands in front of the feet, then jump over the rope with both feet together. Ask children to repeat this until they can do it easily.
- **Skipping:** Next, show children how the slow-motion skipping can be changed into regular skipping by speeding up the actions. Children should be encouraged to anticipate the rope coming down to their feet and try to jump before the rope touches the ground. Moves should be continuous so the rope should not stop at the feet as in slow-motion skipping. After children have managed to skip over the rope once or twice, encourage them to set their own target of how many times they can skip over the rope without the rope getting caught.
- **Double skip:** This will start off in the same position as regular skipping but will require a higher jump so that the skipping rope can be passed twice whilst the legs are in the air. See YouTube link for further information: *youtube.com/watch?v=Id5RG86CSg4*
- **Cross skip:** You must use your wrists when completing this exercise, and ensure the rope goes over your head when completing the move. This starts as a regular skip but when the

This toolkit is coproduced by Born in Bradford, Bradford Council, and Faith in Communities. For details, see introduction.

hands are at eye level, the arms need to cross over (in front of the chest) before the rope reaches the ground.

- **Freestyle skip:** Children are allowed to use what they have learnt and skip without any specific routine.

Cool down:

- **Slow motion skip to the wall:** Children are asked to stand at one end of the room and pretend they are slow motion skipping to the other end of the room.

3. Exploring Nature

Planning:

1. Health group in faith setting plans an Exploring Nature activity for children and families.
2. Recruit and enlist children/families who would like to join in a day out in nature.
3. Link up with a physical activity programme in the area to secure resources, if needed.
4. Identify staff/volunteers/parents and a physical activity instructor who can teach children the basic techniques of walking in nature and help them to improve their skills.
5. Create a rota for volunteers to help on the day.

To prepare for the session(s) the following must be done:

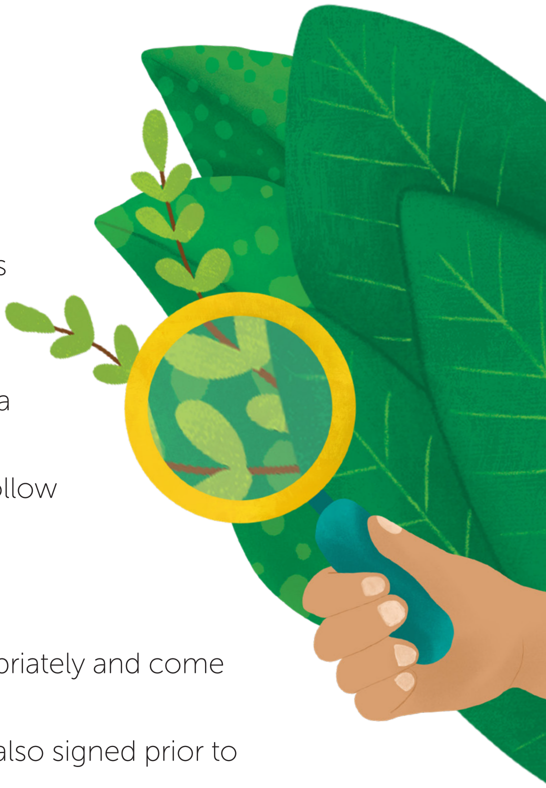

1. Prior to the session:
   - A consent form must be completed by each child's parents
   - Very young children may not be permitted to attend. You must check with the centre in relation to age restrictions
   - All children must be advised on conducting themselves in a courteous and respectful manner
   - Children should also be informed of any rules they must follow for Health and Safety purposes
2. Preparation on the day of session:
   - It is important to ensure that all children are dressed appropriately and come prepared for a change in weather
   - A register must be taken on arrival (signed by parents) and also signed prior to the child/children leaving
   - Recap Health and Safety rules before session begins (although this is likely to be covered by the specialist instructor)

This toolkit is coproduced by Born in Bradford, Bradford Council, and Faith in Communities. For details, see introduction.

Main Activity:

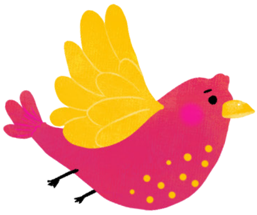

We provide one example below, of exploring nature at Bolton Abbey in the Yorkshire Dales. Feel free to choose any location that would allow children and families to be in nature, and adapt the activities below accordingly.

Bolton Abbey is an amazing place that provides a great deal of exposure to the joys of nature, such as discovering wildflowers, and seeing wildlife such as otters and deer in the woods. Age and ability appropriate activities can be selected from the list below by faith setting staff on the day:

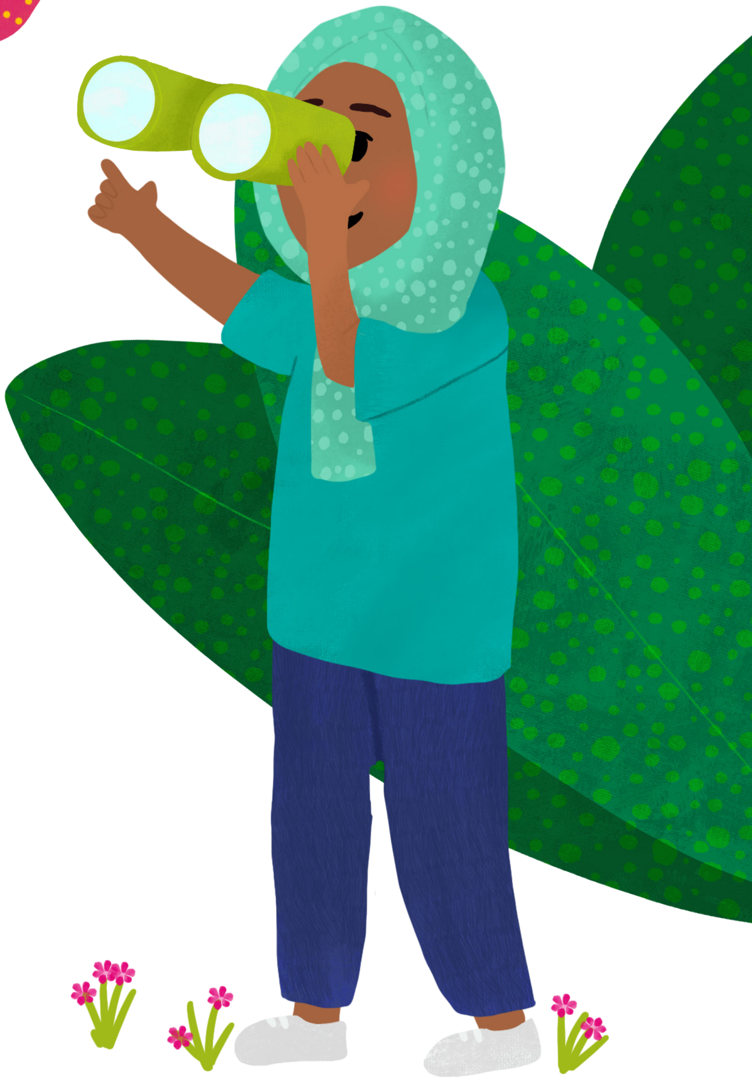

- **Stepping Stones:** These are 60 stones that are safe to cross, and will help to improve the balance of children (children aged under 7 should always be supervised by an adult). Please note any children or adults not able to cross the water via the stones can cross over via the bridge.
- **Beach:** There is a large beach area which is family friendly and suitable for very young children, this is located north of the stepping stones
- **Barden Moor and Barden Fell:** This is a walk that leads to a beautiful waterfall. This walk is only recommended for children 12+ so as recommended it would be appropriate to split groups into separate age groups. The walk also leads on to Simon’s Seat, which has an amazing view. This may be a good moment for children to take time and admire the beauty of nature and link it back to how we should look after the environment, to preserve nature.
- **Striding Woods:** The nature trail through the woods has various different nature trails which are colour coded. These trails should not be too challenging for children aged 7+, but staff members should be aware that children will be of differing abilities.
- **Child friendly activities:** Throughout the walks there are various challenges for children to engage in, such as mazes and slides. These will keep children engaged and enthusiastic.
- **Extra considerations - Wheelchair friendly:** Striding Woods has a nature trail which is labelled green, this is a wheelchair friendly path. This is a reminder that, despite whatever challenges human beings face, there are means to access nature. This can teach children about resilience and having faith and showing patience. It is a good reminder for us all to appreciate what we have at our doorstep and always try to look at the positives. A good opportunity to encourage children to be more resilient.

For ideas of other green spaces to visit, please visit *bit.ly/3Ns8crL* for a list of 25 self-guided walks around Airedale. Detailed instructions and a map are provided for each walk.

We are family BORNINBRADFORD | JU:MP | FAITH IN COMMUNITIES | LIVING WELL | City of BRADFORD METROPOLITAN DISTRICT COUNCIL

Department of Health & Social Care | BRADFORD INSTITUTE FOR HEALTH RESEARCH MAKING RESEARCH REAL | SPORT ENGLAND | Local Government Association

PHYSICAL ACTIVITY WORKSHOP 5

BEING ACTIVE THROUGH TRAVEL

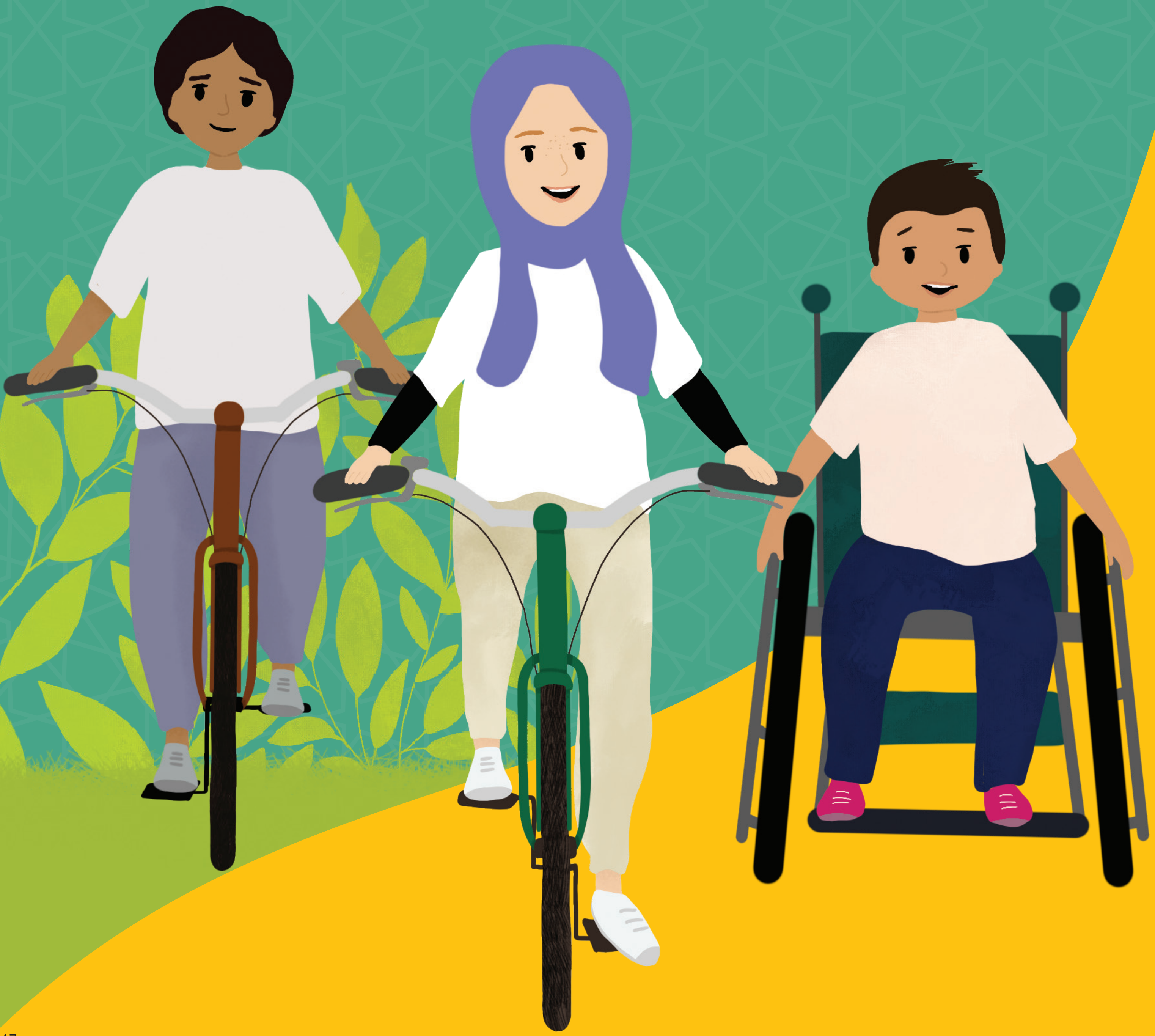

PHYSICAL ACTIVITY WORKSHOP 5

BEING ACTIVE THROUGH TRAVEL

AIMS:

- To develop a plan for increasing walking to/from faith setting
- Support parents and families to commit to walking rather than driving

OUTCOMES:

- Fewer cars outside the faith setting at pick up and drop off times
- An increased understanding of road safety and precautions when travelling by foot
- More children and families travel actively

TARGET PARTICIPANTS:

- Children and parents, staff and volunteers

LENGTH OF WORKSHOP:

- 1 hour

EQUIPMENT REQUIRED:

- N/A

LOCATION:

- Faith setting (also getting to and from)

HEALTH AND SAFETY:

- Safety for children (especially around roads) when walking.
- Road safety on planned route, safeguarding of children when walking as part of a group (e.g. maximum number of children per volunteer leading group).

FOOTWEAR AND CLOTHING:

- Appropriate outdoor clothing for walking to madrasa for the weather conditions

NOTE: This workshop complements **Healthy Places Workshops 5** and **6**. Facilitators may want to consider running these sessions consecutively.

In this workshop children should be involved in activities 1, 2 and 4. You may want to consider providing some physical activities for them to take part in whilst their parents and staff/volunteers continue to complete activity 3.

This toolkit is coproduced by Born in Bradford, Bradford Council, and Faith in Communities. For details, see introduction.

"And do not throw [yourselves] with your [own] hands in to destruction."
[Qur'an, 2:195]

[There are] two blessings in which many people are deceived: good health and free time.
[Al-Bukhari]

Abu Huraira reported that the Prophet (pbuh) said, "Whoever goes to the mosque in the morning and evening, Allah will prepare for him a place in Paradise for every morning and evening."
[Al-Bukhari]

O you who believe! When the call is made for prayer on the day of congregation [Friday], hasten to the remembrance of Allah and leave off trade. That is better for you, if you but knew.
[Qur'an, 62:9]

*City of Bradford Metropolitan District Council, and Born in Bradford do not own the Islamic Narrative in the toolkit and maintain this to be the independent work of Mufti Mohammed Zubair Butt. All enquiries relating to the Islamic Narrative should be referred to Mufti Mohammed Zubair Butt directly. For detailed disclaimer, please see page 2.

The Prophet (pbuh) told us that the entire process of attending the mosque, including every step taken to the house of God, is rewarded.
"He who purifies himself (performs Wudhu) at his home and then walks to a house from the houses of Allah (mosque) so that he may discharge an obligation from the obligations of Allah (perform an obligatory prayer), one of his two step will wipe out a sin and the other (step) will elevate one rank (in Jannah)."

[Muslim]

In another Hadith, his cousin Ali described his manner of walking as follows: "When he walked, he lifted his leg with vigour. As though he was descending from a high place."

[Al-Mustadrak]

This toolkit is coproduced by Born in Bradford, Bradford Council, and Faith in Communities. For details, see introduction.

ACTIVITY 1
Discussion

10 mins

To begin the session, the facilitator will encourage children and parents to contribute to a discussion on the current barriers that they face, which are preventing them from walking to and from the faith setting.

These might include:

- Lack of time
- Busy roads
- Poor weather conditions

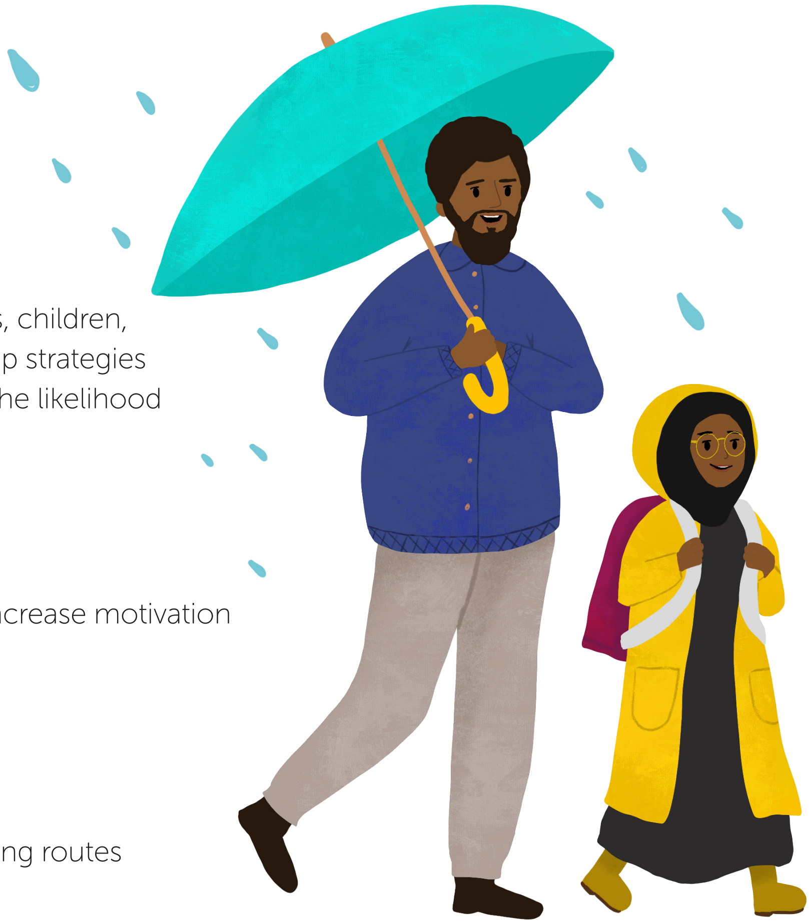

Based on the barriers identified, parents, children, and facilitators work together to develop strategies to overcome these issues, to increase the likelihood of adopting active travel.

Some examples may include:

- Road safety sessions for families
- Incentives for walking families to increase motivation
- Walking buses
- Park and stride
- Traffic control volunteers
- No-parking zones
- Mapping recommended safe walking routes

ACTIVITY 2
Creative Session

10 mins

Children and young people taking part in the workshop advocate through stories or drawings about environmental, social and health benefits of reducing reliance on cars.

ACTIVITY 3
Walking Bus

30 mins

This activity facilitates the creation of a 'walking bus' to and from the faith setting. Read through the steps below, discuss each step and decide who will take responsibility for actioning each step so that a 'walking bus' can happen regularly.

1. Faith setting health group contacts parents and brings them on board.

This toolkit is coproduced by Born in Bradford, Bradford Council, and Faith in Communities. For details, see introduction.

2. Health group considers/decides on response for parents/children who want to bring (for example) scooters or bicycles to the walking group. Discuss if this will be facilitated/possible, and how it can be managed by staff.

3. Health group identifies staff/volunteers to walk children to the faith setting.

4. Children and families are recruited for walking (this can include identifying all families living within one location or area that could form one group, with families from other areas forming another group where appropriate).

5. Health group makes a rota for the implementation of a 'walking bus' to the faith setting.

6. Faith setting to appoint a dedicated staff member/volunteer (e.g. 'Lollipop Imam') to change rota for walking children from home to the faith setting.

7. If appropriate, children can be involved in planning the route and identifying friends who attend the faith setting from the same area that could also be part of the group.

Optional: For families who drive to the faith setting because they live further than a feasible walking distance, the health group can identify a nearby area (e.g. a car park) for pick-up/drop-off, to allow children the opportunity for a short walk from the car to the faith setting, and vice versa.

Optional: Faith setting staff can incentivise active travel to the faith setting through a rewards system. For example, if children actively travel to the faith setting X times per month, or hit a particular step count in a week, they may receive a reward. Rewards can be tailored depending on the children's ages.

ACTIVITY 4
Discussion: Health and Safety

10 mins

The facilitator leads a discussion with all workshop participants on health and safety considerations when walking to the faith setting. The facilitator can ask, "What would keep you safe whilst walking or travelling actively to the faith setting?"

The discussion should include the following road safety considerations:

- Wear light-coloured or high-viz reflective jackets, particularly if late at night or in winter, when it is dark early
- Whenever possible, use pedestrian crossings for crossing roads
- Crossing roads and awareness of vehicles (including consideration of electric vehicles which can be quieter and may not be heard as much in advance)
- Safety considerations in using headphones/earphones, or mobile phones when walking
- Being part of a group increases safety for children and is safer than walking alone, particularly when it is dark
- If children bring scooters to the walking group, these should be used on pavements, and extra caution taken when crossing roads

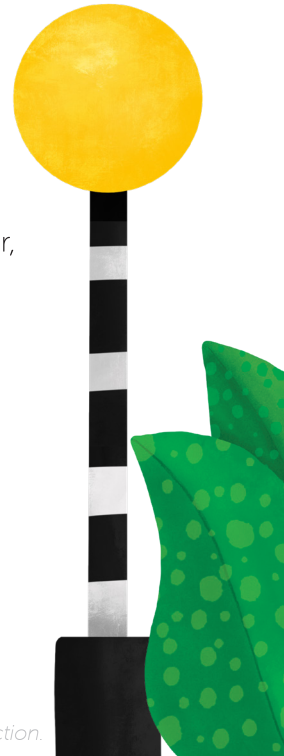

This toolkit is coproduced by Born in Bradford, Bradford Council, and Faith in Communities. For details, see introduction.

PHYSICAL ACTIVITY WORKSHOP 6

USING ISLAMIC NARRATIVE TO SUPPORT PHYSICAL ACTIVITY

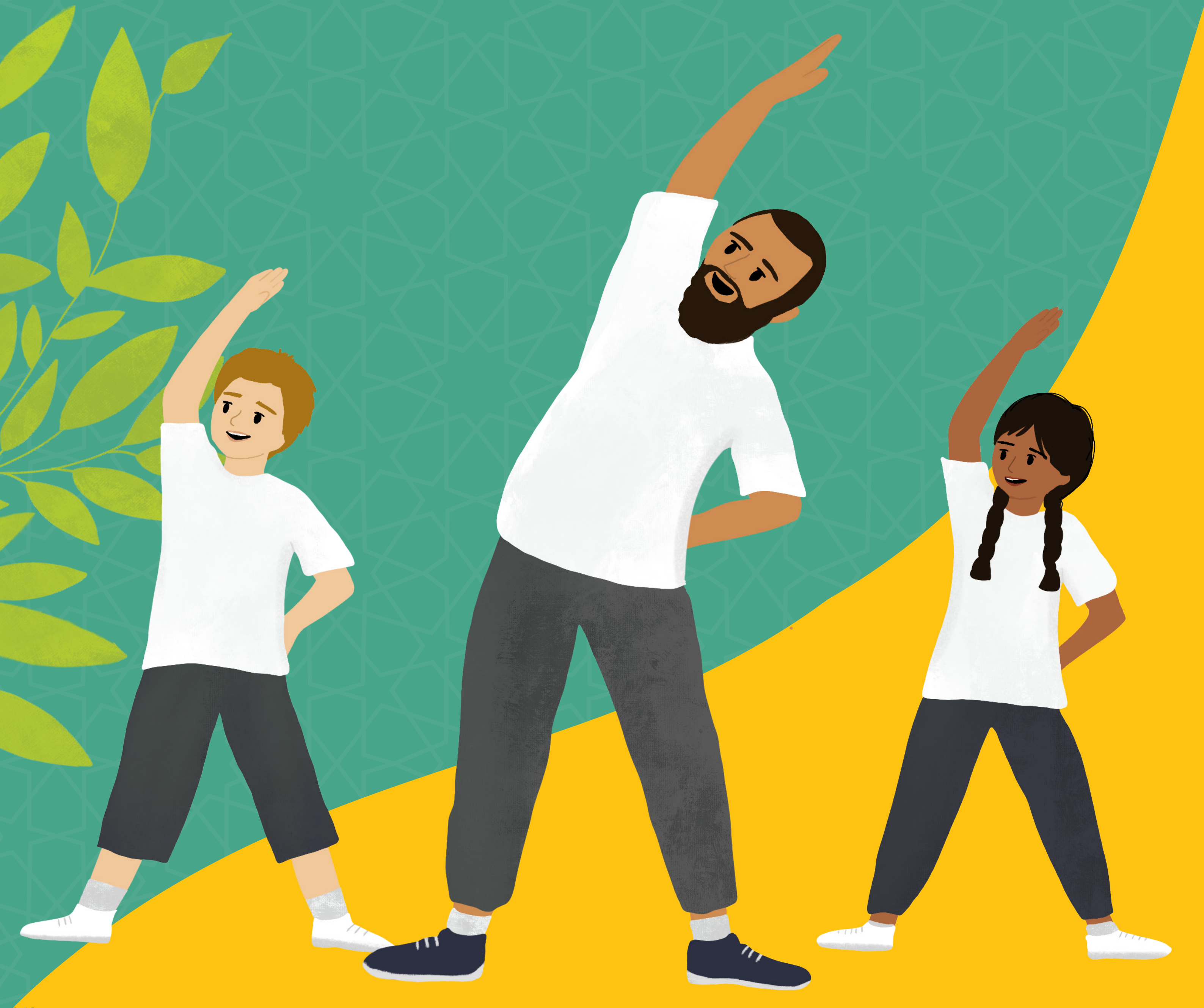

PHYSICAL ACTIVITY WORKSHOP 6

USING ISLAMIC NARRATIVE TO SUPPORT PHYSICAL ACTIVITY

AIMS:

- To widen knowledge of Islamic narratives for physical activity for the community
- To normalise physical activity for all participants

OUTCOMES:

- Faith setting leaders and management have a plan and are confident in how to use an Islamic narrative to support physical activity messages across faith setting activities and teachings
- Physical activity is embedded as part of the faith setting offering

TARGET PARTICIPANTS:

- Imam, Islamic leaders, management

LENGTH OF WORKSHOP:

- 1 hour and 25 minutes

EQUIPMENT REQUIRED:

- N/A

LOCATION:

- Madrasa

HEALTH AND SAFETY:

- N/A

FOOTWEAR AND CLOTHING:

- N/A

This toolkit is coproduced by Born in Bradford, Bradford Council, and Faith in Communities. For details, see introduction.

ISLAMIC NARRATIVE

"Let there be a group among you who invite to all that is good..."
[Qur'an, 3:104]

Whilst offering advice to a man, the Prophet Mohammed (pbuh) said, "Value five [things] before five [other things]: Your youth before your old age, your health before your illness, your affluence before your poverty, your availability before your occupation and your life before your death."
[Al-Mustadrak]

One of the reasons Talut (Saul), who was then a soldier, was made king over the children of Israel, despite his poverty and despite not being a descendent of the house of kings, was his bodily strength.
[Al-Tafsir al-Kabir]

Verily, Allah has chosen him [Talut] above you and has increased him abundantly in knowledge and stature.
[Qur'an, 2:247]

The prophet Moses was also lauded for his bodily strength."One of the two women said, 'O my father, hire him. Indeed, the best whom you might hire is the strong and the trustworthy.'"
[Qur'an, 28:26]

*City of Bradford Metropolitan District Council, and Born in Bradford do not own the Islamic Narrative in the toolkit and maintain this to be the independent work of Mufti Mohammed Zubair Butt. All enquiries relating to the Islamic Narrative should be referred to Mufti Mohammed Zubair Butt directly. For detailed disclaimer, please see page 2.

It is narrated from Abu Huraira that the Messenger of Allah (pbuh) said, “The strong believer is better and more beloved to Allah than the weak believer. And in each is good. Covet that which gives you benefit [in this world and in the afterlife] and seek help in Allah and do not be weak.”

[Muslim]

The strength and weakness mentioned in this Hadith is undoubtedly a reference, primarily, to strength and weakness in belief, and so a person with strong faith is better than a person with weak faith. However, it is also a reference to internal resolve that encourages a person to discharge their religious obligations and, according to some commentators, to bodily strength that allows one to better perform such obligations.

The Prophet Muhammad (pbuh) would naturally walk at a vigorously brisk pace and his close companions found it difficult to keep pace with him. One of his close companions, Abu Huraira, described his walking on one occasion saying, “And I did not see anyone faster in his manner of walking than the Messenger of Allah pbuh, as if the earth was folded for him. We would exert ourselves [to keep up with him] whilst he was not troubled.”

[Ibn Hibban]

In another Hadith, his cousin Ali described his manner of walking as follows: “When he walked, he lifted his leg with vigour. As though he was descending from a high place.”

[Al-Mustadrak]

It is narrated from Jabir, “Some people complained to the Prophet (pbuh) of [weakness and tiredness in] walking so he called for them and said, ‘You should most certainly take up brisk walking.’ So we took up brisk walking and found it easier for us.”

[Al-Mustadrak]

This toolkit is coproduced by Born in Bradford, Bradford Council, and Faith in Communities. For details, see introduction.

ACTIVITY 1
Discussion

15 mins

A discussion is led by the Imam who explains, discusses and educates participants on available Islamic narratives to encourage physical activities.

Suggested Islamic narratives that can be drawn upon are listed on the previous two pages.

ACTIVITY 2
Implementing Islamic Narratives

30 mins

Facilitator to lead on an action plan to compile Islamic narratives and implement into the faith setting.

Action:

1. The facilitator asks all participants to prepare a comprehensive list of Islamic narratives that they are familiar with, on encouraging physical activity.
2. Faith setting staff then plan on embedding the Islamic narrative into their curriculum to teach children the selected Islamic narratives on physical activity.
3. Faith setting staff make a schedule to teach/familiarise children with the Islamic narratives on physical activity.
4. Faith setting staff teach children to learn the saying of the Prophet (pbuh) on spiritual reward for every step taken whilst walking to the faith setting, and to encourage children to share the saying with parents.
5. The health group searches for and collects published and online material that includes the Islamic narratives on physical activity, and makes it a part of the faith setting library.

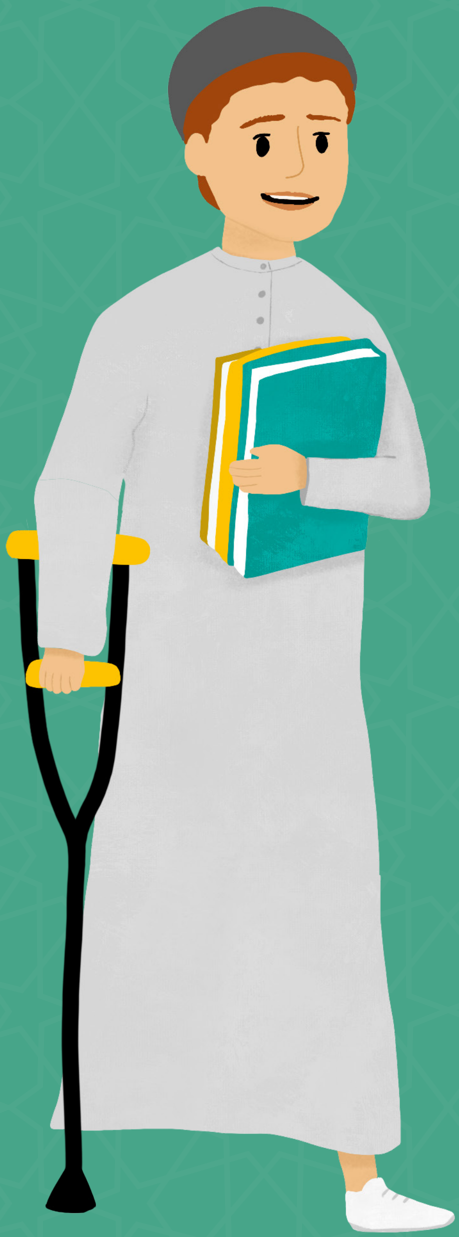

ACTIVITY 3
Friday Sermon

20 mins

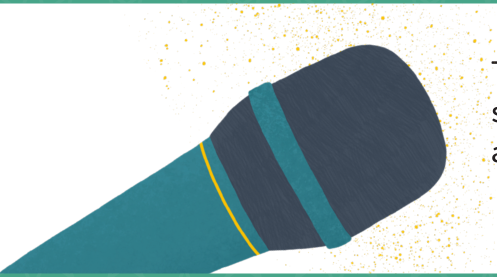

The health group and Imam plan and schedule a dedicated Friday sermon on sharing the Islamic narrative on encouraging physical activity, which has been collated in Activity 2.

ACTIVITY 4
Strategic Planning

20 mins

The facilitator leads the health group in planning how to use an Islamic narrative strategically, for physical activity promotion across the year. This should also include how to ensure that families, particularly women and girls, are also involved.

Action:

1. If it has not already been part of the discussion, the facilitator can introduce the idea of building physical activity into the faith setting curriculum of Islamic history (Sunnah foods and sports).
2. The facilitator should encourage participants to discuss how to embed physical activity as part of the faith setting offering, so that it is there regularly, rather than as a one-off discussion or event.
3. Participants share ideas.

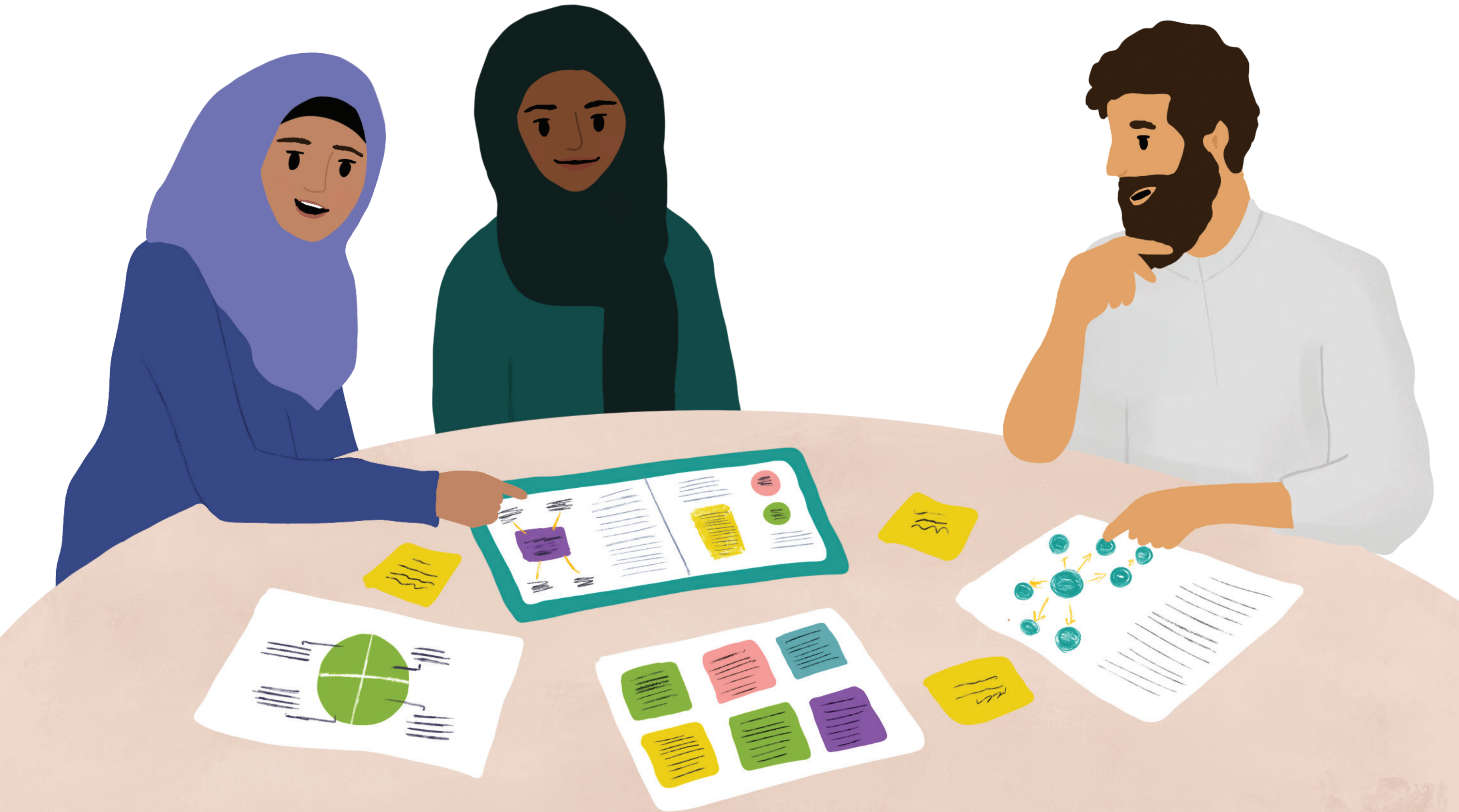

FURTHER INFORMATION AND RESOURCES FOR FACILITATORS

HEALTHY PLACES

Delivering a childhood obesity prevention intervention using Islamic religious settings in the UK: What is most important to the stakeholders?" Preventive medicine reports 22 (2021): 101387
Dogra, Sufyan Abid, Kiran Rai, Sally Barber, Rosemary RC McEachan, Peymane Adab, and Laura Sheard.
doi.org/10.1016/j.pmedr.2021.101387

Excellence in Mosques CD Toolkit
www.icea.org.uk/announcement/excellence-in-mosques-cd-toolkit

Public Health Matters: Tackling Health Inequalities through Enhancing the Wellbeing Role of Mosques
bit.ly/3NRLb1S

Healthy Living: Mosques
www.gov.uk/government/publications/healthy-living-mosques

9 Things You Didn't Know About The Prophet's Mosque
bit.ly/3NsRV4P

Towards Eco-Friendly Socio-Economic Mosques
www.islamicity.org/18154/eco-friendly-socio-economic-mosques

ROSPA: Issues that Schools Should Consider in Relation to School Transport
www.rospa.com/school-college-safety/teaching-safely/school-transport

HEALTHY DIET

NHS: Managing Your Weight
www.nhs.uk/live-well/eat-well/cut-down-on-your-calories

British Nutrition Foundation: A Healthy Ramadan
www.nutrition.org.uk/healthyliving/seasons/ramadan.html

NHS Better Health, Healthier Families: Easy Ways to Eat Well and Move More
www.nhs.uk/change4life

Food Standards Agency
www.food.gov.uk

BDA - The Association of UK Dieticians: Food Facts
www.bda.uk.com/food-health/food-facts.html

British Heart Foundation: Food Portions
bit.ly/3ph0ZSh

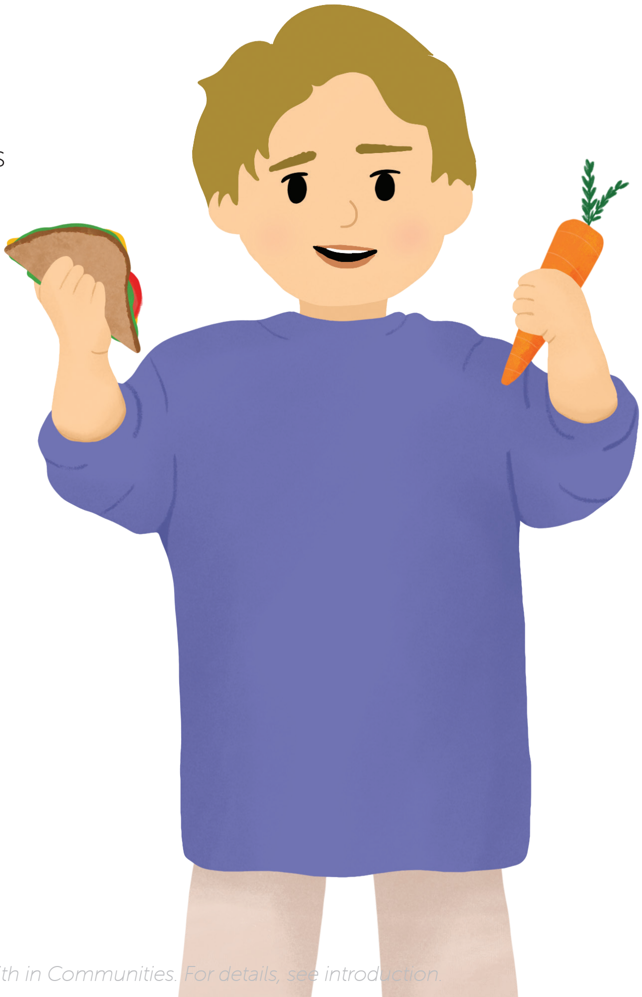

This toolkit is coproduced by Born in Bradford, Bradford Council, and Faith in Communities. For details, see introduction.

Living Well: Why is it Important to Eat Well?
mylivingwell.co.uk/eating-well

Don't Like Salad: Tony Ross
bit.ly/3CMgKUn

Pancakes Pancakes: Eric Carle
bit.ly/44jHdnX

How Did That Get in My Lunchbox? The Story of Food: Chris Butterworth
https://amzn.to/48qZEJR

See Inside: Where Food Comes From: Emily Bone
https://amzn.to/47BNVGV

PHYSICAL ACTIVITY

One Path Network: Revive Ramadan – Park further away from the Masjid
onepathnetwork.com/revive-ramadhan-park-further-away-from-the-masjid

NHS: Physical Activity Guidelines for Children and Young People
www.nhs.uk/live-well/exercise/physical-activity-guidelines-children-and-young-people

Join Us Move Play: Activities to do at home
joinusmoveplay.org/jump-home

Living Well: Why is it Important to be Physically Active ?
mylivingwell.co.uk/physical-activity

Faith in Communities CIC, Bradford
faithincommunities.co.uk

Bolton Abbey: What to See and Do
boltonabbey.com/what-to-see

Child Mind Institute: Why Kids Need to Spend Time in Nature
https://bit.ly/3Q0kJTq

Bradford Council: Road Safety
bso.bradford.gov.uk/content/road-safety

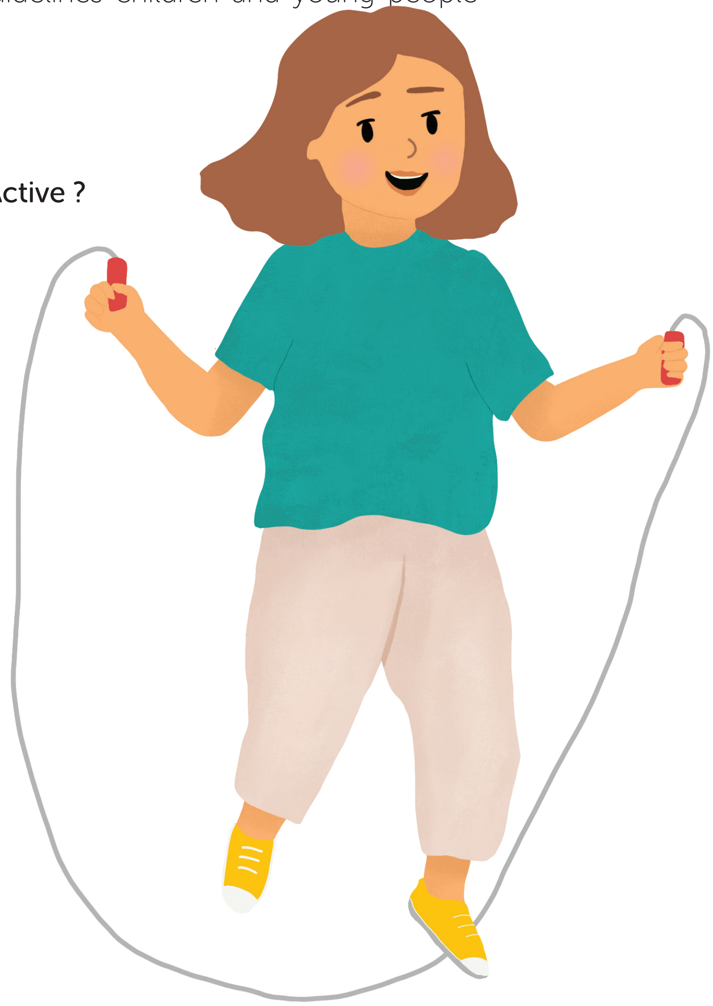

This toolkit is coproduced by Born in Bradford, Bradford Council, and Faith in Communities. For details, see introduction.

bib We are family BORNINBRADFORD

JU:MP

FAITH IN COMMUNITIES

LIVING WELL

City of BRADFORD METROPOLITAN DISTRICT COUNCIL

Department of Health & Social Care

BRADFORD INSTITUTE FOR HEALTH RESEARCH

MAKING RESEARCH REAL

SPORT ENGLAND

Local Government Association
